# Exogenous tryptophan aggravates experimental lung fibrosis through activation of AKT-mTORC1 pathway

**DOI:** 10.1371/journal.pone.0361463

**Published:** 2026-11-03

**Authors:** Jisu Hong, Yunha Nam, Jib Yong Jung, Jun Yeong Baek, Eun Ji Park, Yemin Choi, Shinhee Park, Ae-Rin Baek, Hun-Gyu Hwang, Junehyuk Lee, An-Soo Jang, Do-Jin Kim, Su Sie Chin, Woo Jin Kim, Sung-Woo Park

**Affiliations:** 1 Division of Allergy and Respiratory Medicine, Department of Internal Medicine, Soonchunhyang University Bucheon Hospital, Bucheon, South Korea; 2 Department of Pathology, Soonchunhyang University Bucheon Hospital, Gyeonggi-Do, South Korea; 3 Department of Internal Medicine Environmental Health Center, Kangwon National University, Chuncheon-si, Gangwon-do, South Korea; Longgang Otorhinolaryngology Hospital & Shenzhen Key Laboratory of Otorhinolaryngology, Shenzhen Institute of Otorhinolaryngology, CHINA

## Abstract

Idiopathic pulmonary fibrosis (IPF) is marked by aberrant lung remodeling and excessive extracellular matrix (ECM) accumulation. Previously, we observed elevated tryptophan (TRP) levels in the lungs of IPF patients, but its role in fibrogenesis remained unclear. This study examines whether TRP modulate the severity of lung fibrosis by measuring ECM component expression in TRP-treated lung fibroblasts. The activities of key TRP metabolism enzymes, tryptophan hydroxylase (TPH-1) and indoleamine 2,3-dioxygenase (IDO/TDO), were also assessed in IPF lung. Exogenous TRP administration in bleomycin-exposed mice significantly exacerbated lung damage and fibrosis. TPH-1 levels were elevated in lung tissues and primary fibroblasts of IPF patients compared to controls, while IDO/TDO expression showed no significant differences between two groups. TRP treatment increased fibroblast proliferation and upregulated collagen, fibronectin, and α-smooth muscle actin expression, whereas TRP deprivation reduced these ECM component levels. Additionally, TRP upregulated the AKT-mTORC1 and TGF-β1 signaling pathways in IPF fibroblasts. These findings indicate that elevated TRP levels contribute to IPF pathogenesis and suggest that targeting the TRP pathway could offer therapeutic potential for lung fibrosis including IPF.

## Introduction

Idiopathic pulmonary fibrosis (IPF) is a chronic progressive lung disease characterized by alveolar epithelial cell hyperplasia, increased numbers of myofibroblasts, the interstitial deposition of extracellular matrix (ECM), and a remodeling of the lung architecture [1–3]. The accumulation of heterogeneously distributed ECM components in the alveolar parenchyma is a key feature of IPF [4,5]. The ECM is an interlocking mesh of fibrous proteins and glycosaminoglycans, but the most abundant ECM component in most tissues is collagen [6]. However, a detailed mechanistic understanding of the development or progression of IPF is still lacking and the available therapeutic options are poorly effective, as antifibrotic agents slow lung function decline but they fail to produce stable benefits [7–9]. Elucidation of the precise mechanisms of IPF and the development of targeted drugs therefore remain high priorities.

L-Tryptophan (TRP) is an essential amino acid and the precursor of the neurotransmitter serotonin (5-hydroxytryptamine [5-HT]), which regulates gastrointestinal functions, mood, and appetite [10,11]. Recent studies have shown that L-kynurenine and 5-HT, as key metabolites of TRP, modulate fibroblast activation *in vitro* and in experimental fibrosis [12]. Kynurenine or its pharmacological agonist attenuate fibroblast proliferation whereas 5-HT exhibits profibrotic activity in various fibrosis models [12,13]. 5-Methoxytryptophan (5-MTP), a newly found TRP metabolite, inhibits the fibrotic response both *in vitro* and in fibrosis models by downregulating TGF-β1 activity [14,15]. Recently, using a metabolomic approach, we reported significantly higher lung levels of TRP in IPF patients than in controls [16], but the exact role of TRP in IPF was not determined (Fig 1a).

**Fig 1:**
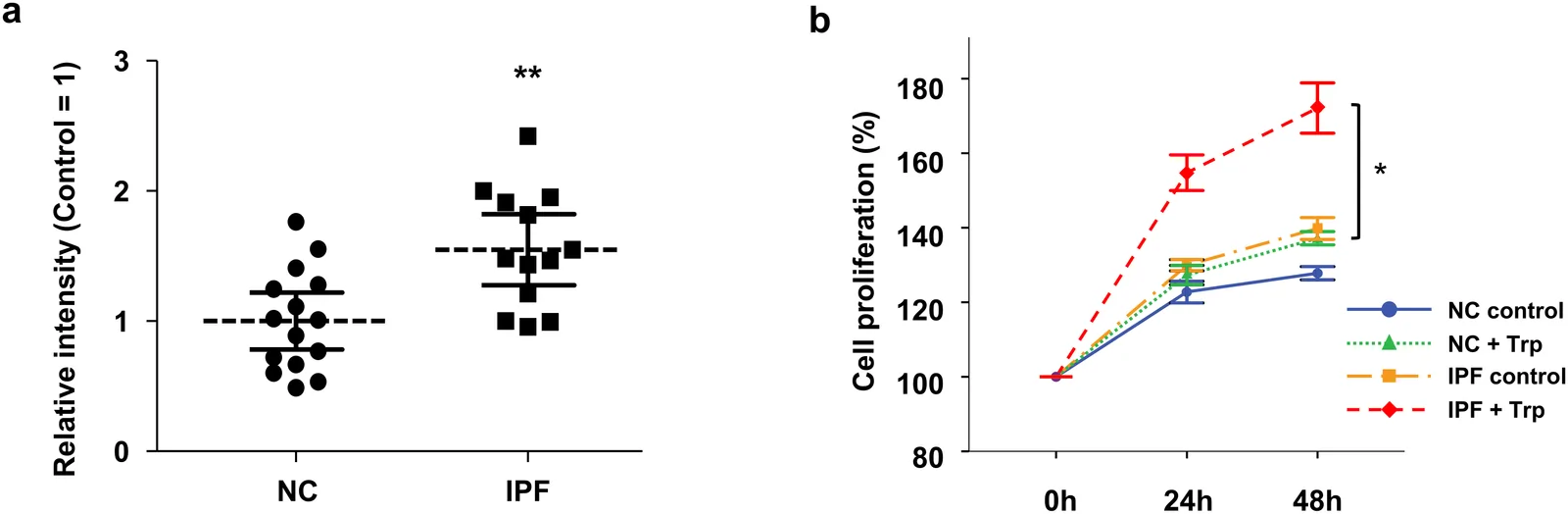
Tryptophan (TRP) levels in the lung are elevated in idiopathic pulmonary fibrosis (IPF), and the proliferation of IPF fibroblasts is significantly increased by TRP. a. TRP levels in NC (n = 15) and IPF (n = 13) lung tissues were obtained from our previous metabolomic study [16]. Values are presented as the mean with 95% confidence intervals. **p < 0.01 vs. the control group. b. Proliferation of control and IPF fibroblasts treated with or without 10 µM TRP was assessed using a water-soluble tetrazolium assay. Data are presented as the mean ± SEM. At each time point, TRP-treated and untreated fibroblasts were compared separately within the control and IPF groups using the Mann–Whitney U test. *p < 0.05 vs. the corresponding untreated group.

In the present study, we investigated the role of TRP in IPF-associated fibrogenesis. Lung tissue and fibroblasts from IPF patients were used to analyze the expression of enzymes that make up the TRP metabolic pathway. Cultures of primary lung fibroblasts were also employed in mechanistic experiments, and the effects of TRP in mice with bleomycin (BLM)-induced lung fibrosis, as an animal model of IPF, were evaluated.

## Materials and methods

### Reagents and antibodies

The specific primary antibodies used in this study were anti-collagen Ⅰ (Abcam Cat# ab34710, RRID:AB_731684), anti-fibronectin (Sigma-Aldrich Cat# F7387, RRID:AB_476988), anti-α-smooth muscle (Abcam Cat# ab7817, RRID:AB_262054), β-actin (Sigma-Aldrich Cat# A1978, RRID:AB_476692), anti-phospho-Akt (Santa Cruz Biotechnology Cat# sc-135650, RRID:AB_2224730), anti-phospho-mTOR (Cell Signaling Technology Cat# 2971, RRID:AB_330970), anti-phospho-4E-BP1 (Cell Signaling Technology Cat# 2855, RRID:AB_560835), anti-phospho-S6 ribosomal protein (Cell Signaling Technology Cat# 4858, RRID:AB_916156), and TPH-1 (Thermo Fisher Scientific Cat# PA1–777, RRID:AB_2256401). The secondary antibody was horseradish-peroxidase (HRP)-conjugated anti-rabbit or anti-mouse immunoglobulin G (IgG) (GenDEPOT Cat# SA001, RRID:AB_3676717), (GenDEPOT Cat# SA002–500, RRID:AB_3676718). TRP was purchased from Sigma Aldrich (#T0254–5G), WST-1 from Roche Diagnostics, RIPA lysis buffer (#WSE-7420) from ATTO (Tokyo, Japan), Masson’s trichrome staining kit (no. KTMTR) from American Mastertech (Lodi, CA, USA), and the hydroxyproline assay kit (MAK008) from Sigma-Aldrich. The TPH-1 (#MBS2023511) and IDO (#MBS2022556) ELISA kits as well as Dulbecco’s MEM (DMEM) F-12 w/o TRP (#MBS653056) were from MyBioSource (San Diego, CA, USA).

### Human samples

Samples of lung tissue from adult patients with IPF and from controls were obtained from the Biobank at Soonchunhyang University Bucheon Hospital (Bucheon-si, South Korea). All methods were performed in accordance with relevant guideline and regulation. All human lung tissue samples from patients with IPF (*n* *=* *6*) and control subjects (*n* *=* *6*) were obtained from the SCH Biobank (Soonchunhyang Biobank, Bucheon, Korea), and the samples were obtained from the Biobank on 27/09/2017. The protocol for the use of human samples was approved by the Institutional Review Board of Soonchunhyang University Bucheon Hospital (SCHBC 2016–12–024–002), and all procedures were performed in accordance with the Declaration of Helsinki. Written informed consent was obtained from all participants or their guardians at the time of sample deposition in the Soonchunhyang University Bucheon Hospital Biobank.

### Fibroblast isolation and culture

Briefly, the cells were derived from lung tissue obtained from IPF patients during video-assisted thoracoscopic biopsy, usually 1 week after hospital admission. None of the patients had been treated with corticosteroids or immunosuppressive drugs at the time of the biopsy. Control fibroblasts were obtained from patients who underwent a lobectomy to remove a primary lung tumor. No morphological evidence of disease was found in the tissue samples from which the control cells were isolated. Lung fibroblasts from IPF or control tissue were isolated by mechanical dispersal followed by trypsin digestion and further disruption to obtain ~1 mm^2^ fragments. Fibroblast cultures were established in DMEM supplemented with 10% FBS, 100 U penicillin/mL, 100 mg streptomycin/mL, and 0.25 μg amphotericin B/mL and incubated at 37°C in 95% air/5% CO2 until just prior to reaching confluence (1–2 weeks). After three passages, adherent cells harvested from the same culture vessels were analyzed by immunoblotting using anti-vimentin antibodies. All cells had the morphological characteristics of fibroblasts. Experiments with IPF and control fibroblasts were performed on cells before the sixth passage.

### Proliferation assays

Primary human lung fibroblasts and normal control cells were seeded in 96-well plates and incubated for 24 h before they were stimulated with 10 μM TRP (Sigma Aldrich) in medium containing 2% FBS. After 24 or 48 h incubation at 37°C they were tested using a WST-1 (Roche Diagnostics) assay. The plates were read using a scanning multi-well spectrophotometer.

### Immunoblotting

To determine the mechanism by which TRP affects fibrosis signaling pathways, primary human lung fibroblasts in DMEM containing 2% FBS (MyBioSource) were treated (or not treated) with 10 μM TRP (Sigma Aldrich) and incubated at 37°C for 24 h. Thereafter, total protein was extracted from the cells using lysis buffer (ATTO) containing proteinase and phosphatase inhibitor cocktails followed by centrifugation. Immunoblotting was performed as described previously. For each experiment, equal amounts of total protein were resolved on a 10% SDS-polyacrylamide gel via electrophoresis, transferred to a polyvinylidene difluoride membrane (Sigma Aldrich), and incubated overnight at 4°C with the following primary antibodies: anti-collagen Ⅰ, anti-fibronectin, anti-α-smooth muscle, β-actin, anti-phospho-, anti-phospho-mTOR, anti-phospho-4E-BP1, anti-phospho-S6 ribosomal protein, and TPH-1. After several washes with TBS containing Tween-20 (Sigma Aldrich), the membrane was incubated with HRP-linked anti-rabbit or anti-mouse IgG (1:3000), as the secondary antibody, for 1 h. Then it was washed again several times as above and the bands were detected using chemiluminescence detection (Thermo Fisher Scientific) and a BioRad ChemiDoc Touch Imaging System (RRID:SCR_021693)

### Enzyme-linked immunosorbent assays (ELISAs) for TPH-1 and IDO

TPH-1 and IDO protein concentrations in IPF fibroblasts were measured in cell lysates using an ELISA kit (MyBioSource), in accordance with the manufacturer’s instructions. The lower limit of detection was 0.054 ng/mL for TPH-1 and 0.06 ng/mL for IDO. Values below these limits were recorded as 0 ng/mL. The inter- and intra-assay coefficients of variance were < 12%.

### Histological assays

A portion of each left lung was fixed in 4% (v/v) buffered paraformaldehyde, embedded in paraffin, and cut into sections 4 µm thick for H&E (histopathological analyses) or Masson’s trichrome (collagen content and distribution) staining. The right lung was snap-frozen by immersion in liquid nitrogen and stored at –80°C prior to RNA and protein extraction. The Ashcroft score was used to evaluate lung fibrosis as previously described [17].

### Immunohistochemical staining

Lung tissues were dehydrated, embedded in paraffin, and cut to yield sections 4 µm thick. The sections were treated with 1.4% H_2_O_2_-methanol for 30 min to block endogenous peroxidase, followed by incubation in 1.5% TBS to block nonspecific binding. Then they were incubated with anti-TPH-1 (1:100; Invitrogen) overnight at 4°C, washed several times with TBS, and then incubated for 1 h in TBS containing 1% FBS and biotinylated goat anti-rabbit lgG (Vector Laboratories, Burlingame, CA, USA). After several washes with TBS, the sections were incubated for 30 min with ABC kit reagents (Vector Laboratories, Burlingame, CA, USA). The color reaction was developed by staining with a liquid DAB  +  substrate kit (Golden Bridge International Inc., Mukilteo, WA, USA) and then counterstained for 1 min with Harris’s hematoxylin.

### Animal model of BLM-induced lung fibrosis and treatment with L-tryptophan

Male C57BL/6 mice (Orient Bio, Inc., Charles River, Sungnam, South Korea, RRID:MGI:2159769) were maintained under pathogen-free conditions with a 12-h light period at 21−23 °C and 40−50% humidity. Food and water were provided ad libitum. The experiments were conducted using 8-week-old male mice, were randomly assigned into four groups (n = 8 per group). On day 0, the mice were administered 3 mg BLM (Nippon, To det Kayaku, Takasaki-shi, Japan)/kg, dissolved in 100 µL endotoxin-free water, via intratracheal instillation. The appropriate dose of TRP was determined in a pilot study in which bleomycin-induced mice were treated with 20 mg TRP/kg in saline from day 0 to day 10, after which the inflammatory cells were analyzed. Sham control mice were treated with PBS only. On day 21, the mice were anesthetized using Zoletil (80 mg/kg) and Rompun (5 mg/kg) to induce general anesthesia in the animals, and the mice were euthanized by cutting the inferior vena cava. And bronchoalveolar lavage fluid (BALF) was obtained by instilling 1 mL PBS, which was gently retrieved, four times as described previously [17]. Cell numbers were measured using a hemocytometer, and differential cell counts were performed on slides prepared by cytocentrifugation and Diff-Quik staining (E. Merck KG, Darmstadt, Germany). BALF was centrifuged at 1000 rpm for 15 min, and the supernatant was stored at –80°C. Portions of the cell-free supernatants were used for biochemical analyses. Animals were euthanzied if predefined clinical signs were observed during the experiment, or if infection or other conditions resulted in a body weight loss of 20% or more compared with age-matched healthy animals. Once animals reached these endpoint criteria, euthansia was performed immediately. Aminal health and behavior were monitored twice daily throughout the study. No animals died before meeting the criteria for euthansia. All animal procedures followed a protocol approved by the Institutional Animal Care and Use Committee (IACUC) of Soonchunhyang University Bucheon Hospital (SCHBC_Animal_2016-12), and all personnel involved in the animal experiments received appropriate training in animal care and handling, including animal ethics and welfare education provided by the IACUC. All methods are reported in accordance with ARRIVE guidelines for the reporting of animal experiments.

### Masson’s trichrome staining

A Masson’s trichrome stain kit (StatLap) was used as follows. Mouse lung sections were placed in Bouin’s solution at 56°C for 1 h and then stained successively with Mayer’s hematoxylin solution for 5 min, Biebrich scarlet-acid fuchsin solution for 5 min, phosphomolybdic acid-phosphotungstic acid for 15 min, and aniline blue for 1 h. The sections were examined under a microscope.

### Measurement of hydroxyproline in the lungs of mice

The amount of collagen in the lungs was quantified via hydroxyproline assays (Sigma-Aldrich), performed using tissue from the right lung of each mouse, in accordance with the manufacturer’s instructions. Briefly, the lungs were weighed, homogenized in sterile water, and hydrolyzed in 12 N HCl at 120°C for 3 h. The hydrolyzed samples were incubated with 4-(dimethylamino) benzaldehyde for 90 min at 60°C, after which the absorbance of oxidized hydroxyproline was determined at 560 nm. The results are expressed as µg/mg lung tissue.

### Statistical analyses

The data were analyzed using a Kruskal–Wallis’s test, followed by a Mann–Whitney U test (with Bonferroni correction for intergroup comparisons). All data are expressed as the mean  ±  standard error; p values  <  0.05 were considered to indicate statistical significance.

## Results

### Treatment with TRP promotes the proliferation of IPF fibroblasts

TRP levels were significantly elevated in lung tissues from patients with IPF compared with control subjects (FDR-adjusted p = 0.0099) [16] (Fig 1a). Based on this finding, we next investigated whether increased TRP levels could influence lung fibroblast proliferation. To identify the TRP concentration conducive to optimal cell growth, the cells were treated with TRP at concentrations of 1, 10, and 100 µM, and proliferation was compared. In normal lung fibroblasts, an increase in the TRP concentration from 1- to 16-fold resulted in a corresponding increase in the cell count, with 10 µM TRP inducing the highest level of proliferative activity (Supplementary S1 Fig). In IPF fibroblasts, proliferation at 24 and 48 h was significantly higher in cultures treated with 10 µM TRP than in controls (24 h, p = 0.0005; 48 h, p = 0.0035) (Fig 1b). These results demonstrate the ability of TRP to significantly increase the proliferation of IPF fibroblasts and thus its potential role in the pathogenesis of IPF.

### Tryptophan regulates the production of collagen, fibronectin, and α-SMA in IPF fibroblasts

Given the increased proliferation of IPF fibroblasts in response to TRP, its ability to regulate the main ECM components in lung fibroblasts was investigated. Primary fibroblasts from both IPF (*n = 4*) and control (*n = 4*) cultures were treated with 10 µM TRP, followed by Western blotting analysis of collagen I, fibronectin, and α-smooth muscle actin (SMA). The expression of all three proteins increased in IPF fibroblasts treated with TRP (all p < 0.05), but the changes were not significant in non-treated control fibroblasts (Fig 2a). Then the impact of TRP depletion on ECM components was assessed by culturing IPF fibroblasts for 48 h in TRP-free and TRP-containing media. Western blotting analysis revealed significantly lower levels of collagen I, fibronectin, and α-SMA in fibroblasts cultured in TRP-free medium than in TRP-containing medium (all p < 0.05) (Fig 2b). These results suggest that TRP regulates the production of ECM components in IPF fibroblasts.

**Fig 2:**
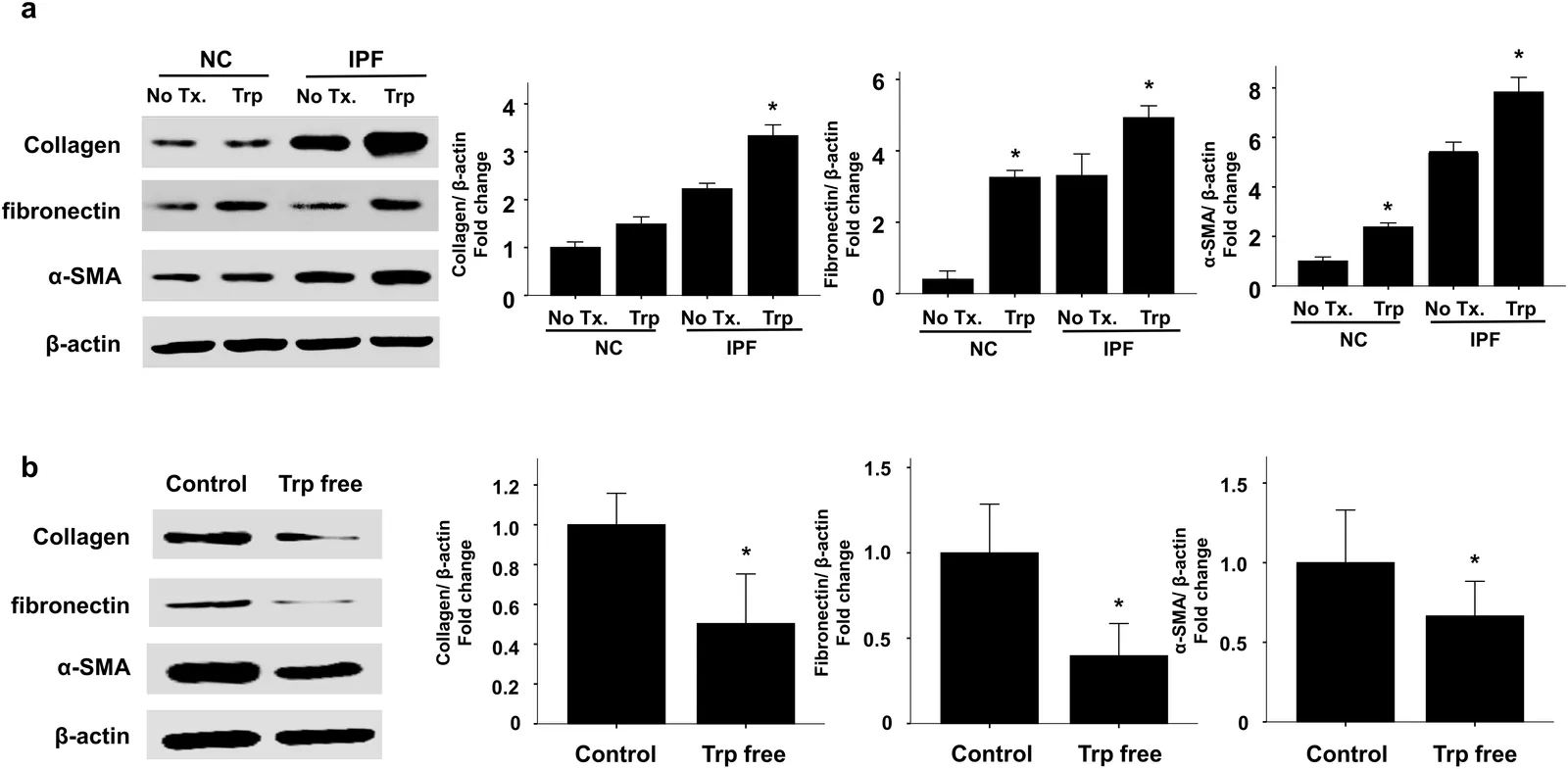
L-TRP regulates the expression of extracellular matrix (ECM) components in IPF fibroblasts. The effects of TRP on the expression of collagen, fibronectin, and α-smooth muscle actin (SMA) in control and IPF fibroblasts. **a**. Control and IPF fibroblasts were treated with or without 10 µM TRP for 24 h. Protein levels of collagen I, fibronectin, and α-SMA were quantified by immunoblotting and densitometry (*n = 4* per group). **b**. IPF fibroblasts were cultured in TRP-containing or TRP-free medium for 24 h. Protein levels were quantified by immunoblotting and densitometry (*n = 4* per group). Data are presented as the mean ± SEM. Statistical analyses were performed using the Kruskal–Wallis test followed by the Mann–Whitney U test with Bonferroni correction. *p < 0.05 versus the corresponding untreated group within each fibroblast group.

### Tryptophan upregulates AKT-mTOR signaling pathway proteins

The mechanism underlying the ability of TRP to regulate fibroblast proliferation was explored by examining the PI3K/AKT/mTOR pathway, which mediates a wide range of cellular processes including cell survival, growth, and proliferation [18–20]. Specifically, the expression of Akt and mTOR together with that of the downstream signaling molecules 4EBP and p70S6K was examined by Western blotting in IPF fibroblasts treated with 10 µM TRP. The increased phosphorylation of all four proteins in TRP-treated fibroblasts (all p < 0.05) (Fig 3a) suggested a role for the TRP-mediated upregulation of the AKT-mTOR signaling pathway in stimulating fibroblast proliferation.

**Fig 3:**
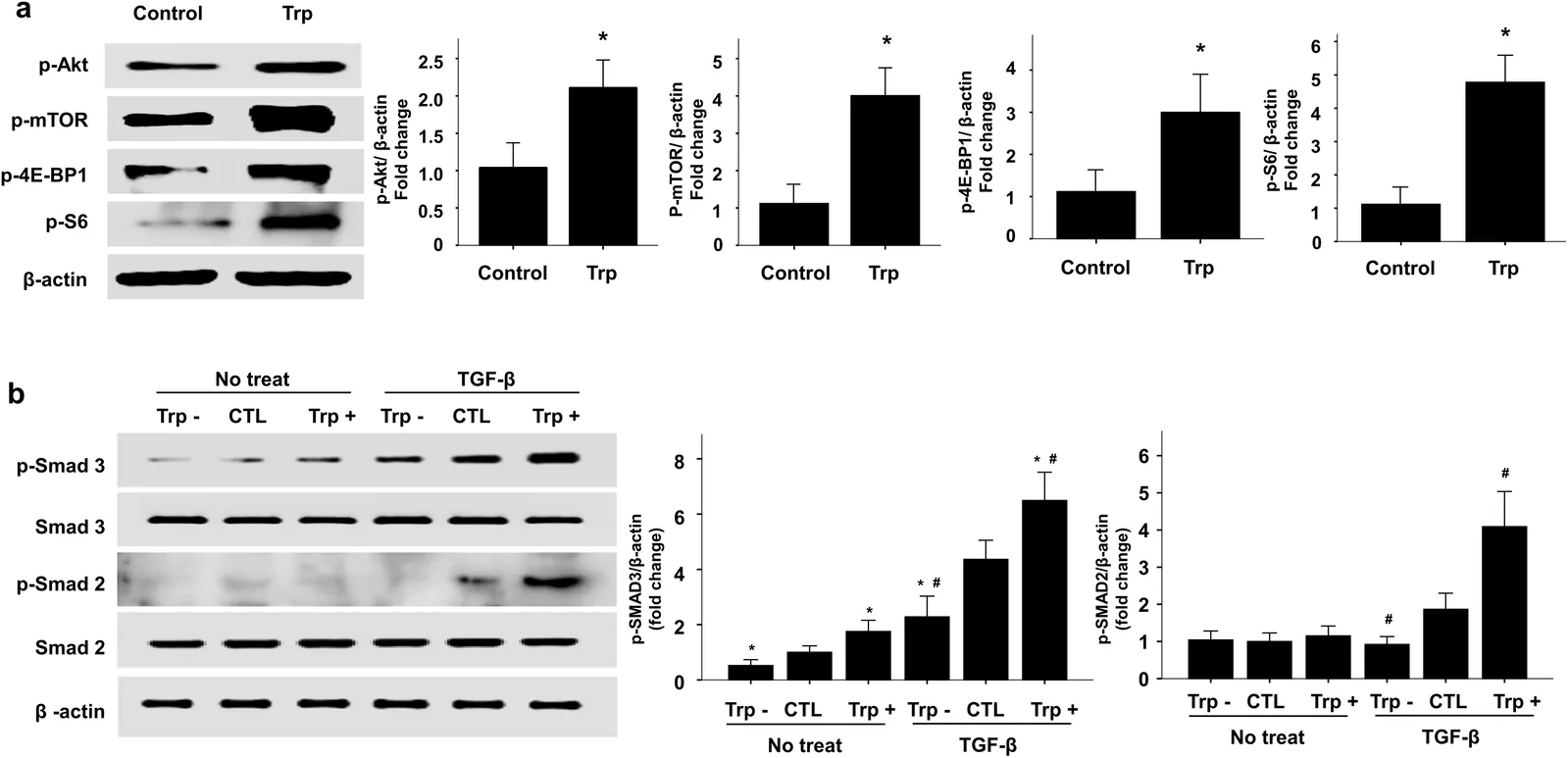
TRP regulates AKT-mTOR signaling and the SMAD pathway in IPF fibroblasts. **a.** IPF fibroblasts were treated with or without 10 µM TRP for 24 h. The levels of p-AKT, p-mTOR, p-4E-BP1, and p-S6 were quantified by immunoblotting and densitometry (n = 4 per group). **b.** IPF fibroblasts were cultured under the indicated TRP conditions with or without TGF-β. The levels of p-SMAD2 and p-SMAD3 were quantified by immunoblotting and densitometry (*n = 4* per group). Data are presented as the mean ± SEM. Statistical analyses were performed using the Kruskal–Wallis test followed by the Mann–Whitney U test with Bonferroni correction for intergroup comparisons. *p < 0.05 vs. the corresponding untreated control; #p < 0.05 vs. the corresponding TGF-β-treated group.

### Effect of tryptophan on the TGF-β signaling pathway

TGF-β plays a crucial role in lung fibrosis [21,22]. Its binding to its transmembrane receptor initiates a signal transduction process that leads to the phosphorylation of Smad2/3. The resultant heteromeric p-Smad complex then upregulates the transcription of fibrosis-promoting genes such as a-SMA, collagen, and fibronectin [21,23]. In this study, the involvement of Smad molecules in the effects of TRP on IPF fibroblasts was examined by culturing the cells under three conditions: TRP depletion, control, and TRP treatment. Then the cells in each group were treated with TGF-β and the expression of Smad molecules was determined. As shown in Fig 3b, while TRP induced an increase in p-Smad3 levels, in the absence of TGF-β there was no increase in p-Smad2 expression in any of the fibroblast groups regardless of TRP treatment. However, among the fibroblasts treated with TGF-β and TRP, the levels of both p-Smad2 and p-Smad3 increased (all p < 0.05), whereas in the TRP-depleted cultures, p-Smad2 expression in response to TGF-β was negligible. These findings suggest that the TGF-β-activated signaling pathway in IPF fibroblasts is upregulated by the presence of TRP and downregulated by its absence.

### Tryptophan hydroxylase-1 is upregulated in the IPF lung and in IPF fibroblasts

The metabolism of TRP via two distinct pathways yields the metabolites 5-HT and kynurenine, respectively, which differ in their effects on fibroblast proliferation [12,24]. 5-HT is taken up by platelets, circulates via the blood, and is released upon tissue damage, causing fibroblast activation and initiating a wound-healing process that includes collagen deposition and tissue fibrosis [25]. The metabolism of TRP to 5-HT is the rate-limiting step in a reaction catalyzed by tryptophan hydroxylases (TPH-1, 2) [26,27]. In mice with BLM-induced lung fibrosis, 5-HT levels are increased whereas a 5-HT antagonist downregulates TGF-β levels [28]. These observations suggest that 5-HT has profibrotic effects in experimental lung fibrosis. The breakdown of TRP to yield N-formyl kynurenine, the precursor to L-kynurenine, is catalyzed by indoleamine 2,3-dioxygenase (IDO) [29]. The anti-fibrogenic activities of kynurenine suggest its utility in treating hypertrophic scarring [13], while studies in rats with lung allografts have shown that IDO overexpression hinders the proliferation of lung fibroblasts [30]. Feedback inhibition between these two TRP metabolic pathways has been investigated in skin fibroblasts [31] but not in IPF. We hypothesized that TRP increases profibrotic activity by activating the 5-HT pathway in the IPF lung. In ELISA analyses, TPH-1 levels in IPF fibroblasts were significantly higher than in controls (p < 0.0001), whereas IDO levels did not differ (p = 0.851) (Fig 4a). Immunohistochemical staining showed that, in IPF lung tissue, TPH-1 was predominantly expressed in alveolar macrophages as well as in perivascular and fibrotic areas (Fig 4b). The levels of TPH-1 were significantly elevated in human IPF samples and in the lungs of BLM-treated mice. These findings suggest that the profibrotic activities of TRP contribute to the pathogenesis of IPF and can be explained, at least partly, by the increased levels of TPH-1 in the lung.

**Fig 4:**
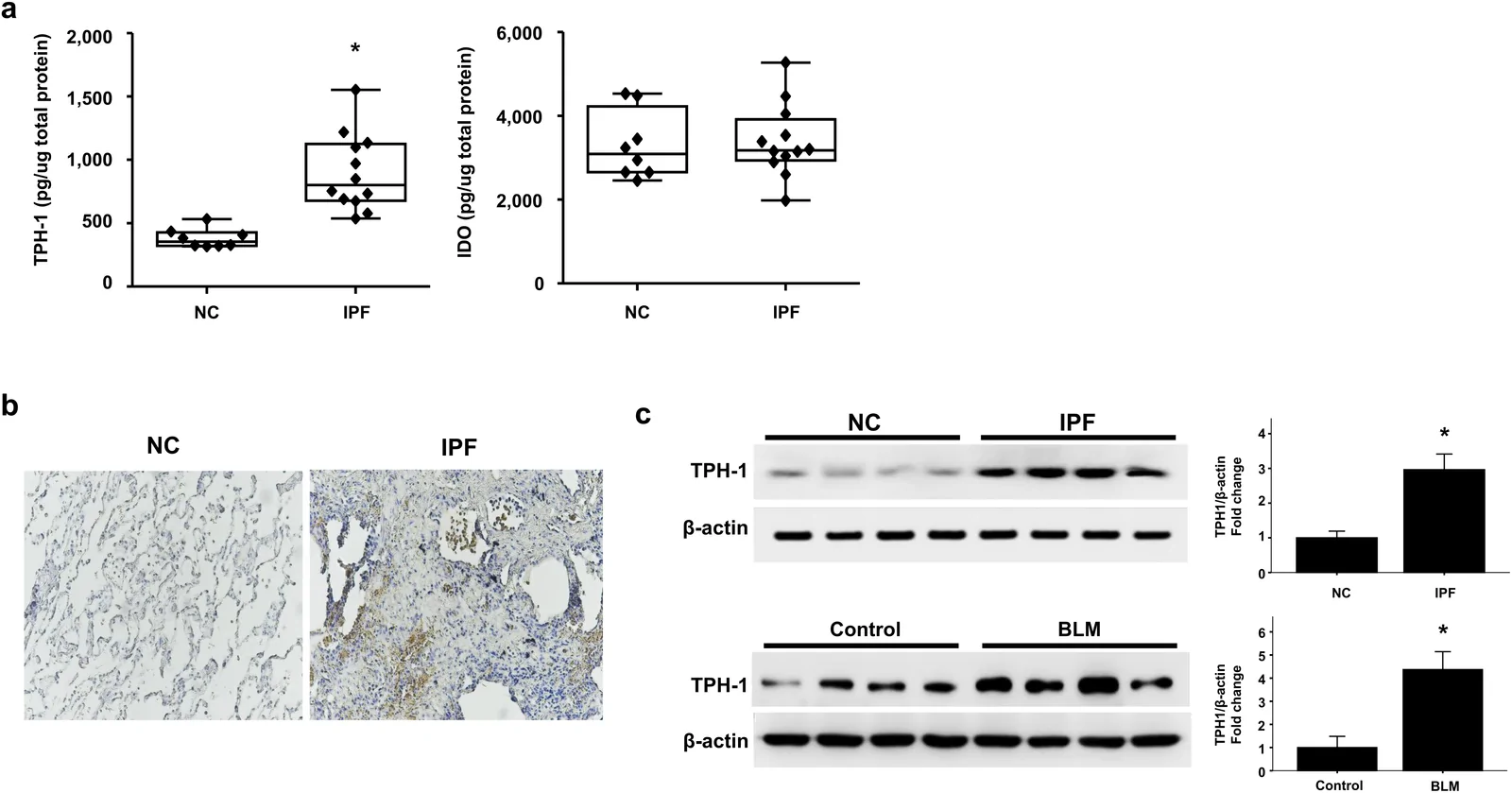
TPH-1 levels are increased in human IPF samples and in the lungs of BLM-treated mice. a. TPH-1 and IDO levels in primary lung fibroblasts from control subjects *(n = 8)* and patients with IPF *(n = 12)* were quantified by ELISA. Data are presented as the median (interquartile range [IQR]) and were analyzed using the Mann–Whitney U test. b. Immunohistochemical staining for TPH-1 was performed in lung tissues from control subjects and patients with IPF; original magnification, 100 ×. c. TPH-1 expression was examined by immunoblotting in primary human lung fibroblasts from control subjects and patients with IPF and in lung tissues from control and BLM-treated mice (*n = 4* each), followed by densitometric quantification. Data are presented as the mean ± SEM. Statistical comparisons were performed using the Mann–Whitney U test. *p < 0.05 vs. the corresponding control group.

### Effect of TRP administration on BLM-induced lung injury/fibrosis

Whether exogenous TRP treatment aggravates fibrosis was examined in mice with BLM-induced lung fibrosis. The mice were intraperitoneally injected daily from day 0 to day 10 with TRP (20 mg/kg in mice) and lung samples were obtained on day 21. Histochemical staining with hematoxylin and eosin (H&E) showed that exogenous TRP treatment markedly aggravated the structural distortion in the fibrotic lungs of BLM-treated mice (Fig 5a). Consistent with the histology results, a BALF analysis showed that TRP significantly increased total cell counts, as well as the numbers of macrophages, neutrophils, and lymphocytes (total cells, p = 0.015; macrophages, p = 0.030; neutrophils, p = 0.006; lymphocytes, p = 0.028), indicating upregulated lung inflammation (Fig 5b). Masson’s trichrome staining and Ashcroft scoring showed that TRP considerably increased collagen deposition and thus lung fibrosis (Ashcroft score, p = 0.0002) (Figs 5c and 5d). A hydroxyproline assay demonstrated the profibrotic activity of TRP treatment in the BLM mice (p = 0.016) (Fig 5e). While the level of the active form of TGF-β1 was significantly increased in the lungs of BLM-exposed mice, it was increased even further by TRP treatment (p = 0.037) (Fig 5f). The ability of TRP to aggravate experimental lung fibrosis is consistent with its detrimental effect in fibrotic lung disease.

**Fig 5:**
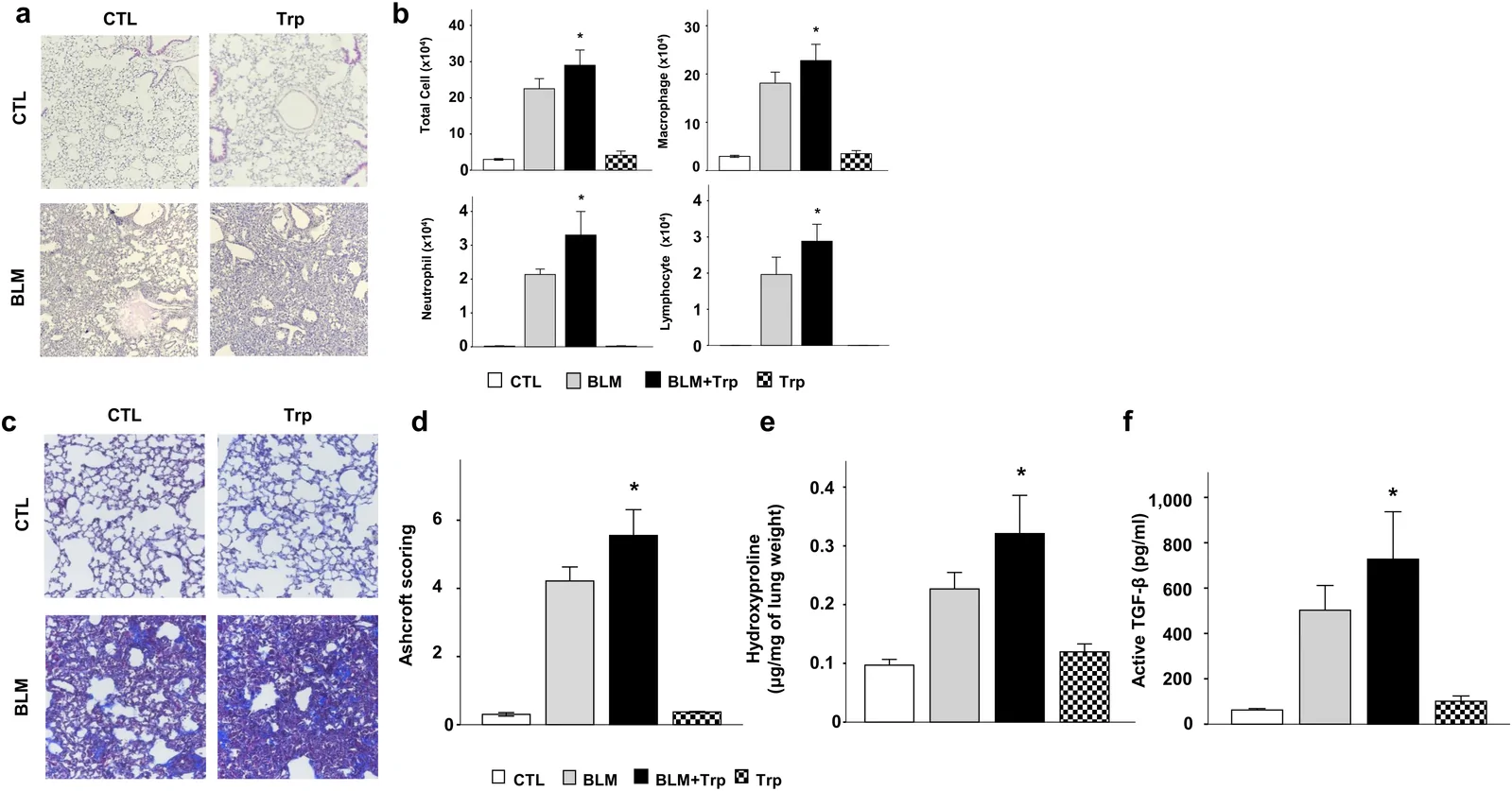
L-TRP exacerbates lung inflammation and fibrosis in BLM-treated mice. **a.** Mice were intranasally administered TRP (20 mg/kg) on days 0–12; lung tissues were collected on day 21 and stained with H&E; magnification, 100 ×**. b.** Cell counts in BALF collected on day 21; differences between BALF cell counts were analyzed based on 500 cells stained with Diff-Quik (*n = 8* per group). **c.** Masson’s trichrome staining; magnification, 100 ×. d. Lung fibrosis was quantified using the Ashcroft score (*n = 8* per group). **e.** Collagen expression in the lungs was measured using hydroxyproline assays performed in control and BLM-treated mice treated or not with TRP (*n = 8* per group)**. f.** Active TGF-β1 levels in the lungs of control and BLM-treated mice treated or not with TRP (*n = 8* per group) were quantified in lung lysates by ELISA. Data are presented as the mean ± SEM. Statistical comparisons between the BLM and BLM + TRP groups were performed using the Mann–Whitney U test. *p < 0.05 vs. the BLM-treated group.

## Discussion

Consistent with our previous metabolomic study showing elevated TRP levels in the lungs of IPF patients, in the present study, elevated levels of TPH1, the key enzyme responsible for converting TRP to serotonin, in IPF lungs were identified. In addition, exogenous TRP was found to aggravate BLM-induced lung inflammation and fibrosis in mice, implicating increased TRP levels in IPF progression. An exploration of the mechanism underlying the effects of TRP revealed the activation of the AKT-mTORC1 and TGF-β pathways in primary cultures of IPF fibroblasts, the proliferative cells responsible for the increased production of ECM components in IPF. Taken together, our results suggest that increased levels of TPH-1 and thus of TRP in the lung contribute to the fibrogenesis associated with IPF.

TRP is an essential amino acid that plays a crucial role in many metabolic functions. Its metabolites include kynurenine and 5-HT [32]. While several studies have reported that 5-HT activation during the earliest phases of wound repair strongly influences fibrogenesis [33,34], few studies have examined the relationship between TRP and tissue fibrosis, and as far as we know, none have investigated the role of TRP in IPF. In our study, exogenous TRP significantly enhanced the proliferation of IPF fibroblasts and upregulated the expression of the main ECM components, collagen I, fibronectin, and α-SMA (Fig 2a). By contrast, TRP deprivation resulted in a significant reduction of collagen I and fibronectin in IPF fibroblasts (Fig 2b). This effect was mediated by the inhibition of TGF-β1 activities. Targeting TRP metabolism may therefore offer a therapeutic strategy in patients with fibrotic lung disease.

Recent research has demonstrated the critical roles of 5-HT and kynurenines, the metabolic byproducts of TRP, in fibrotic disease [35–37]. In the 5-HT pathway, TPH-1 catalyzes the hydroxylation of TRP to 5-hydroxytryptophan, which then is decarboxylated to 5-HT, an activator of fibroblast proliferation and collagen deposition. In the kynurenine pathway, IDO converts TRP into kynurenine, which has been reported to exert antifibrotic effects by suppressing fibroblast activation [12,13]. These contrasting effects of serotonin and kynurenine suggest that the biological consequences of increased TRP availability may depend, at least in part, on its metabolism through these distinct pathways [12,13]. In the present study, TPH-1 expression was increased whereas IDO levels were unchanged, suggesting differential regulation of the serotonin and kynurenine pathways in IPF fibroblasts. However, because serotonin, kynurenine, and other downstream metabolites were not directly measured, the relative contribution of these pathways remains to be determined. The association between elevated TPH-1 levels and the profibrotic activities of TRP is supported by a study in which TPH-1 knockout significantly reduced BLM-induced pulmonary fibrosis in mice [38].

We found significantly increased TPH-1 levels both in IPF lung tissues and in cultured primary IPF fibroblasts (Figs 4a-c), such that TPH-1 may provide a therapeutic target in human lung fibrosis. Furthermore, the ability of 5-HT to promote fibrogenesis suggests that activation of the 5-HT pathway contributes to TRP-induced fibrosis. However, we did not examine whether lung levels of 5-HT are elevated in IPF lungs, nor the potential involvement of other pathways, including the kynurenine pathway.

TRP significantly increased fibroblast proliferation *in vitro*, with stronger effects in IPF than in control fibroblasts (Fig 1b). The PI3K/AKT/mTOR pathway regulates cell growth, proliferation, and survival [39] and its deregulation has been implicated in a vast array of disease, including solid tumors, immune-mediated diseases, and IPF [40]. Indeed, several drugs targeting the PI3K signaling pathway are used to treat IPF, and experimental trials of similarly acting drugs for the treatment of other diseases have achieved promising results [39]. For example, in one study, while TRP treatment increased hepatic steatosis via mTOR phosphorylation, the mTOR inhibitor rapamycin reversed this effect, suggesting that mTOR activation is essential in the TRP-mediated exacerbation of hepatic steatosis [41]. In another study, 5-HT was found to activate Src/PI3K/AKT/mTOR/p70S6K phosphorylation signaling via G-protein coupled receptors, as also demonstrated for vascular endothelial growth factor [42]. In our study, we likewise found that TRP increased the phosphorylation of AKT, mTOR, and downstream signaling molecules, including 4E-BP1 and S6, in IPF fibroblasts (Fig 3a), supporting an association between TRP treatment and AKT-mTORC1 pathway activation. However, pharmacological or genetic inhibition of this pathway was not performed; therefore, its causal involvement in TRP-induced fibroblast activation remains to be established.

TGF-β is a fibrogenic cytokine able to induce the differentiation of fibroblasts into myofibroblasts. TGF-β signaling proceeds through the serine/threonine kinase of its transmembrane receptor, resulting in the activation of cytoplasmic Smad proteins [43]. The TRP metabolite 5-MTP attenuates pulmonary fibrosis by inhibiting the phosphorylation of TGF-β/SMAD3, PI3K/AKT pathway components [14]. However, whether the profibrotic activities of TRP are associated with modulation of TGF-β activity is unclear. We found increased expression of Smad2 and Smad3 in the presence of TRP, i.e., that TRP enhances TGF-β signaling in IPF fibroblasts (Fig 3b). According to this finding, TRP not only promotes fibroblast proliferation but also amplifies the fibrotic response via enhanced TGF-β signaling. Modulating TRP levels or downstream TRP-related signaling pathways may thus be effective for downregulating the expression of profibrosis genes in IPF patents.

Finally, the results of our *in vivo* analyses of BLM-induced lung fibrosis in mice were consistent with those of our *in vitro* an analyses. Specifically, TRP administration not only increased inflammation and structural distortion of the lung but also aggravated fibrosis caused by collagen deposition (Figs 5a, 5c, and 5d). In BLM mice, the active form of TGF-β1 was significantly increased following TRP treatment, thus supporting the profibrotic role of TRP *in vivo* (Fig 5f).

Despite highlighting a role for TRP in IPF, our study had several limitations. First, lung tissues and fibroblasts were obtained from a relatively small number of IPF patients, such that the variability and heterogeneity observed in the broader IPF population may not have been adequately reflected. Therefore, validation in larger independent cohorts is warranted. In addition, although TRP increased AKT-mTORC1 signaling, pharmacological or genetic inhibition of this pathway was not performed. Further studies using pathway-specific inhibition are required to establish its causal role in the profibrotic effects of TRP. Second, although TPH-1 expression was increased in IPF lung tissues and fibroblasts, serotonin (5-HT) levels were not directly measured. Moreover, although IDO expression was assessed, kynurenine and other downstream metabolites were not quantified. Therefore, the present study cannot determine the relative contributions of TRP itself, the serotonin pathway, and the kynurenine pathway to the observed profibrotic effects. Indeed, several studies have reported the antifibrotic effects of the TRP metabolite 5-MTP. Further studies incorporating direct measurements of TRP metabolites and pathway-specific interventions are required to clarify the respective contributions of these metabolic pathways. Nonetheless, our study is the first to identify the involvement of TRP in fibrogenesis, both in IPF lungs and in primary IPF fibroblasts. TRP was shown to upregulate known profibrotic pathways, and specifically AKT-mTORC1 and TGF-β, in IPF fibroblasts. These findings suggest the modulation of TRP metabolism as a therapeutic strategy in IPF patients.

## Conclusions

Our findings support a pathogenic role for TRP in IPF by promoting profibrotic fibroblast responses and worsening pulmonary fibrosis in vivo. These results further suggest that altered TRP metabolism may represent a potential therapeutic target in pulmonary fibrosis.

## Supporting information

S1 Fig L-tryptophan increases proliferation of IPF fibroblasts in a dose- and time-dependent manner. (DOCX)

S2 File Original uncropped and unadjusted immunoblot images. (PDF)
