# Supplementary material for: Exogenous tryptophan aggravates experimental lung fibrosis through activation of AKT-mTORC1 pathway
Source: PLoS One. 2026 Nov 3;21(11):e0361463. doi: 10.1371/journal.pone.0361463 (PMC13730385; doi:10.1371/journal.pone.0361463)

**Fig. 2a. Fibronectin. Original uncropped chemiluminescence images.**

The loading order and identity of experimental samples are indicated above each image. The molecular weight marker lane (M) is indicated where applicable. The expected molecular weight of fibronectin is approximately 260 kDa. All images were captured using a ChemiDoc™ Touch Imaging System (Bio-Rad Laboratories, Hercules, CA, USA).

**Replicate 1**

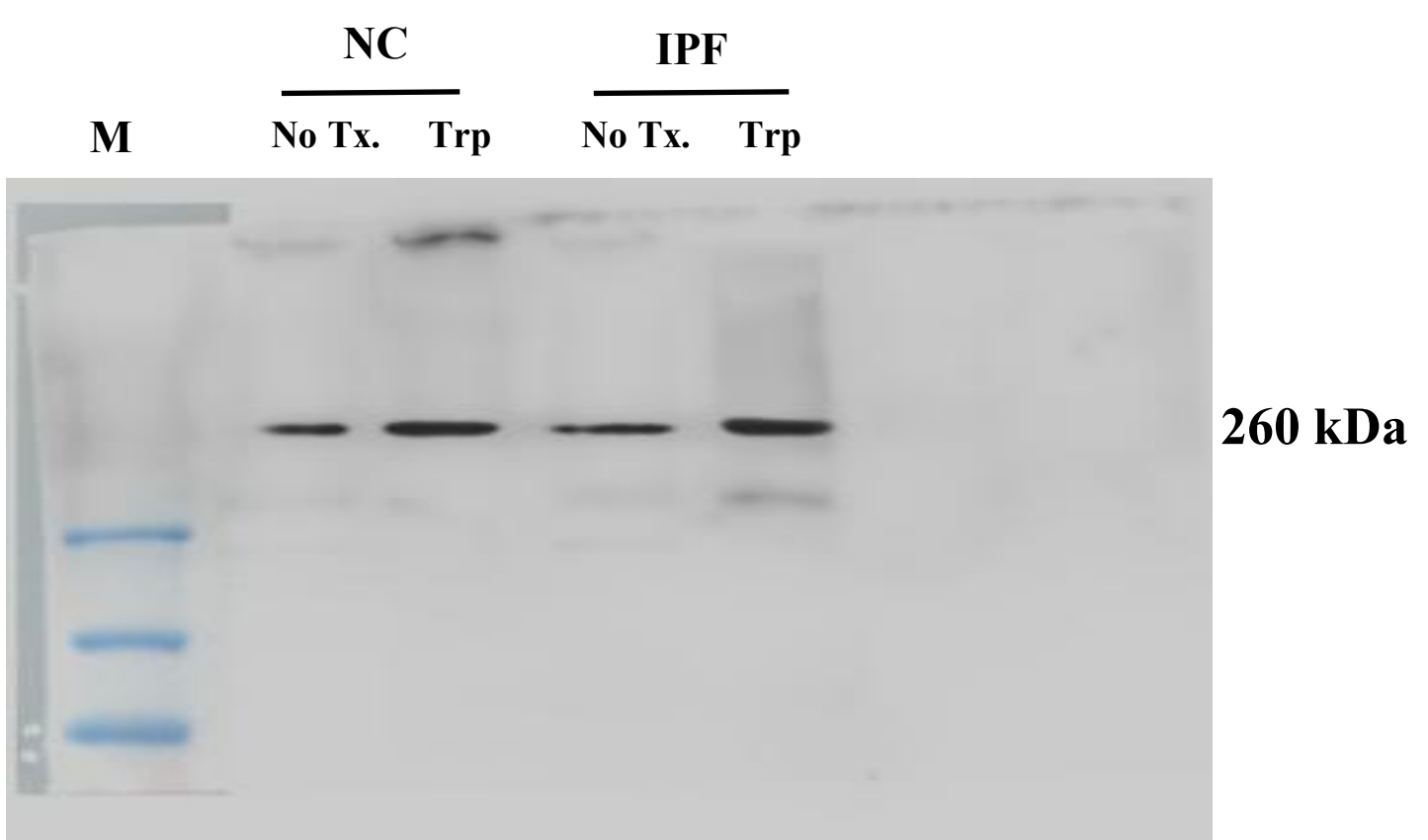


**Replicate 2**

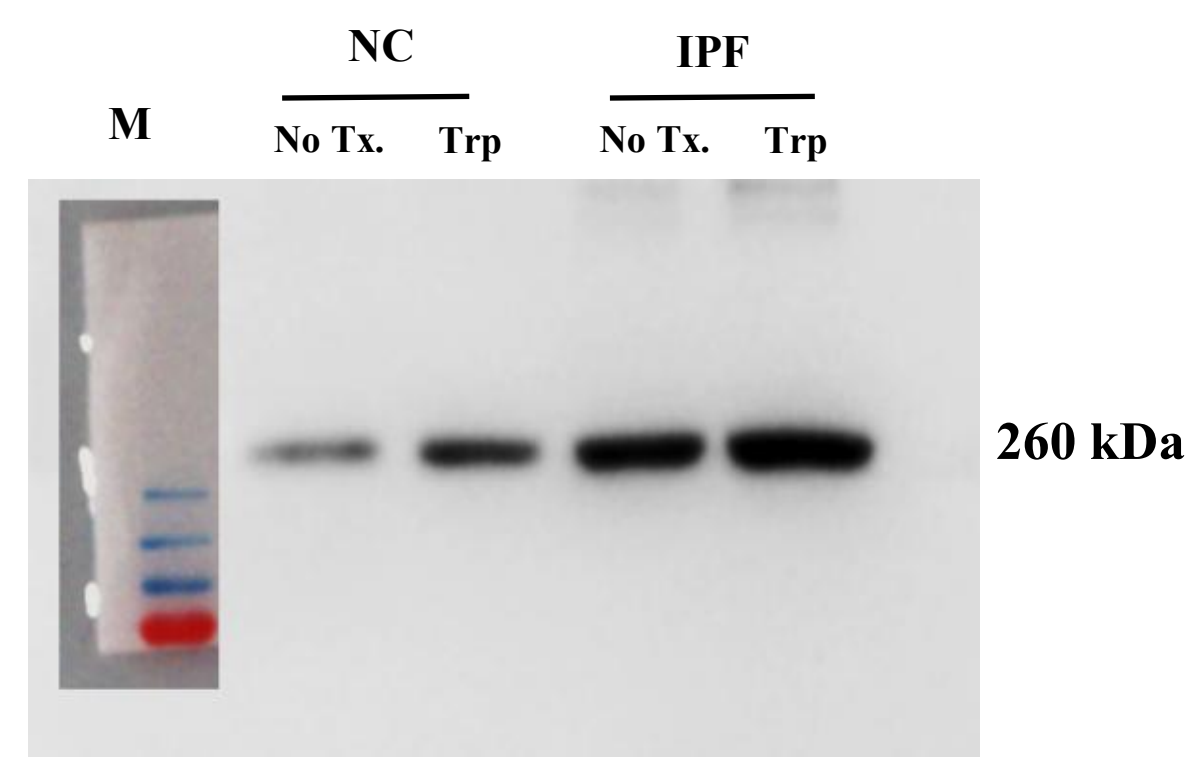


**Replicate 3**

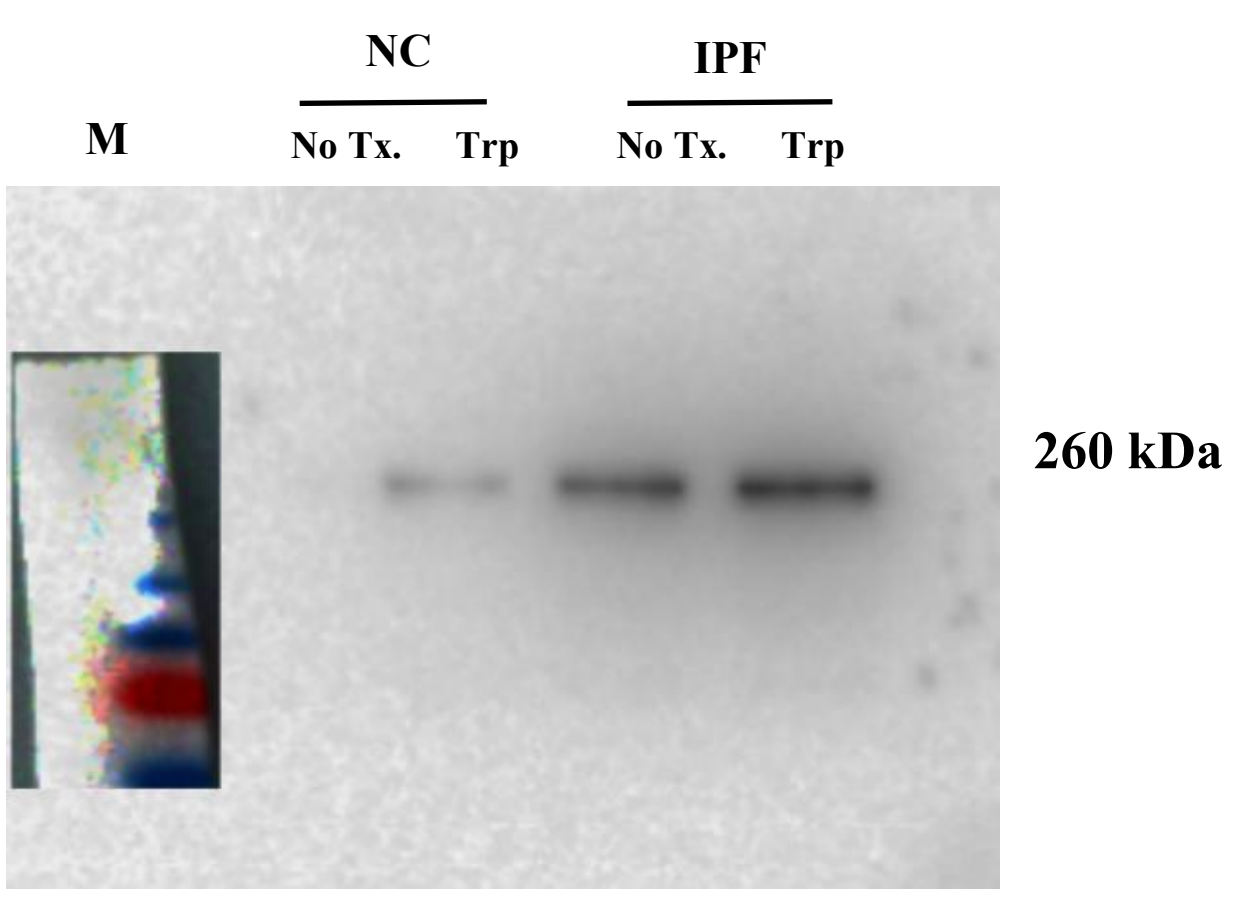


**Replicate 4**

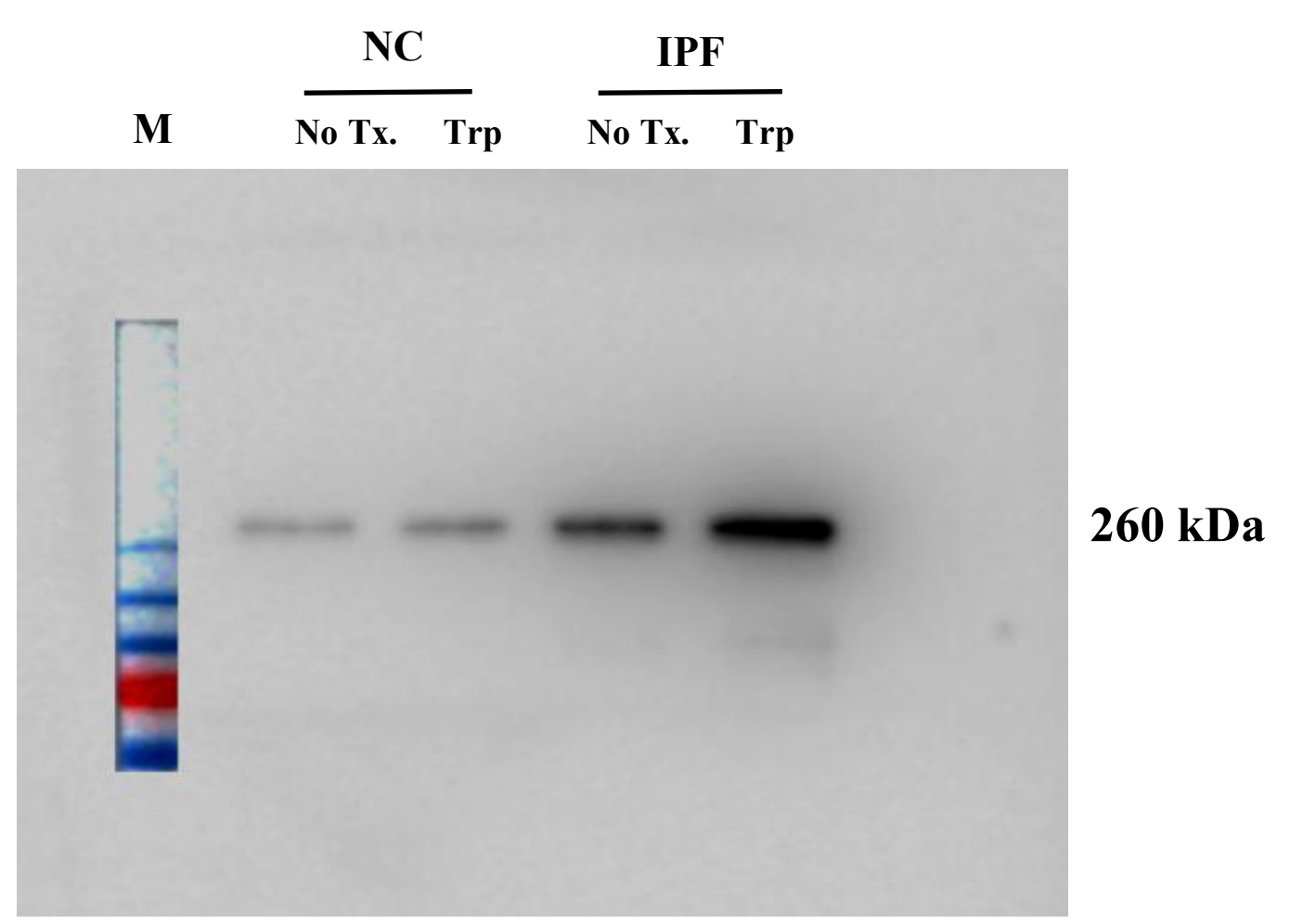

**Fig. 2a. Collagen. Original uncropped chemiluminescence images.**

The loading order and identity of experimental samples are indicated above each image. The molecular weight marker lane (M) is indicated where applicable. The expected molecular weight of fibronectin is approximately 130 kDa. All images were captured using a ChemiDoc™ Touch Imaging System (Bio-Rad Laboratories, Hercules, CA, USA).

**Replicate 1**

NC IPF

M No Tx. Trp No Tx. Trp

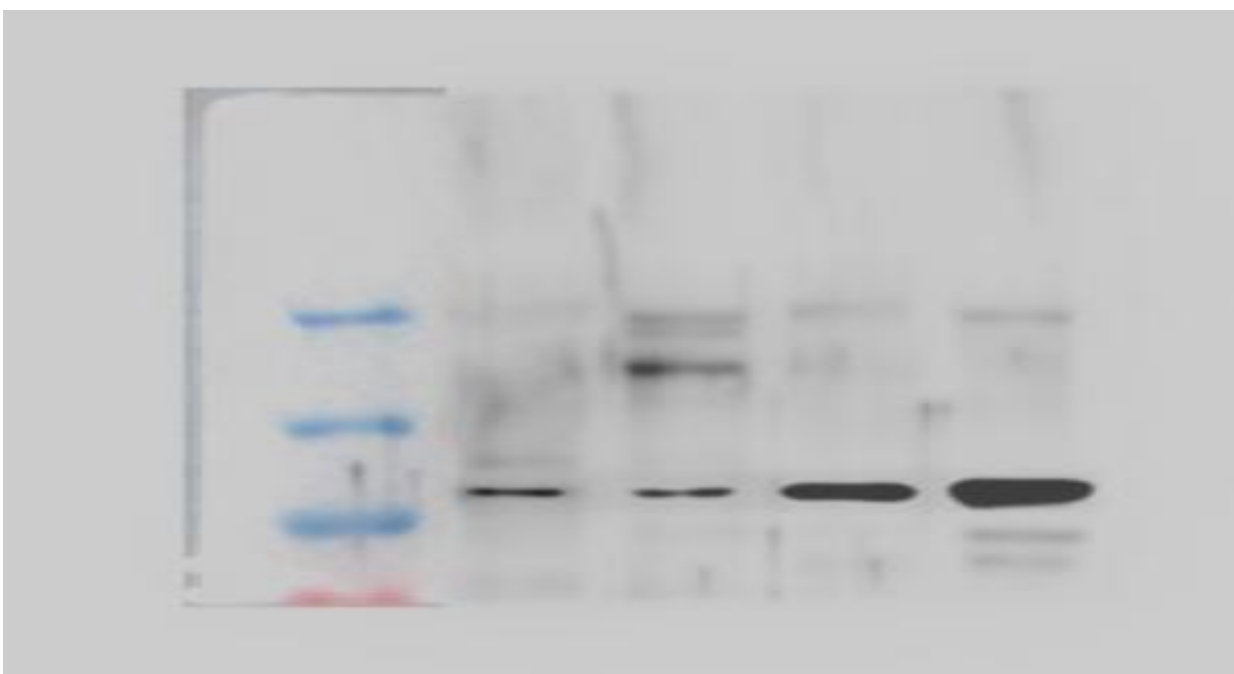

130 kDa

**Replicate 2**

NC IPF

M No Tx. Trp No Tx. Trp

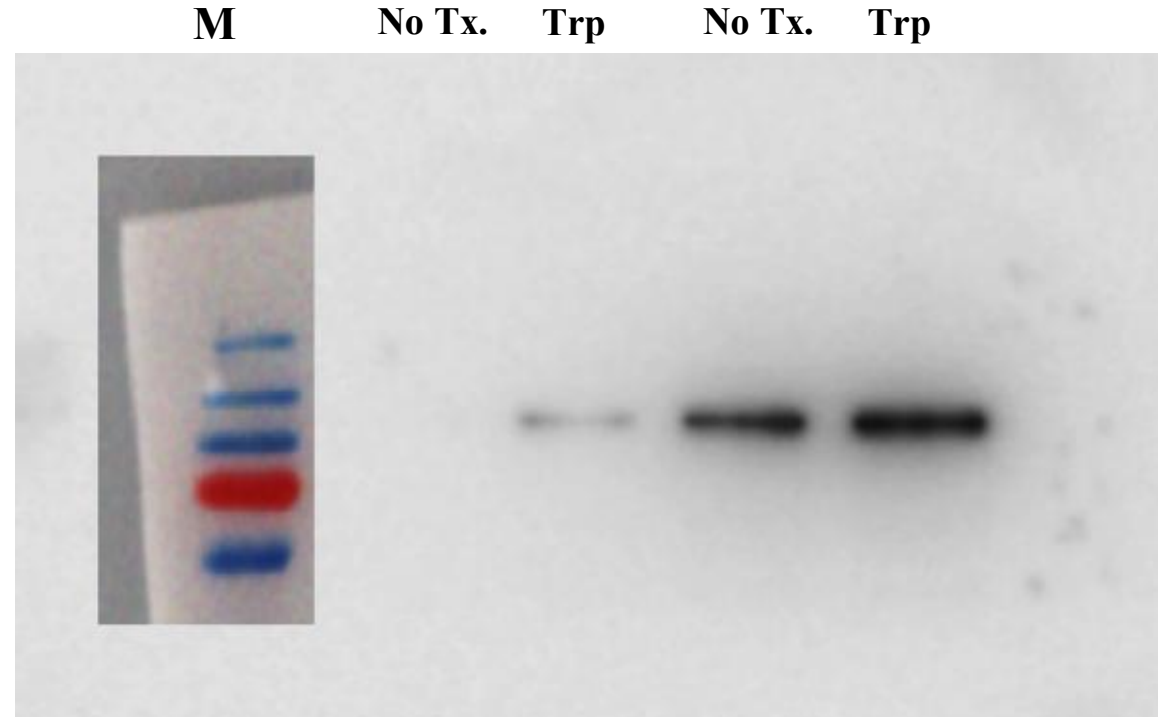

130 kDa

**Replicate 3**

NC IPF

M No Tx. Trp No Tx. Trp

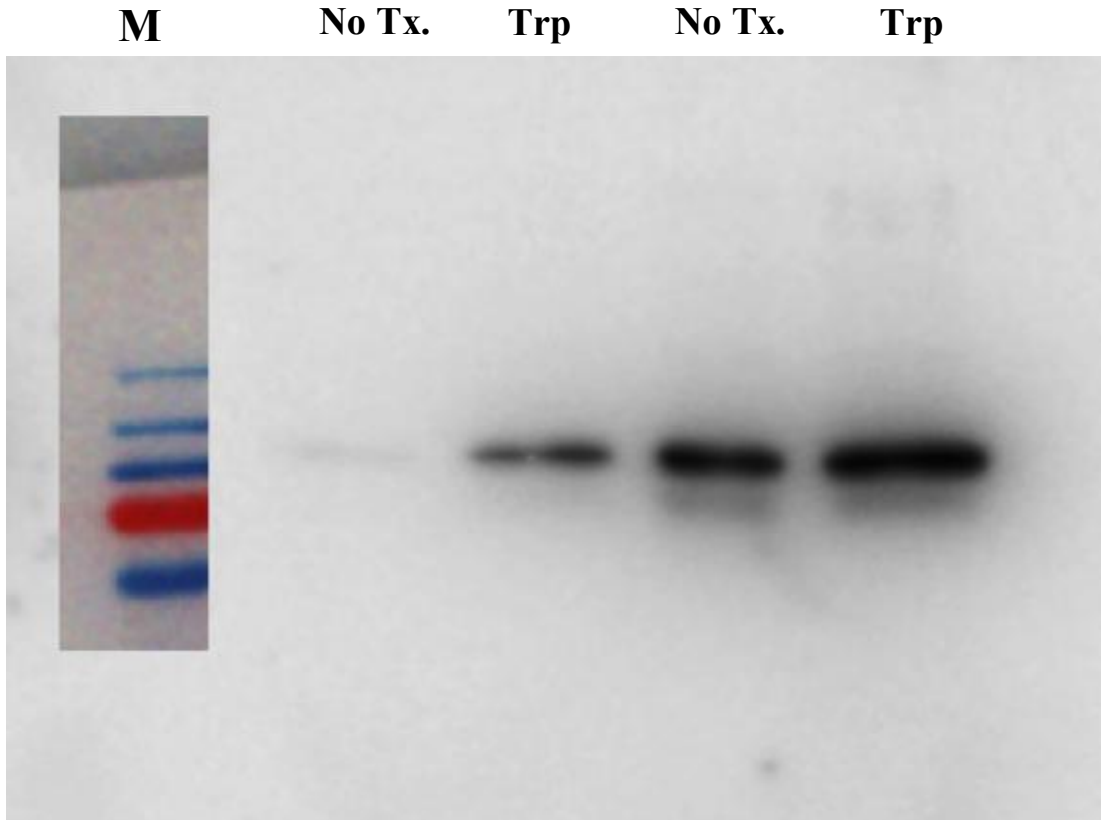

130 kDa

**Replicate 4**

NC IPF

M No Tx. Trp No Tx. Trp

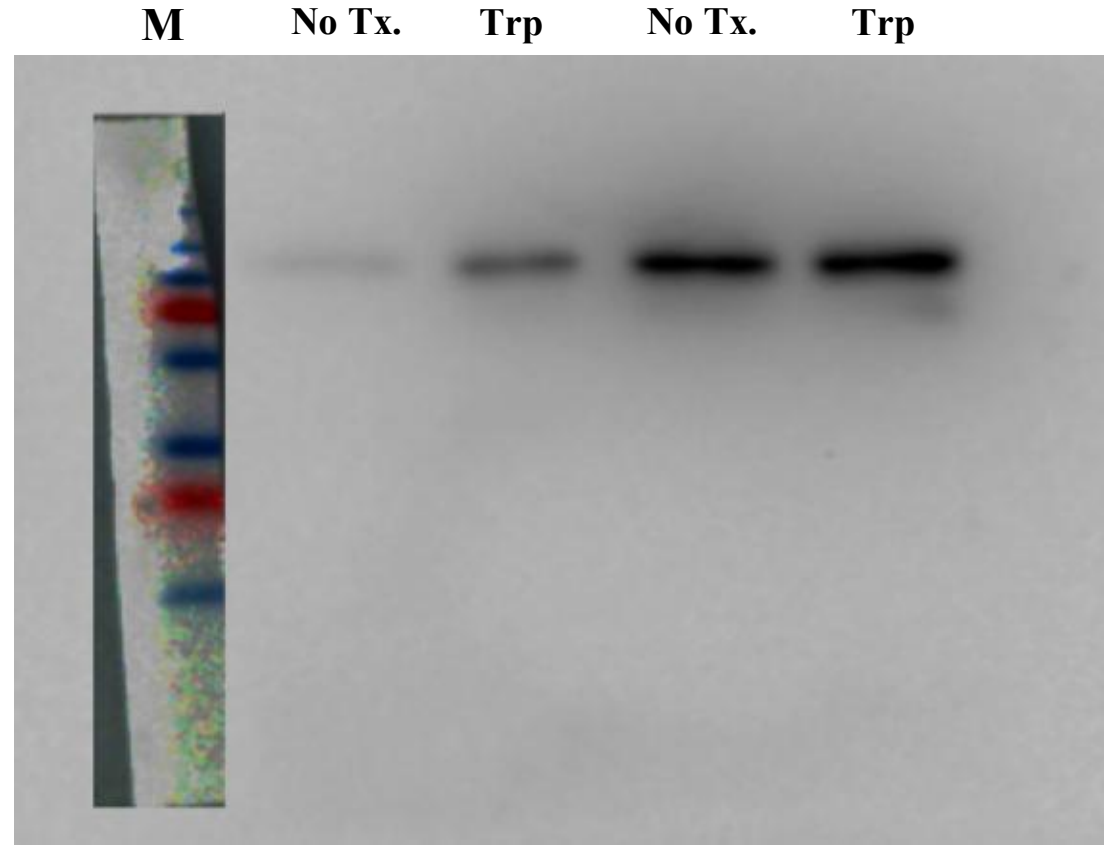

130 kDa

**Fig. 2a. α-SMA. Original uncropped chemiluminescence images.**

The loading order and identity of experimental samples are indicated above each image. The molecular weight marker lane (M) is indicated where applicable. The expected molecular weight of fibronectin is approximately 45 kDa. All images were captured using a ChemiDoc™ Touch Imaging System (Bio-Rad Laboratories, Hercules, CA, USA).

**Replicate 1**

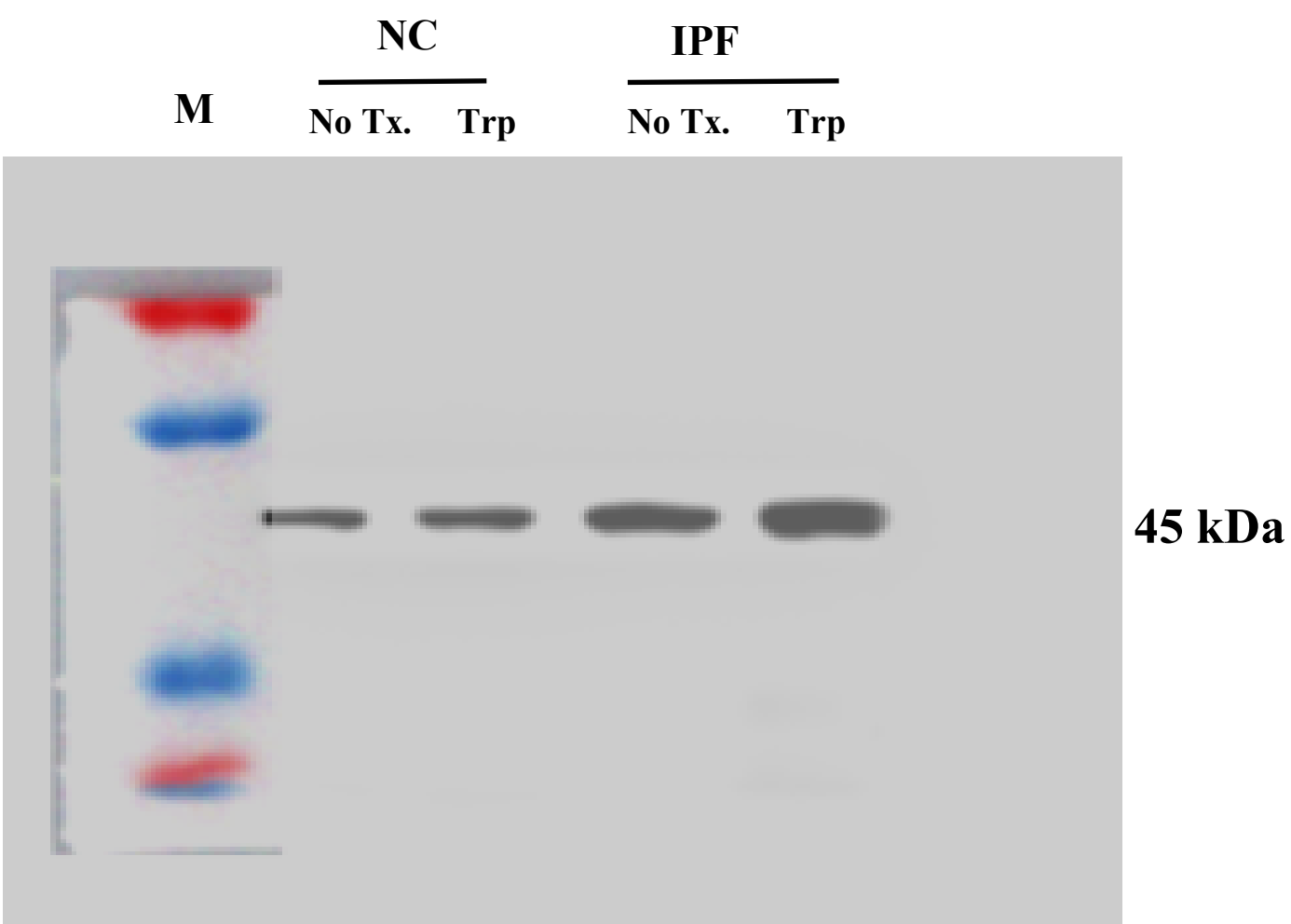


**Replicate 2**

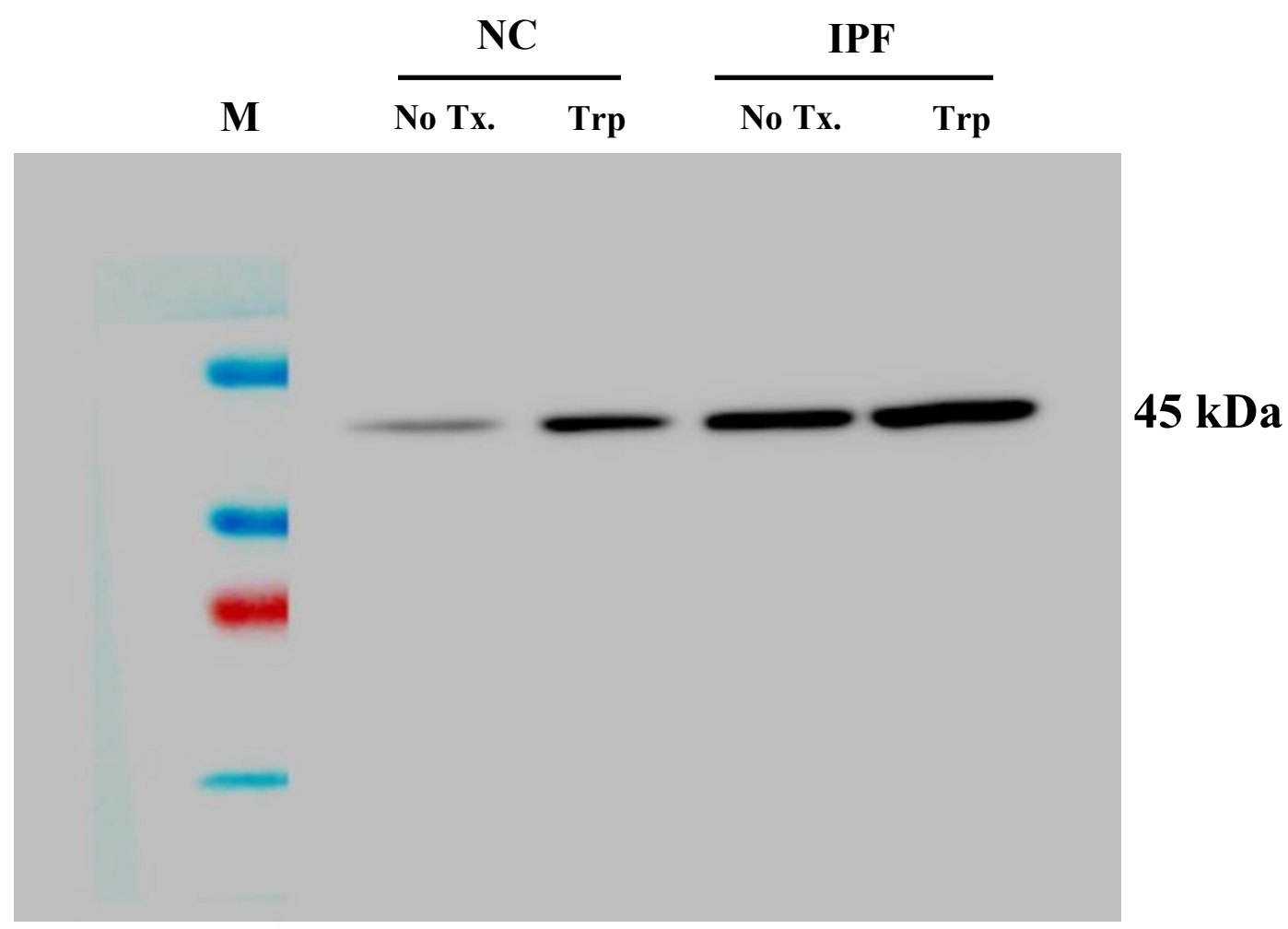


**Replicate 3**

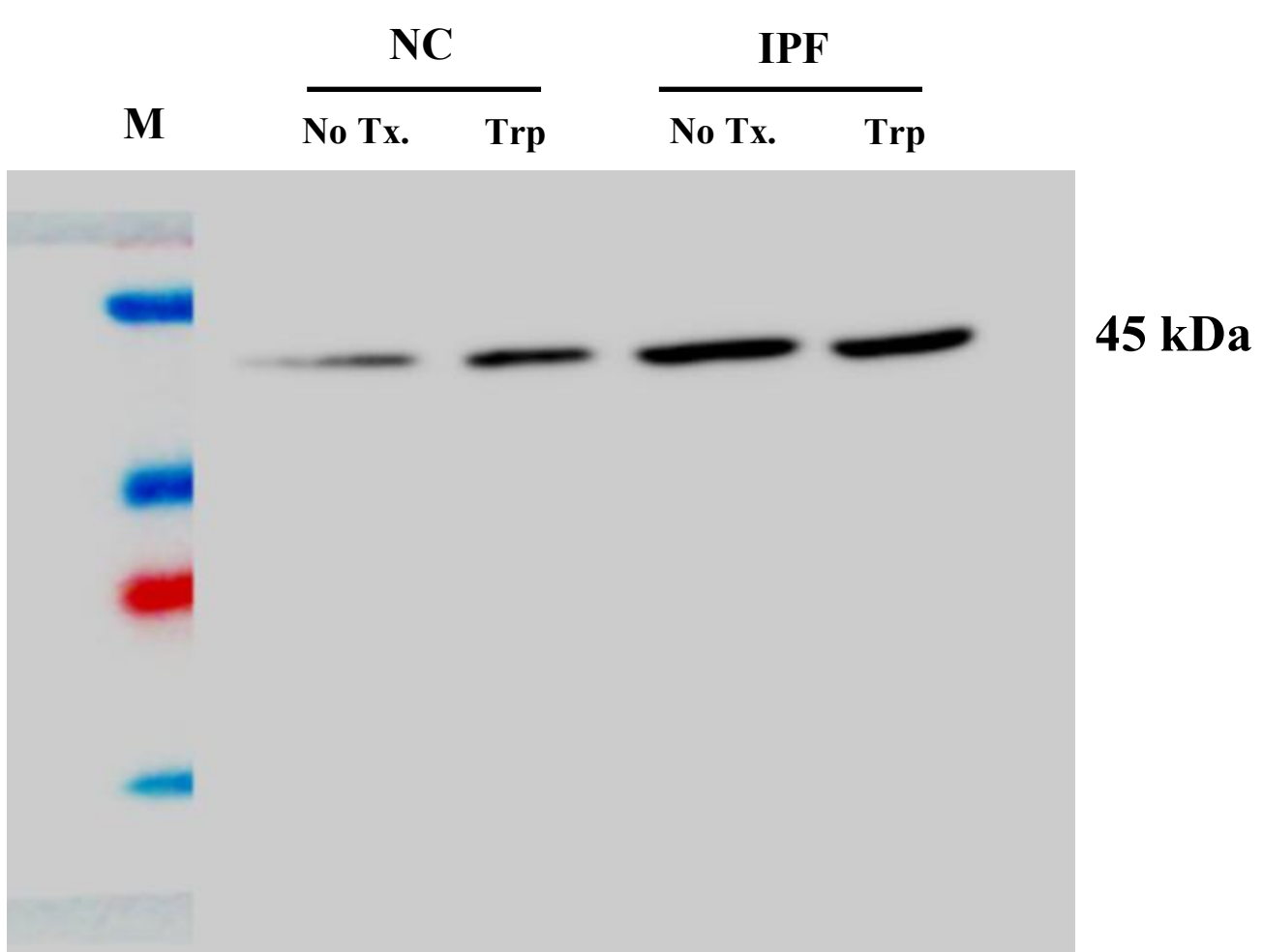


**Replicate 4**

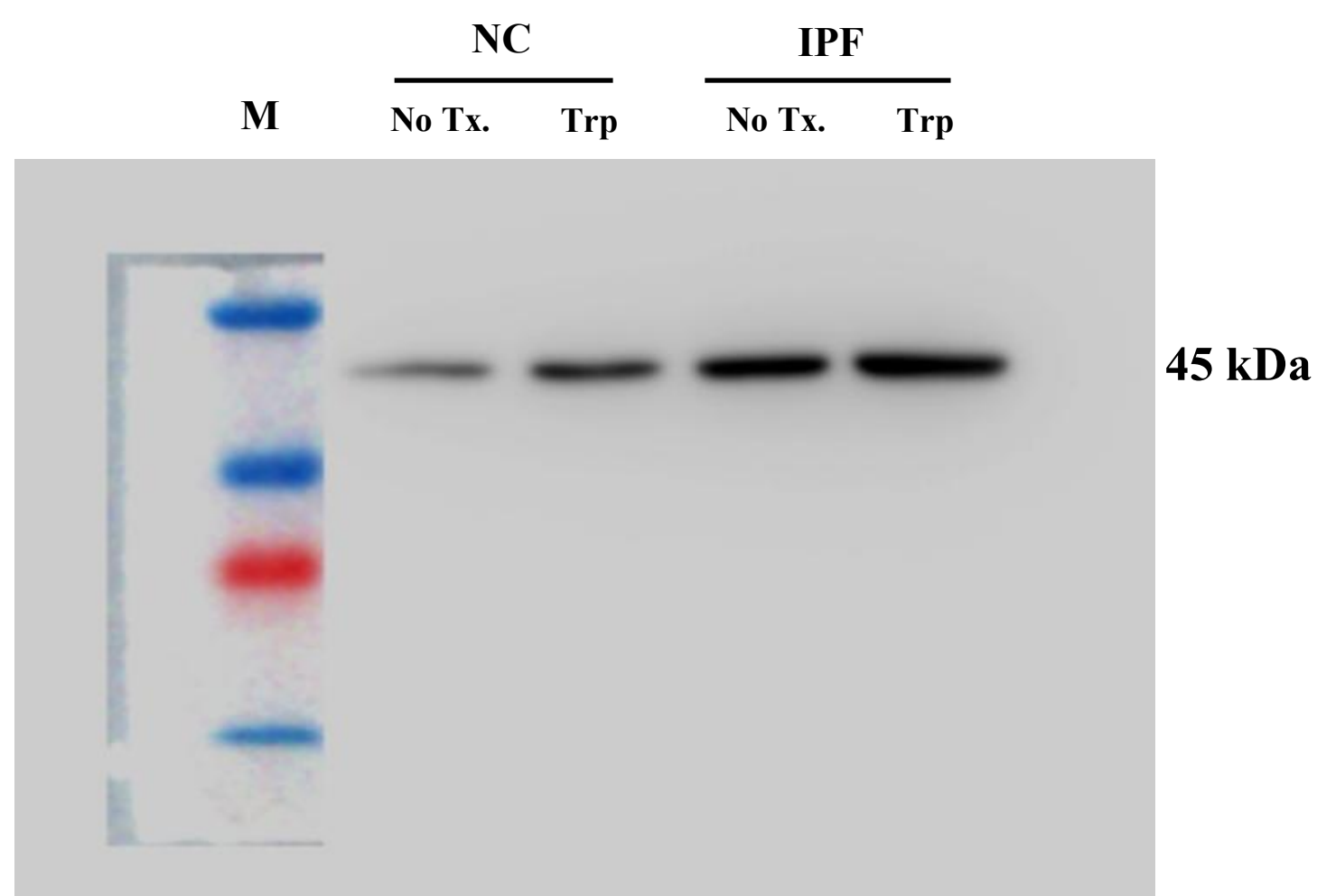

**Fig. 2a. β-actin. Original uncropped chemiluminescence images.**

The loading order and identity of experimental samples are indicated above each image. The molecular weight marker lane (M) is indicated where applicable. The expected molecular weight of fibronectin is approximately 45 kDa. All images were captured using a ChemiDoc™ Touch Imaging System (Bio-Rad Laboratories, Hercules, CA, USA).

**Replicate 1**

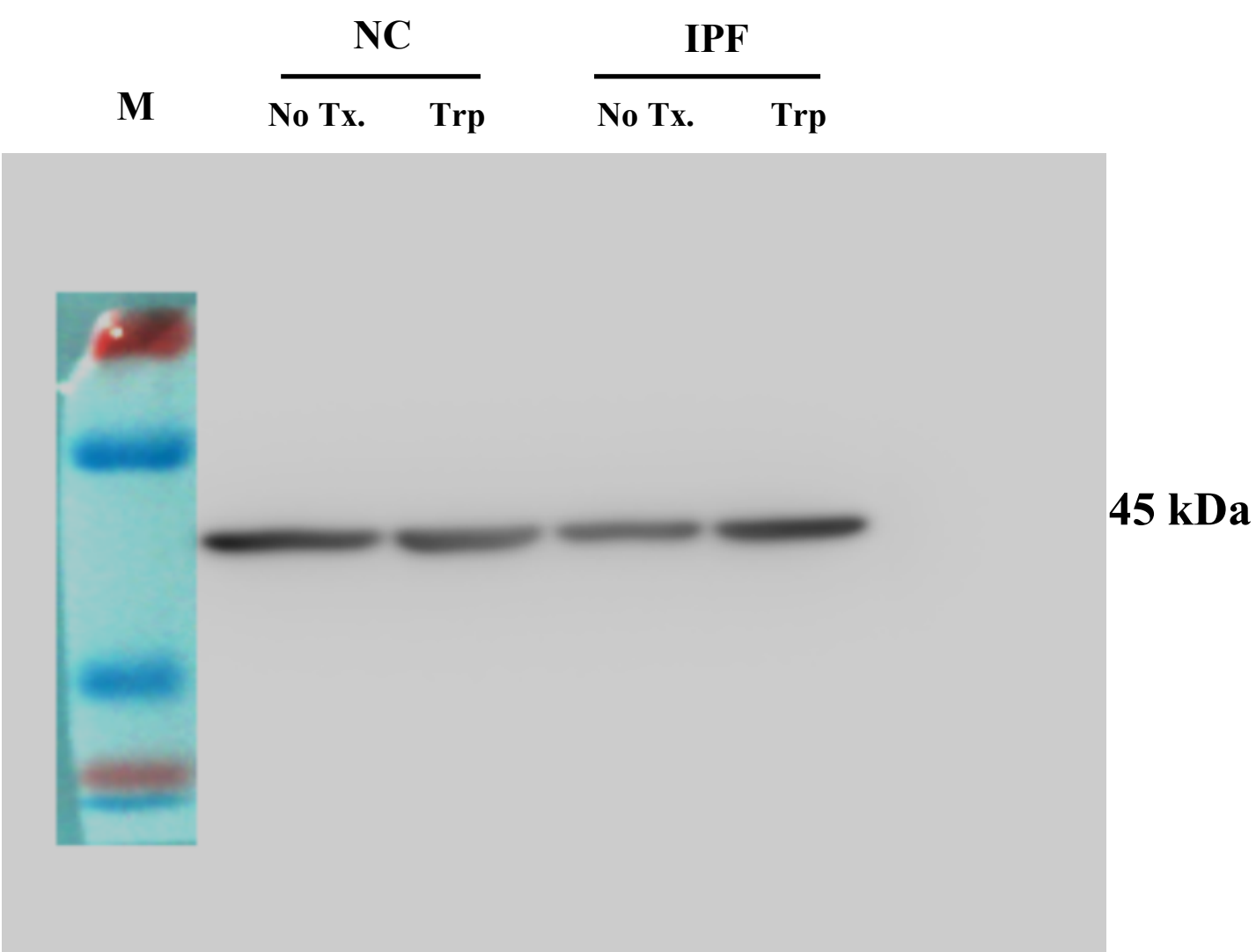


**Replicate 2**

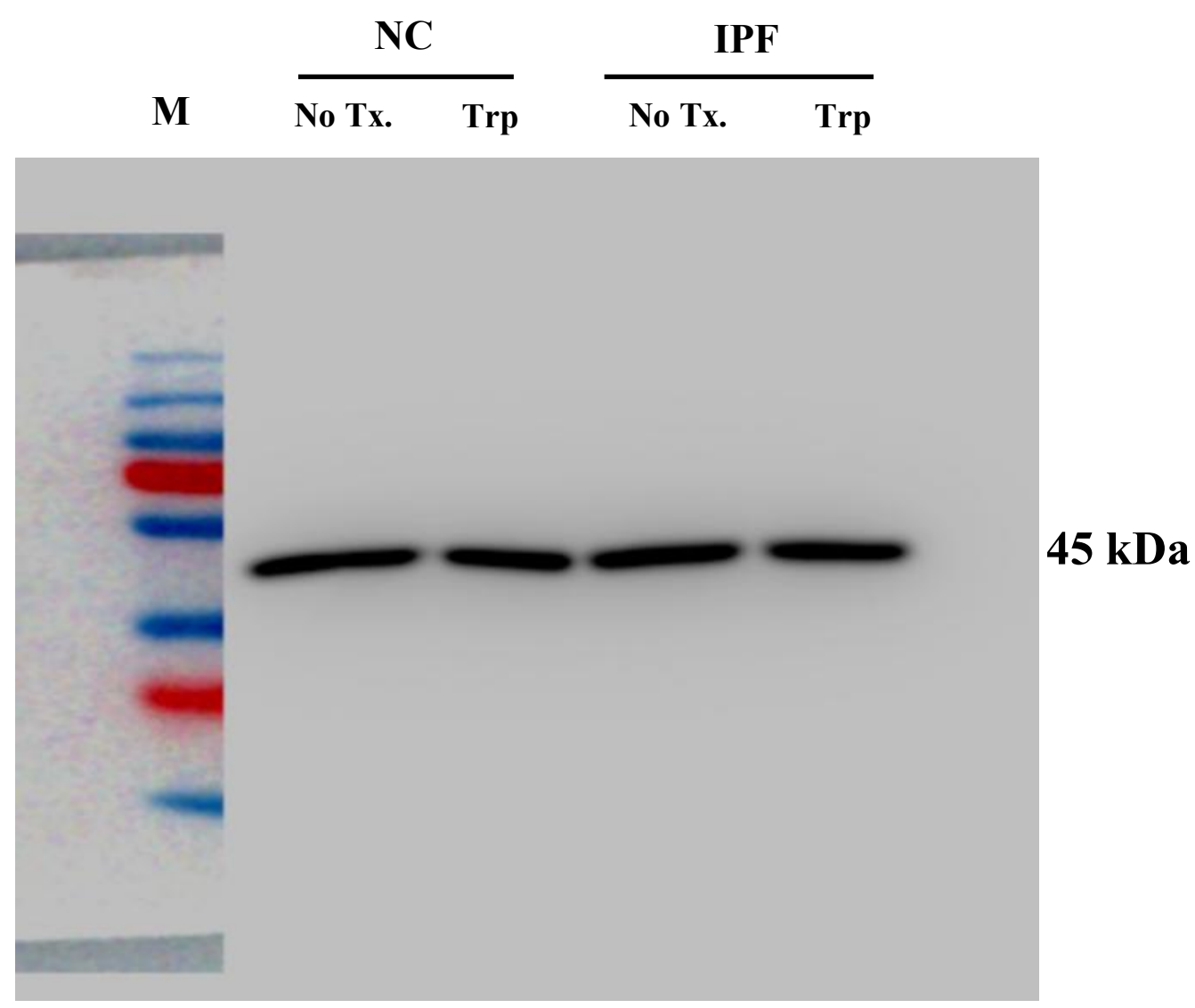


**Replicate 3**

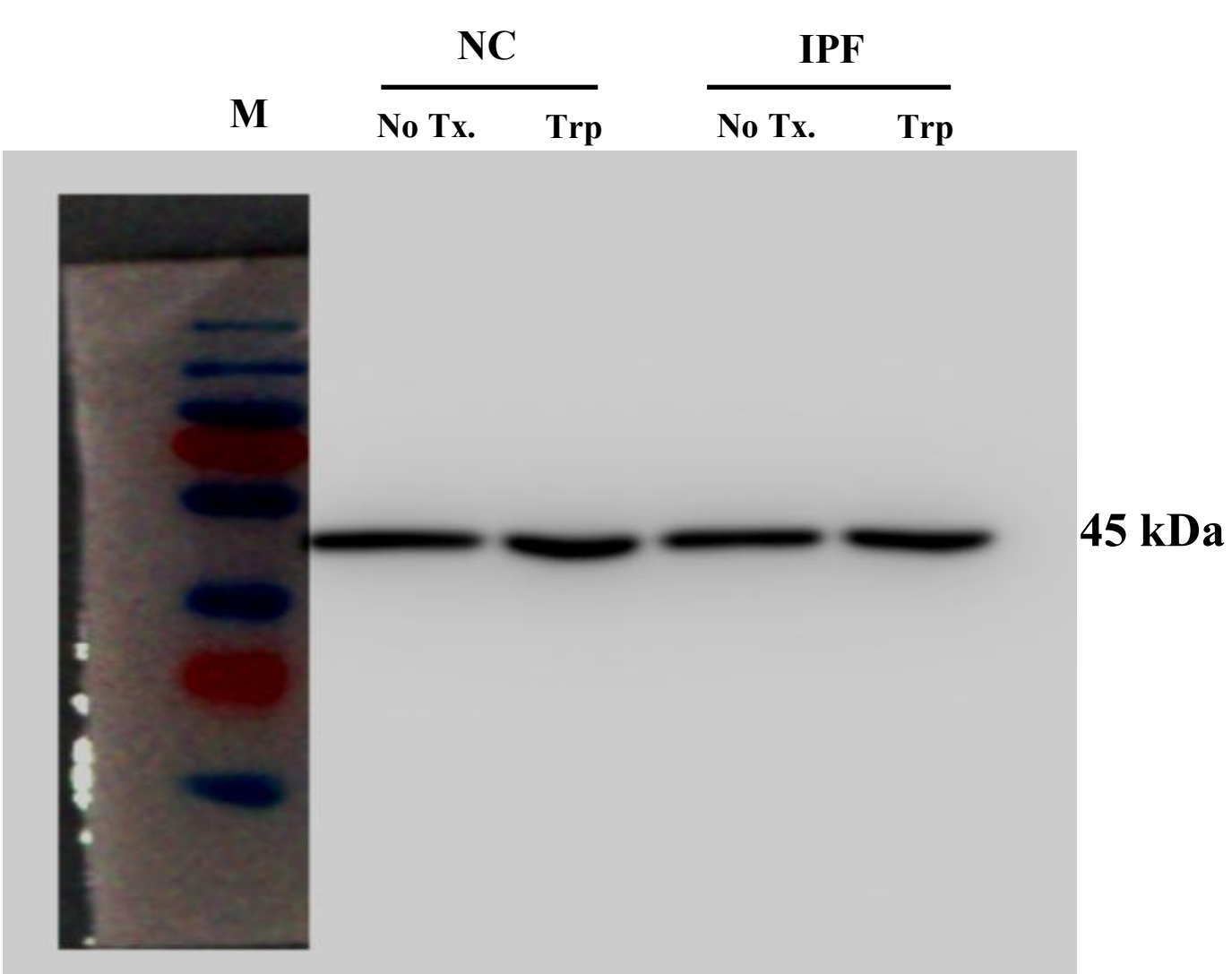


**Replicate 4**

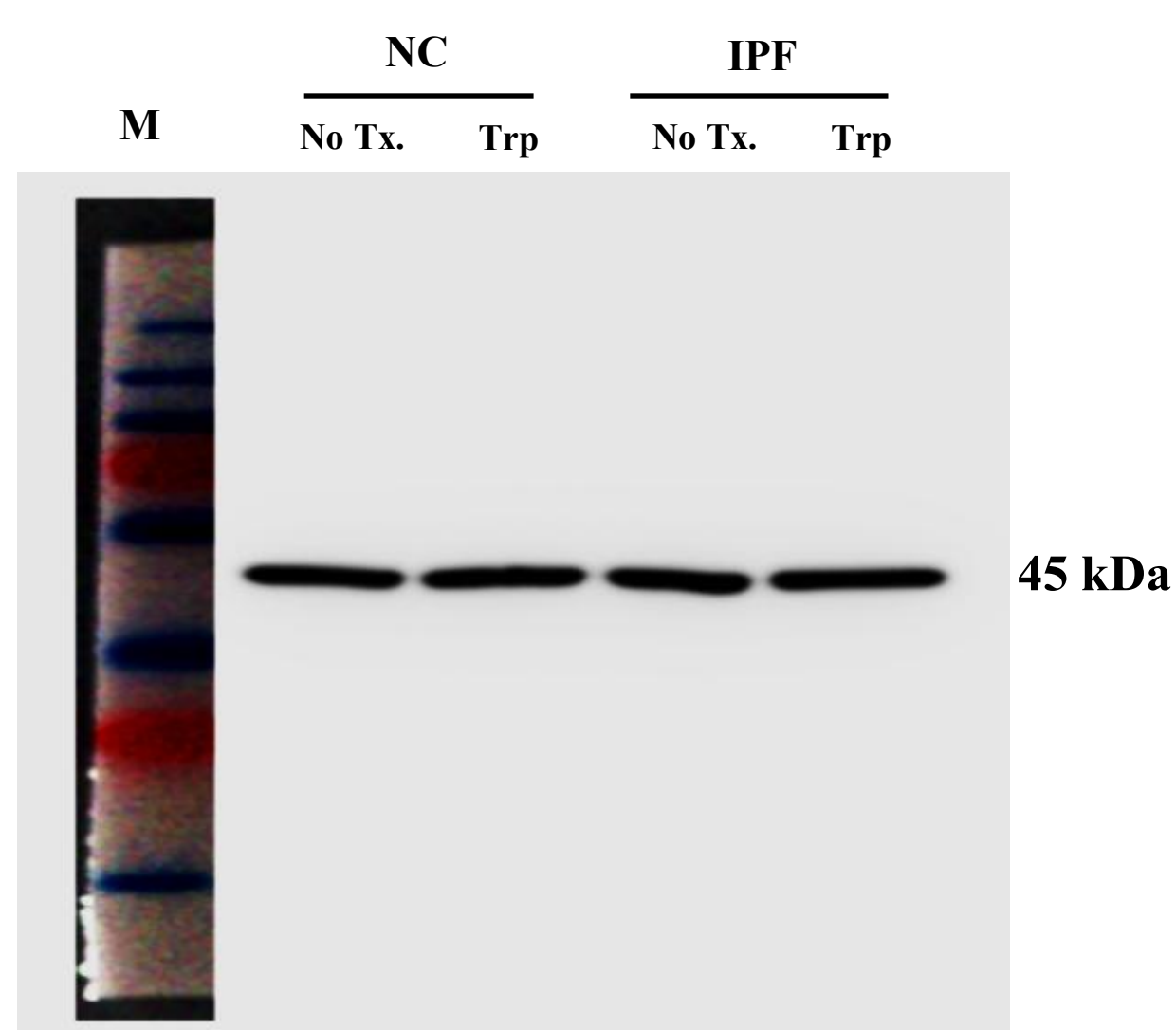

**Fig. 2b. Fibronectin. Original uncropped chemiluminescence images.**
The loading order and identity of experimental samples are indicated above each image. The molecular weight marker lane (M) is indicated where applicable. Lanes marked with "X" indicate lanes that were present in the original blot but were not included in the final figure. The expected molecular weight of fibronectin is approximately 260 kDa. All images were captured using a ChemiDoc™ Touch Imaging System (Bio-Rad Laboratories, Hercules, CA, USA).

**Replicate 1**

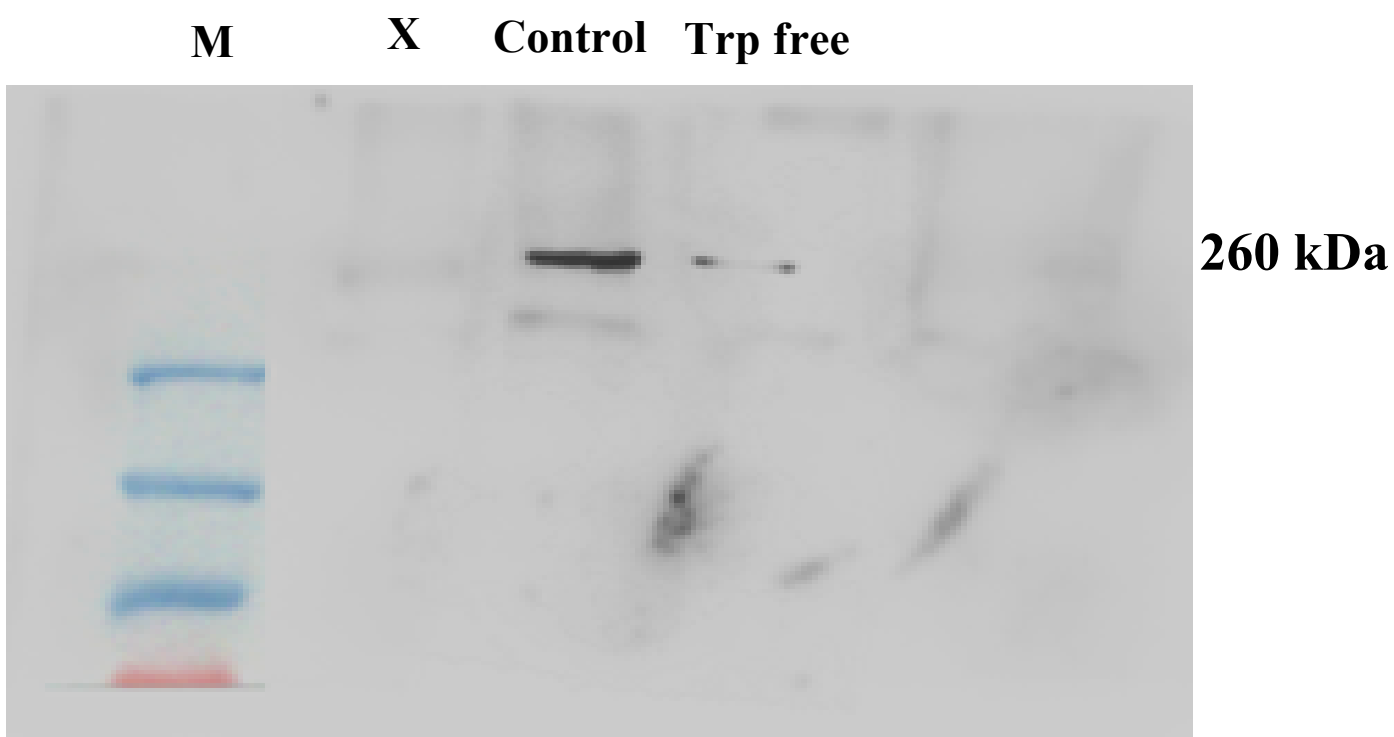


**Replicate 2**

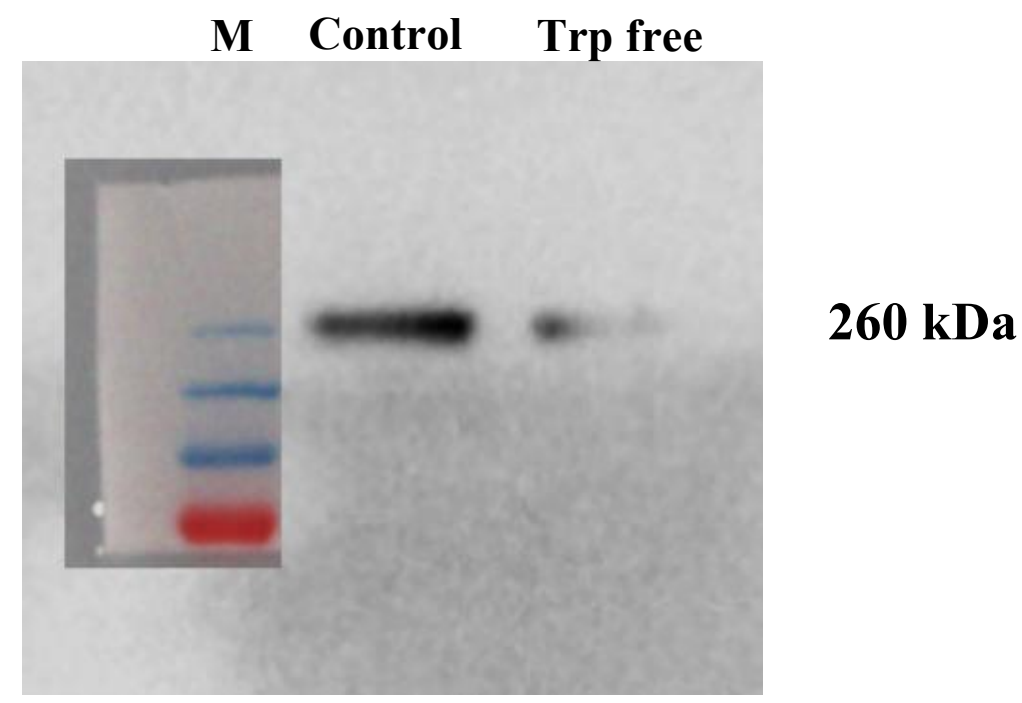


**Replicate 3**

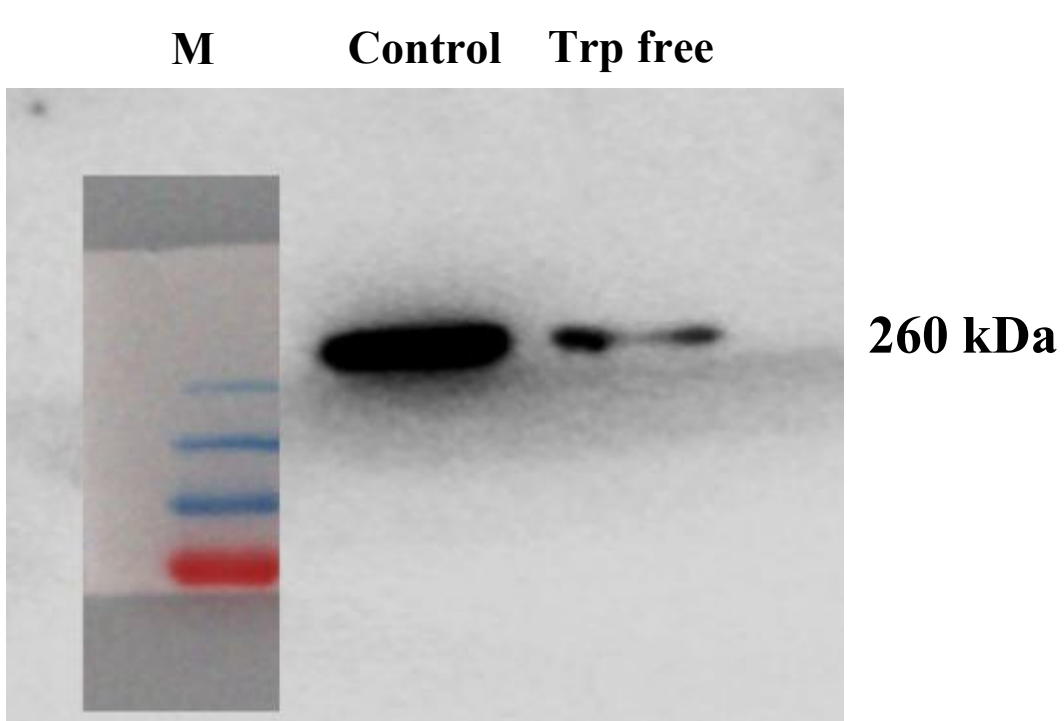


**Replicate 4**

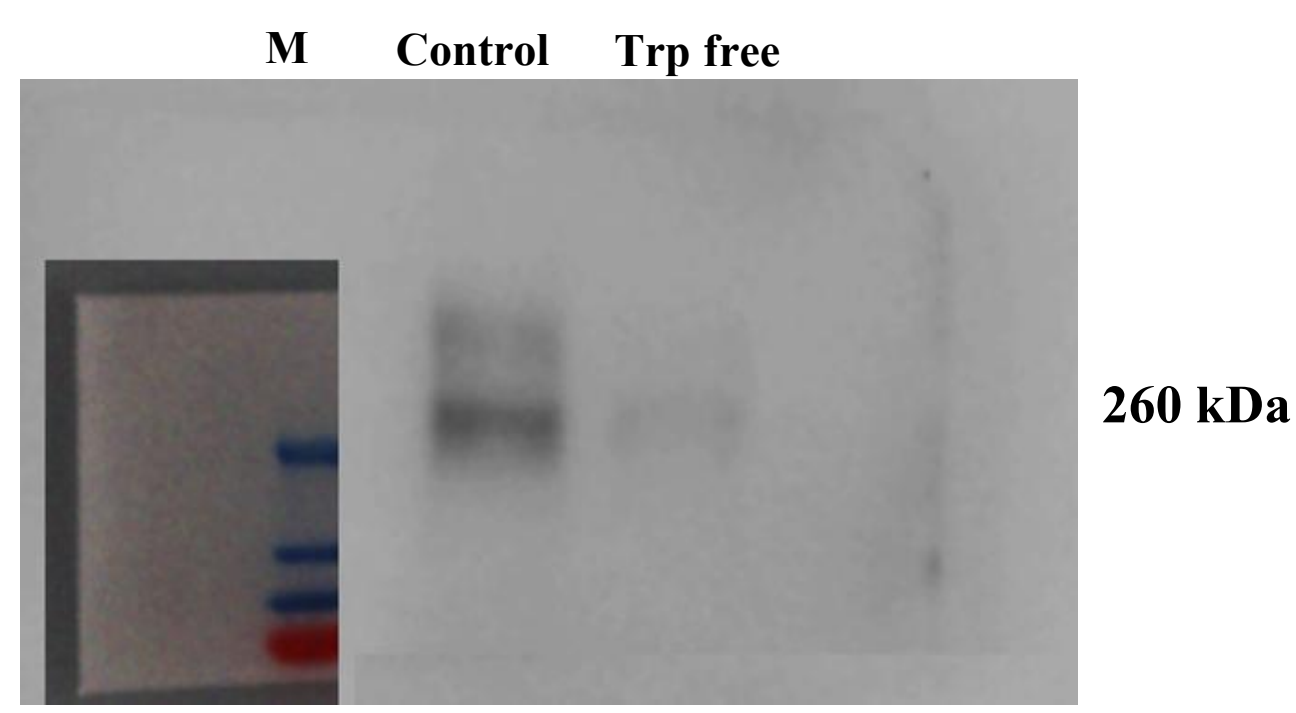

**Fig. 2b. Collagen. Original uncropped chemiluminescence images.**

The loading order and identity of experimental samples are indicated above each image. The molecular weight marker lane (M) is indicated where applicable. Lanes marked with "X" indicate lanes that were present in the original blot but were not included in the final figure. The expected molecular weight of fibronectin is approximately 130 kDa. All images were captured using a ChemiDoc™ Touch Imaging System (Bio-Rad Laboratories, Hercules, CA, USA).

**Replicate 1**

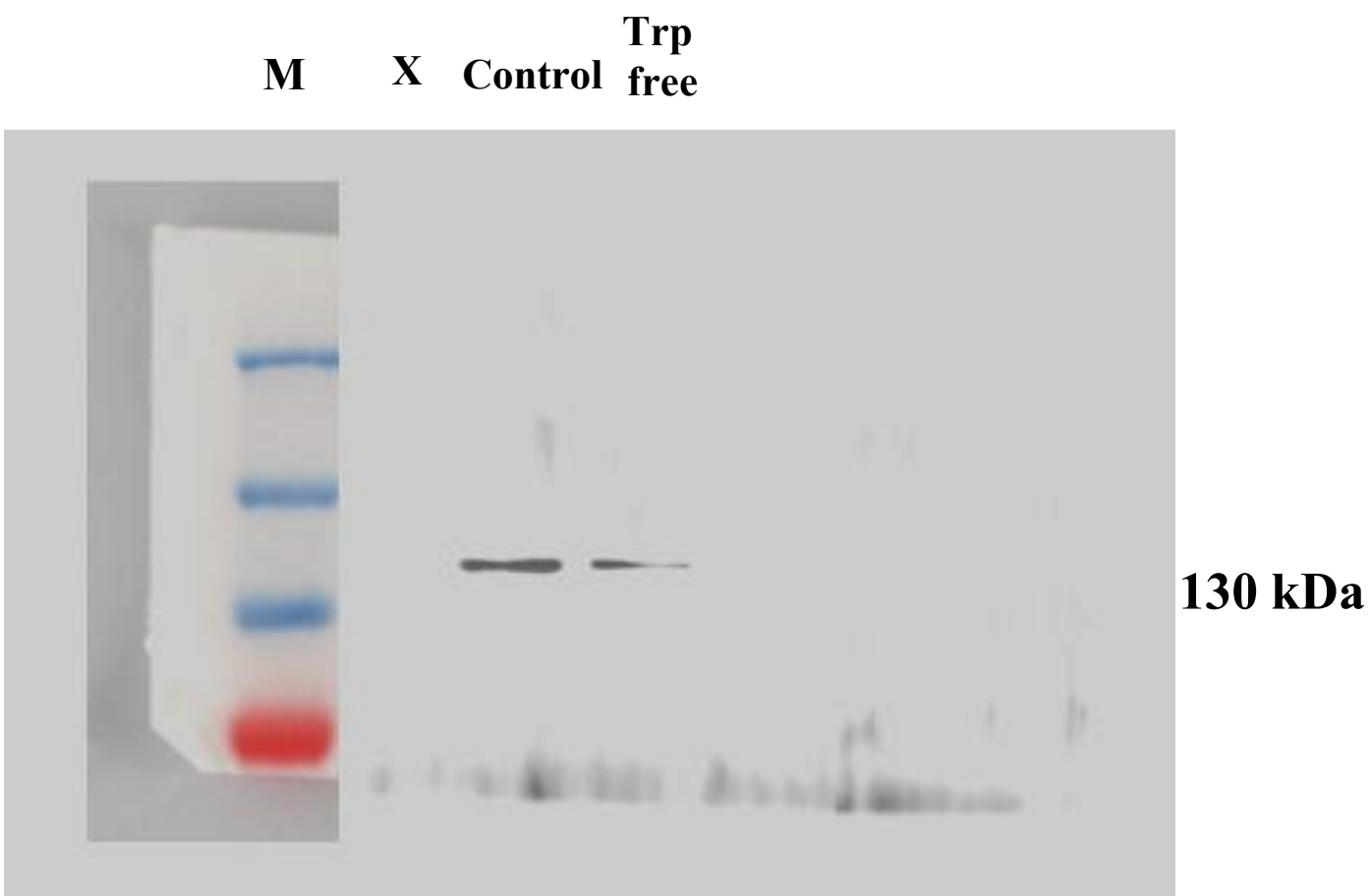


**Replicate 2**

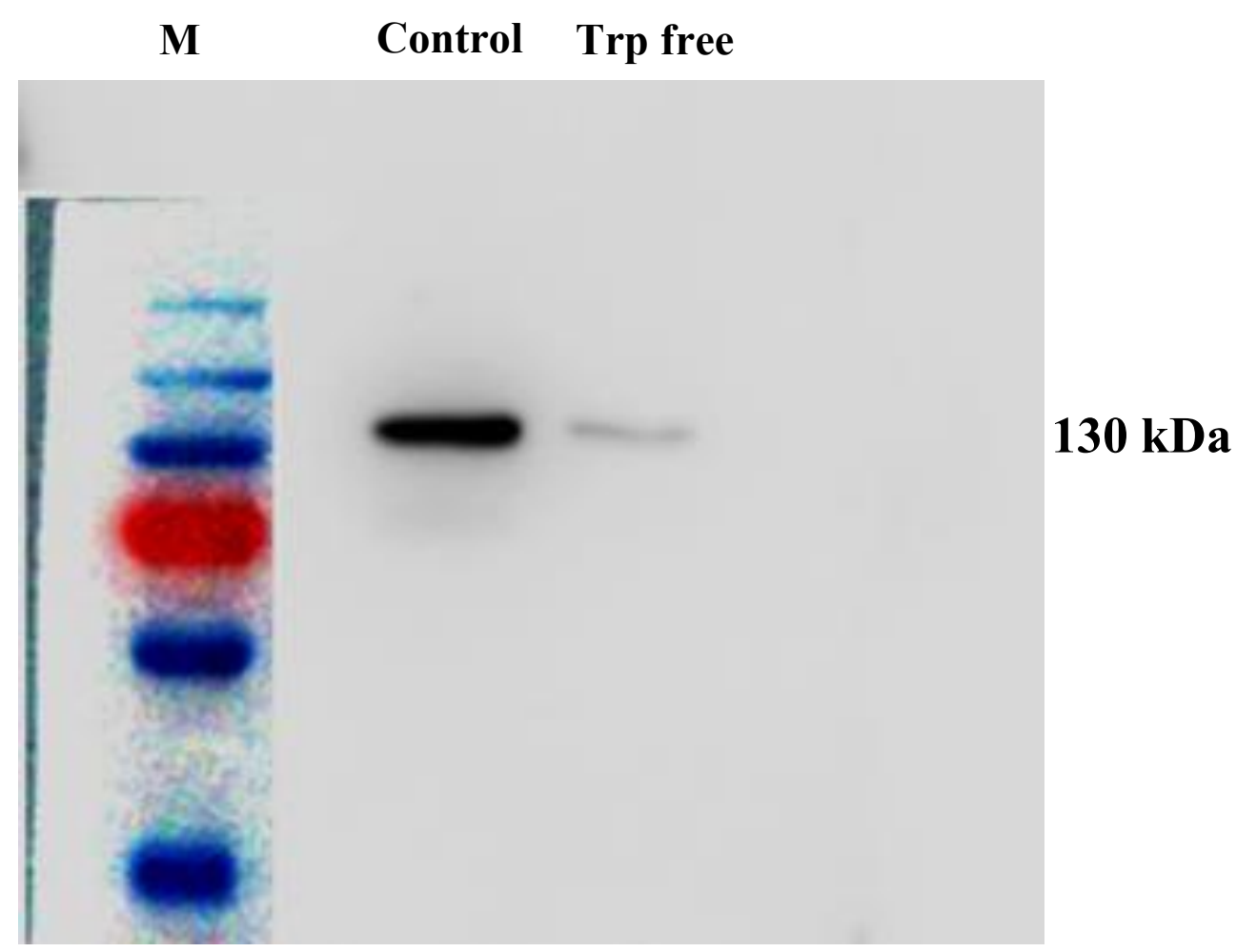


**Replicate 3**

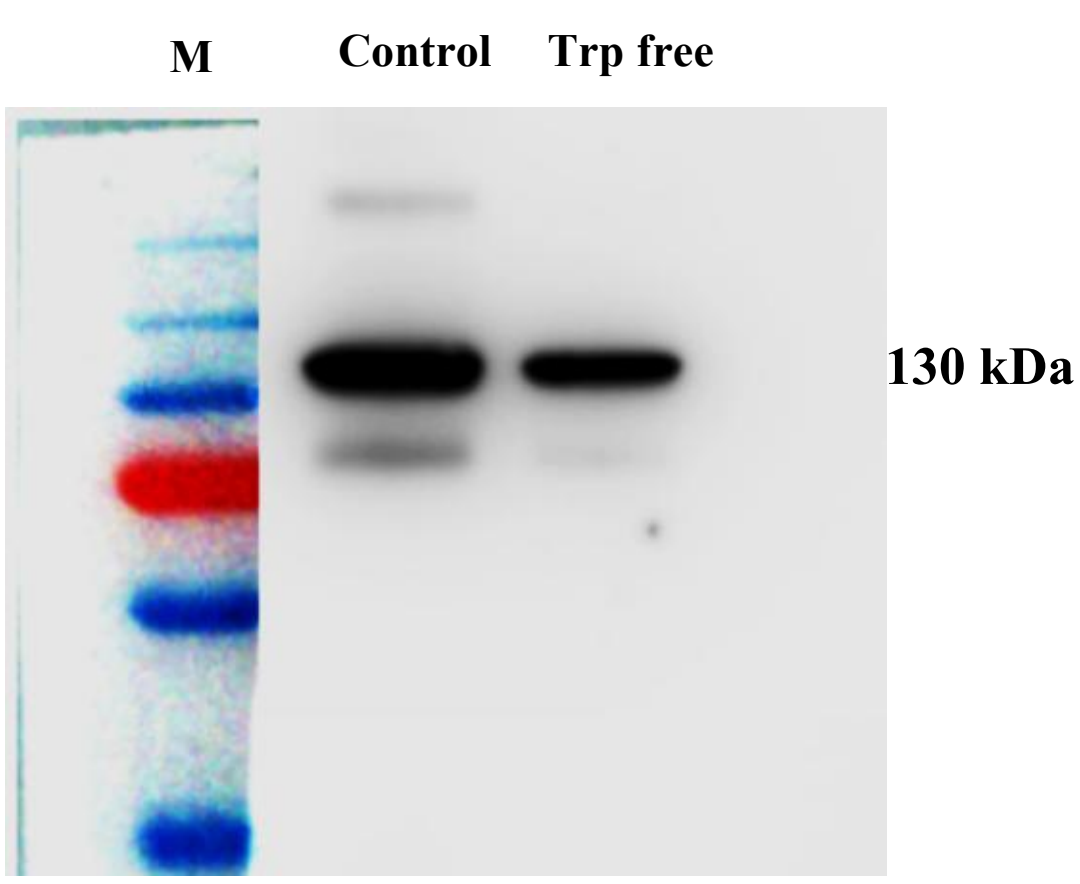


**Replicate 4**

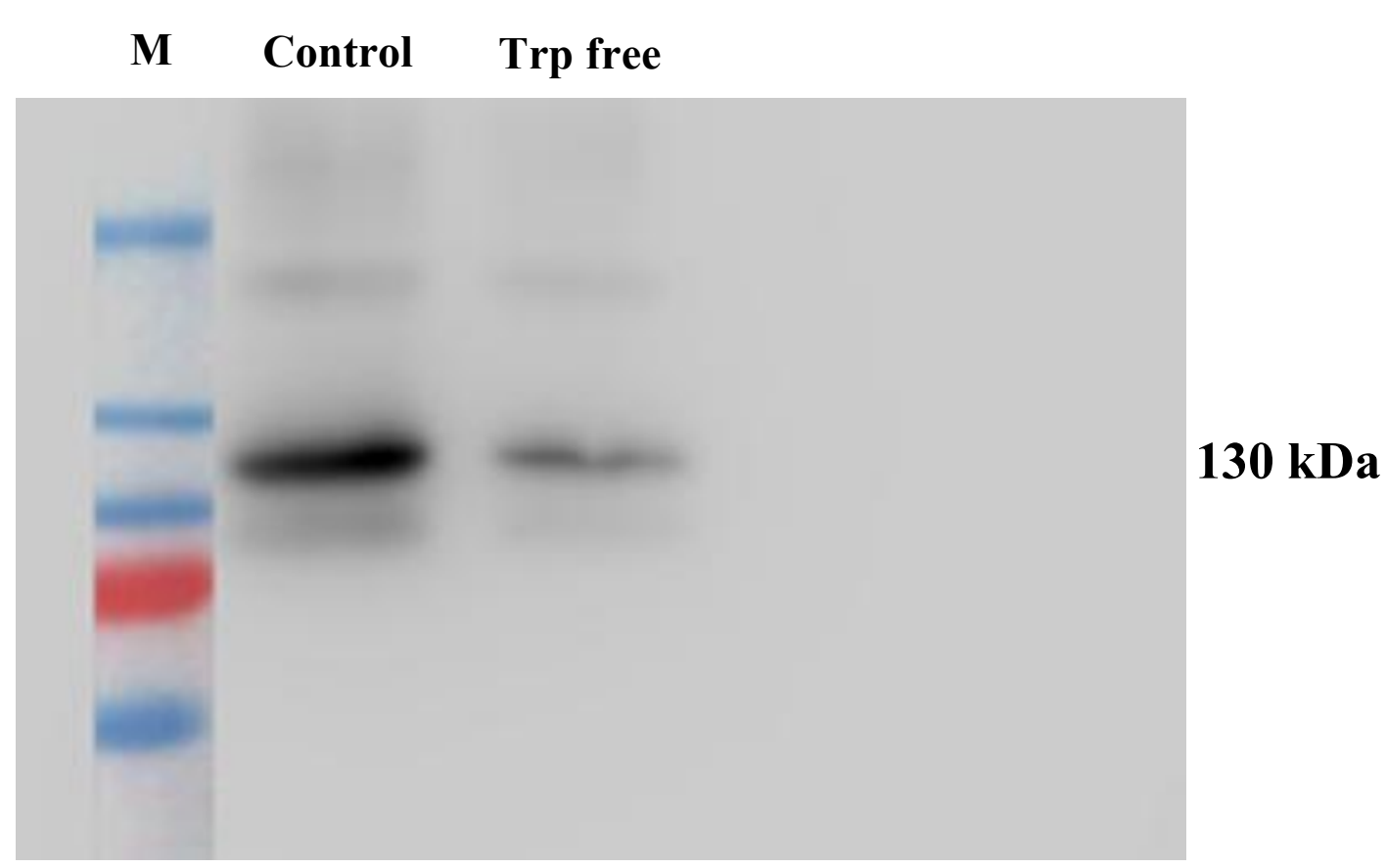

**Fig. 2b. α-SMA. Original uncropped chemiluminescence images.**

The loading order and identity of experimental samples are indicated above each image. The molecular weight marker lane (M) is indicated where applicable. Lanes marked with “X” indicate lanes that were present in the original blot but were not included in the final figure. The expected molecular weight of fibronectin is approximately 45 kDa. All images were captured using a ChemiDoc™ Touch Imaging System (Bio-Rad Laboratories, Hercules, CA, USA).

**Replicate 1**

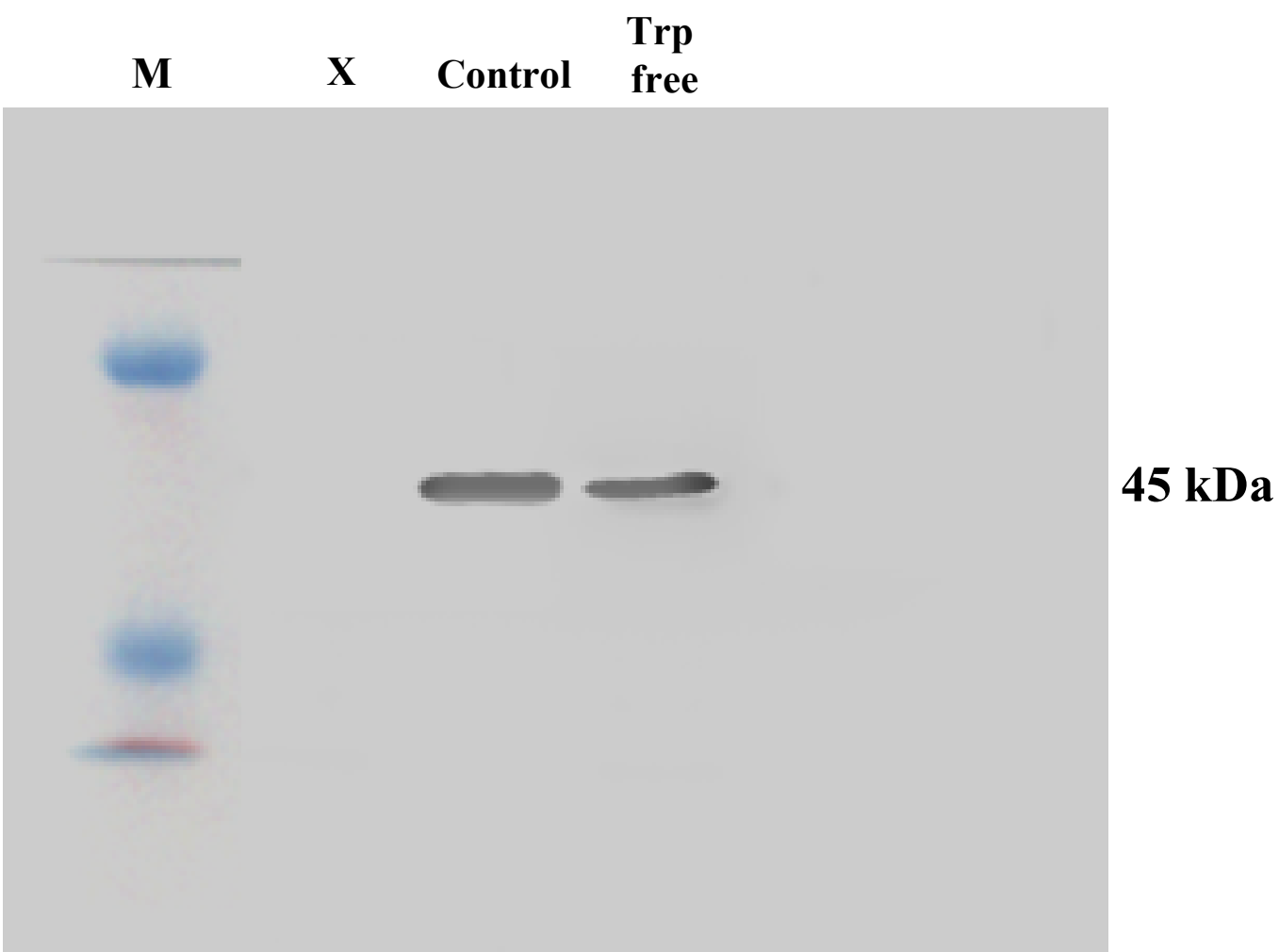


**Replicate 2**

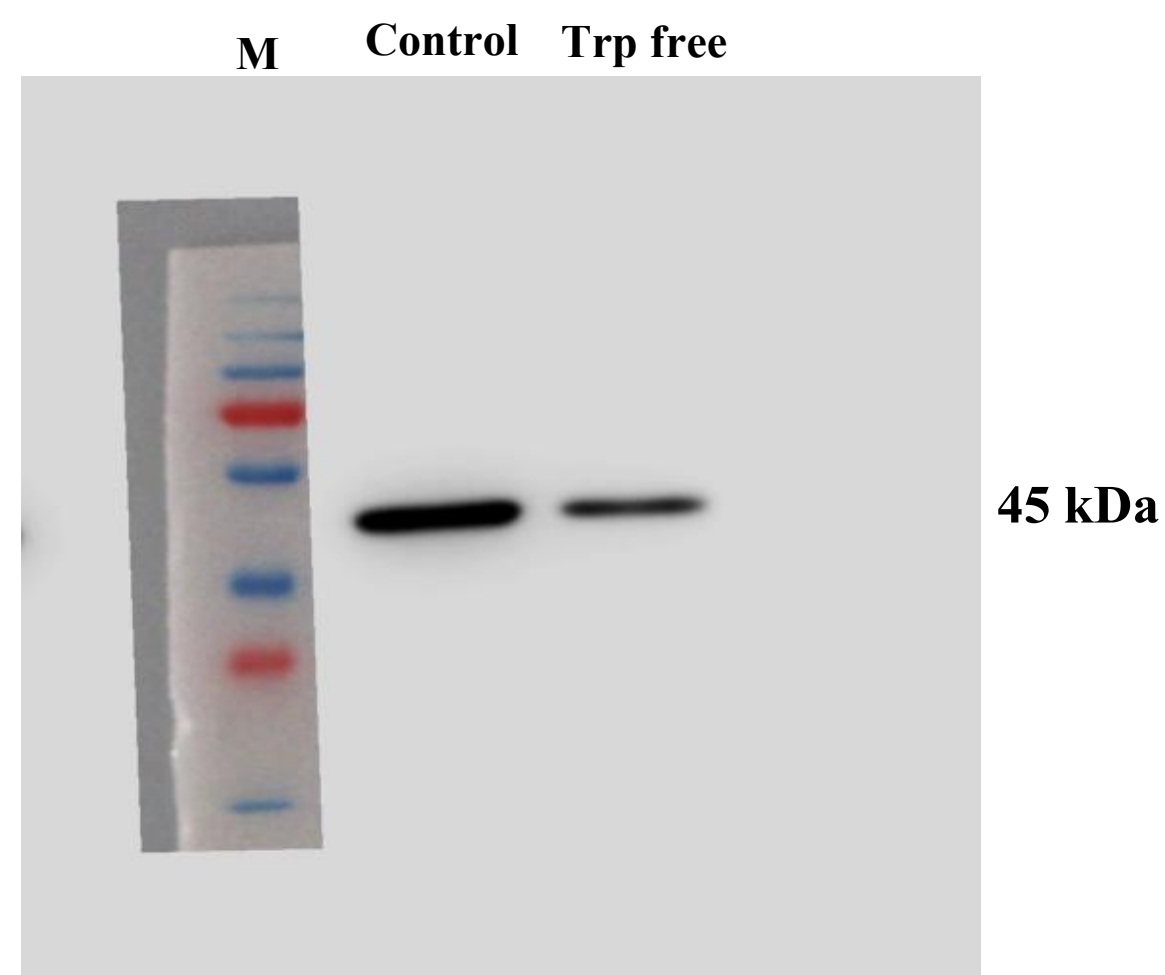


**Replicate 3**

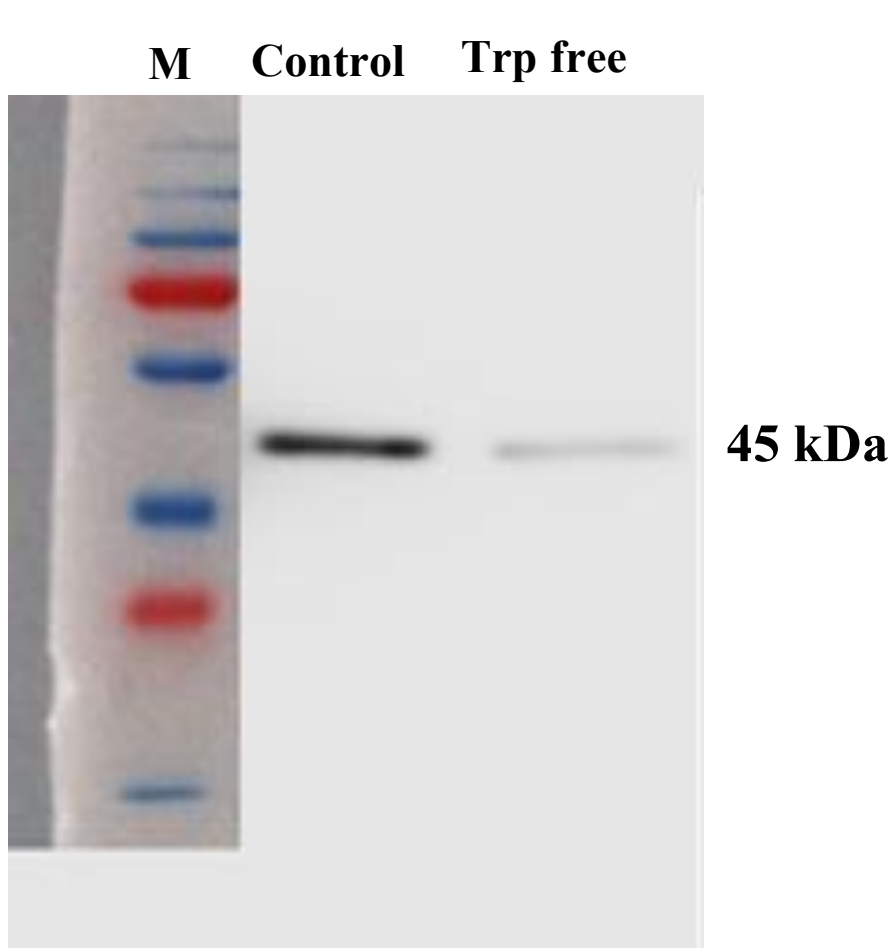


**Replicate 4**

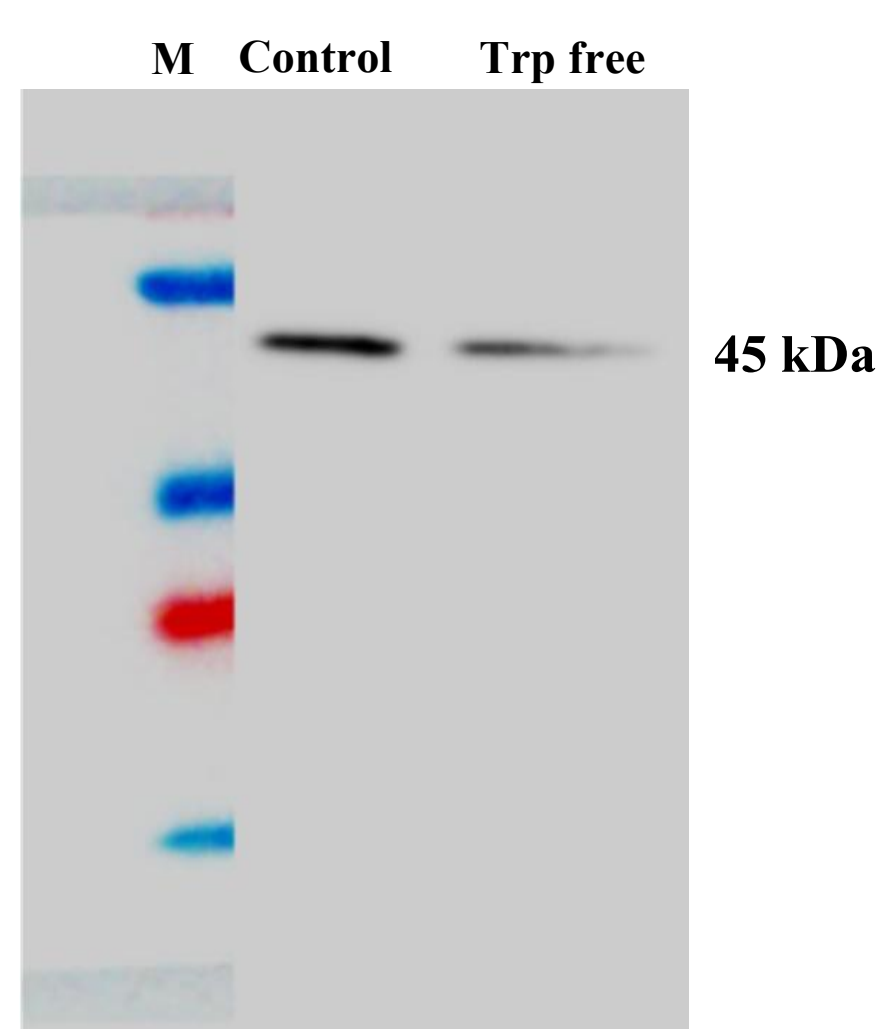

**Fig. 2b. β-actin. Original uncropped chemiluminescence images.**

The loading order and identity of experimental samples are indicated above each image. The molecular weight marker lane (M) is indicated where applicable. Lanes marked with “X” indicate lanes that were present in the original blot but were not included in the final figure. The expected molecular weight of fibronectin is approximately 45 kDa. All images were captured using a ChemiDoc™ Touch Imaging System (Bio-Rad Laboratories, Hercules, CA, USA).

**Replicate 1**

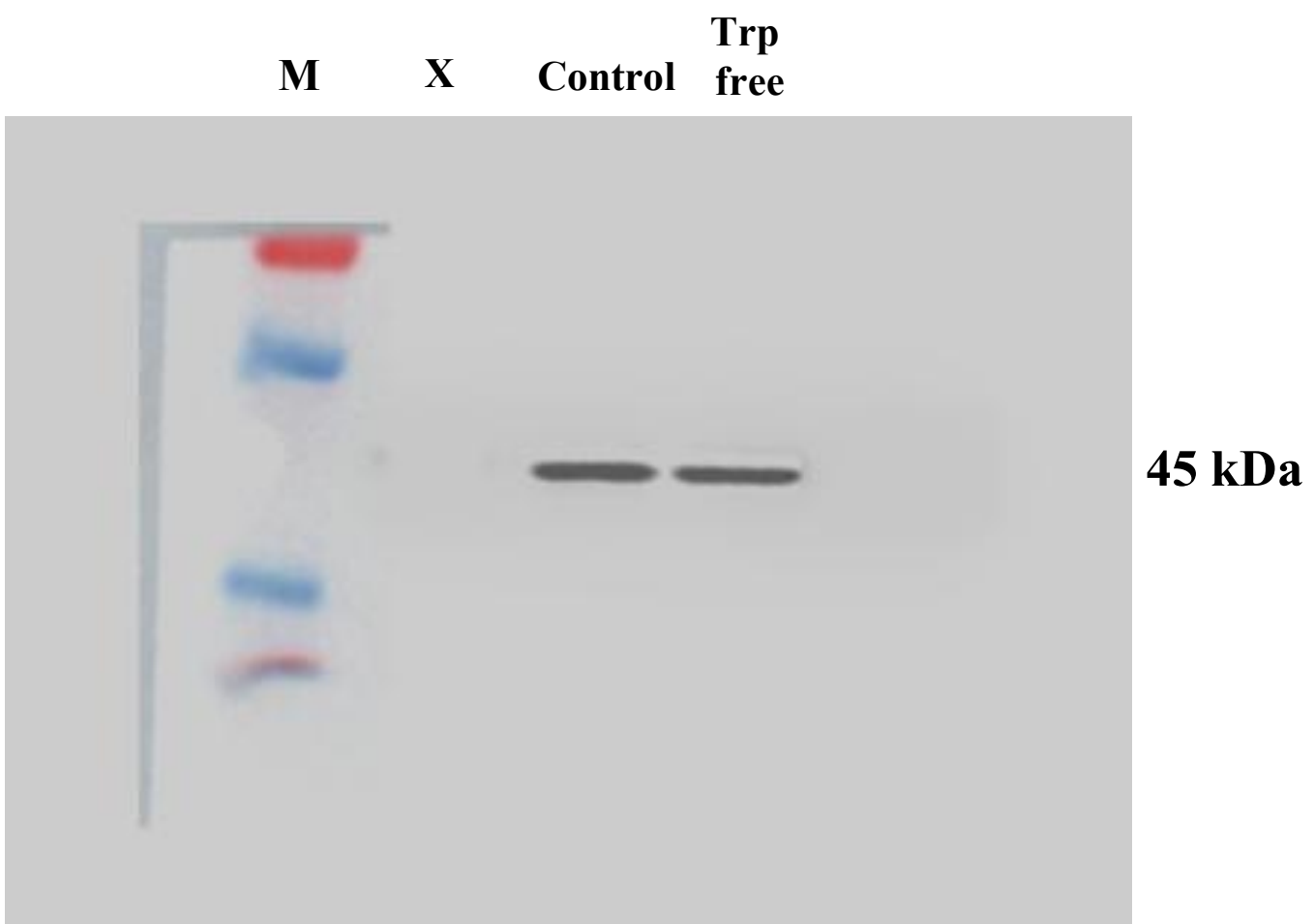


**Replicate 2**

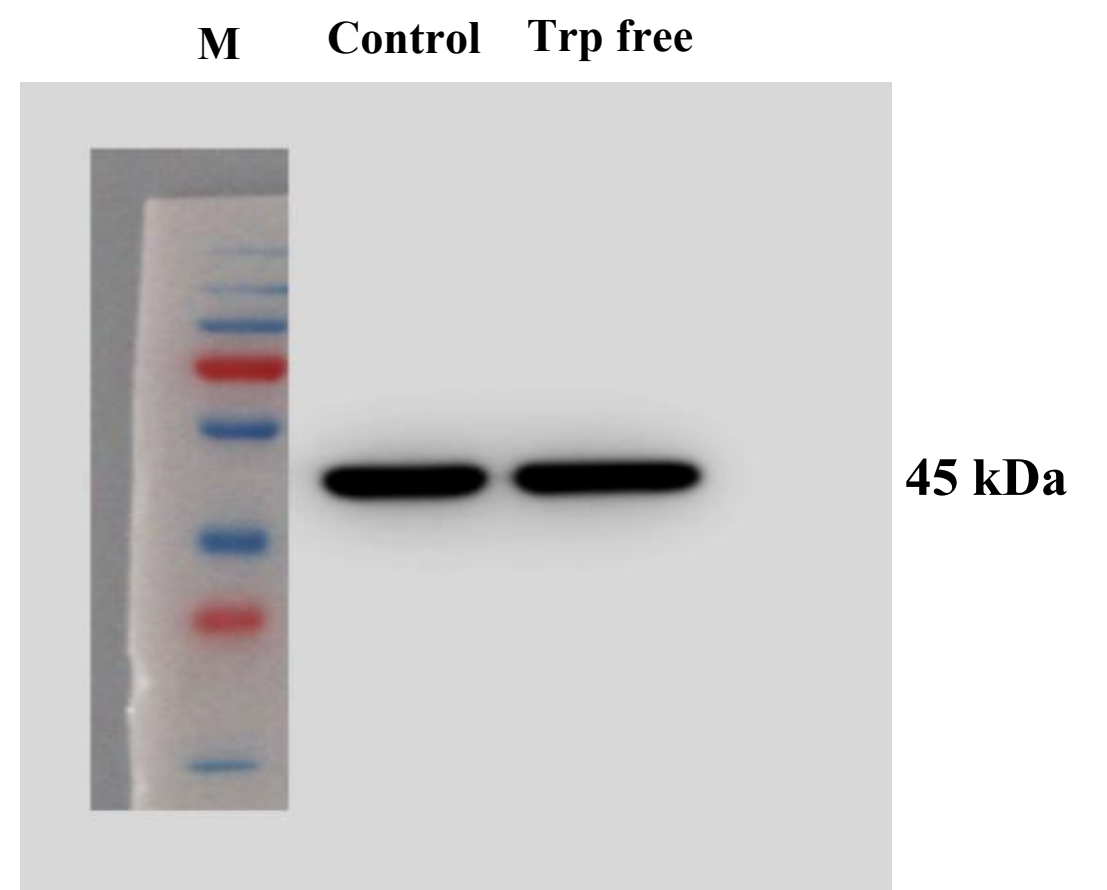


**Replicate 3**

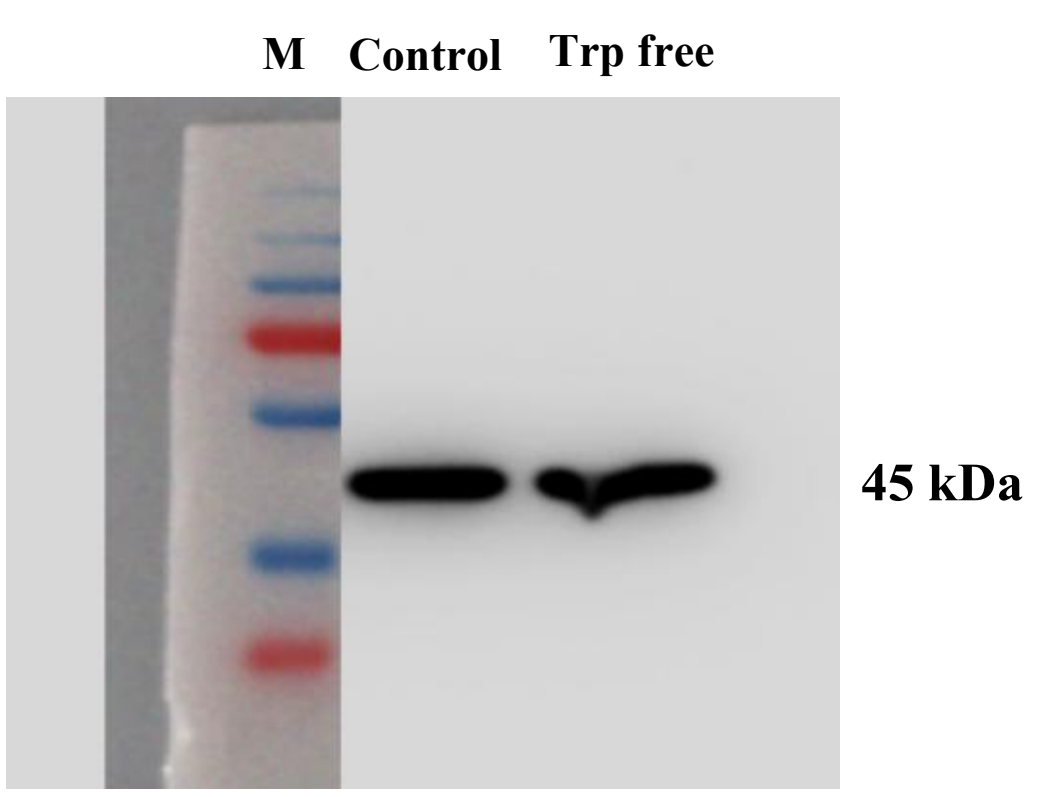


**Replicate 4**

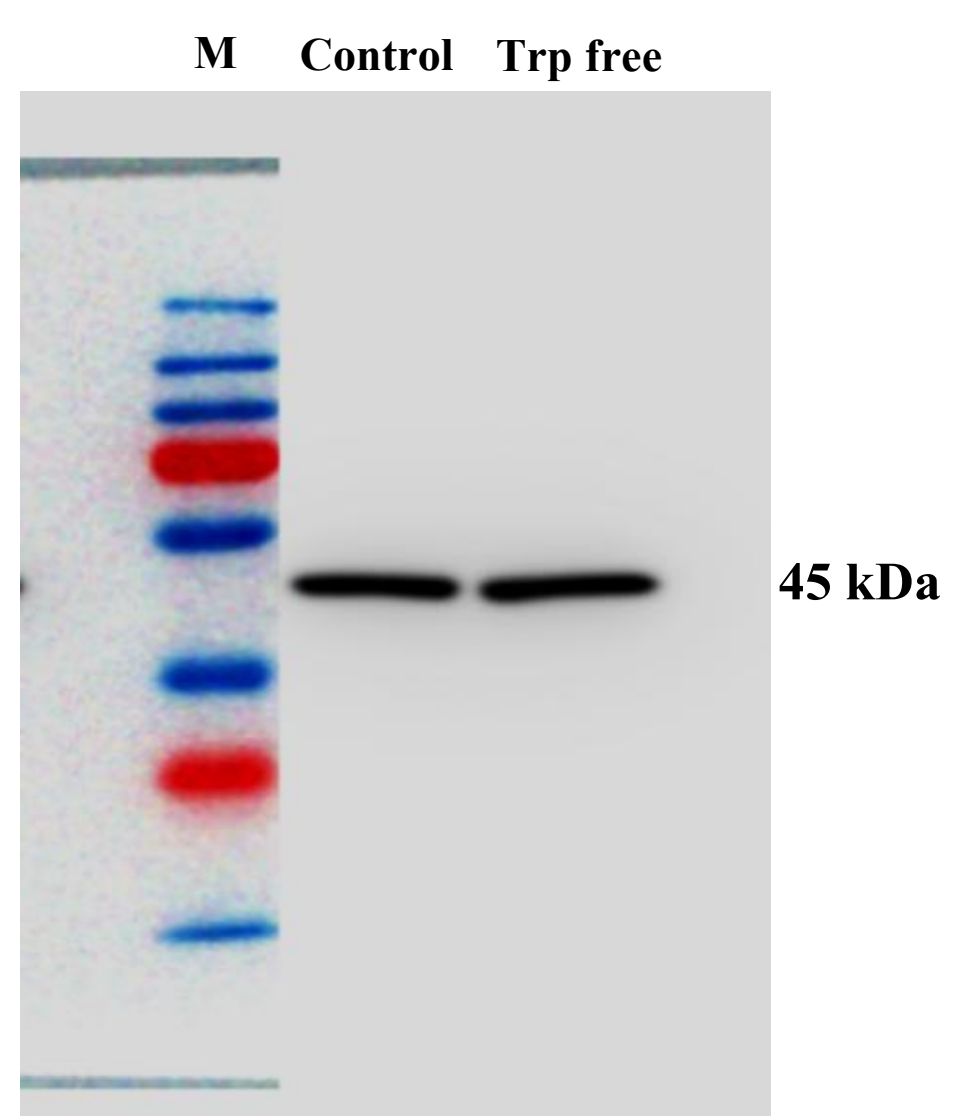

**Fig. 3a. p-Akt. Original uncropped chemiluminescence images.**

The loading order and identity of experimental samples are indicated above each image. The molecular weight marker lane (M) is indicated where applicable. The expected molecular weight of fibronectin is approximately 60 kDa. All images were captured using a ChemiDoc™ Touch Imaging System (Bio-Rad Laboratories, Hercules, CA, USA).

**Replicate 1**

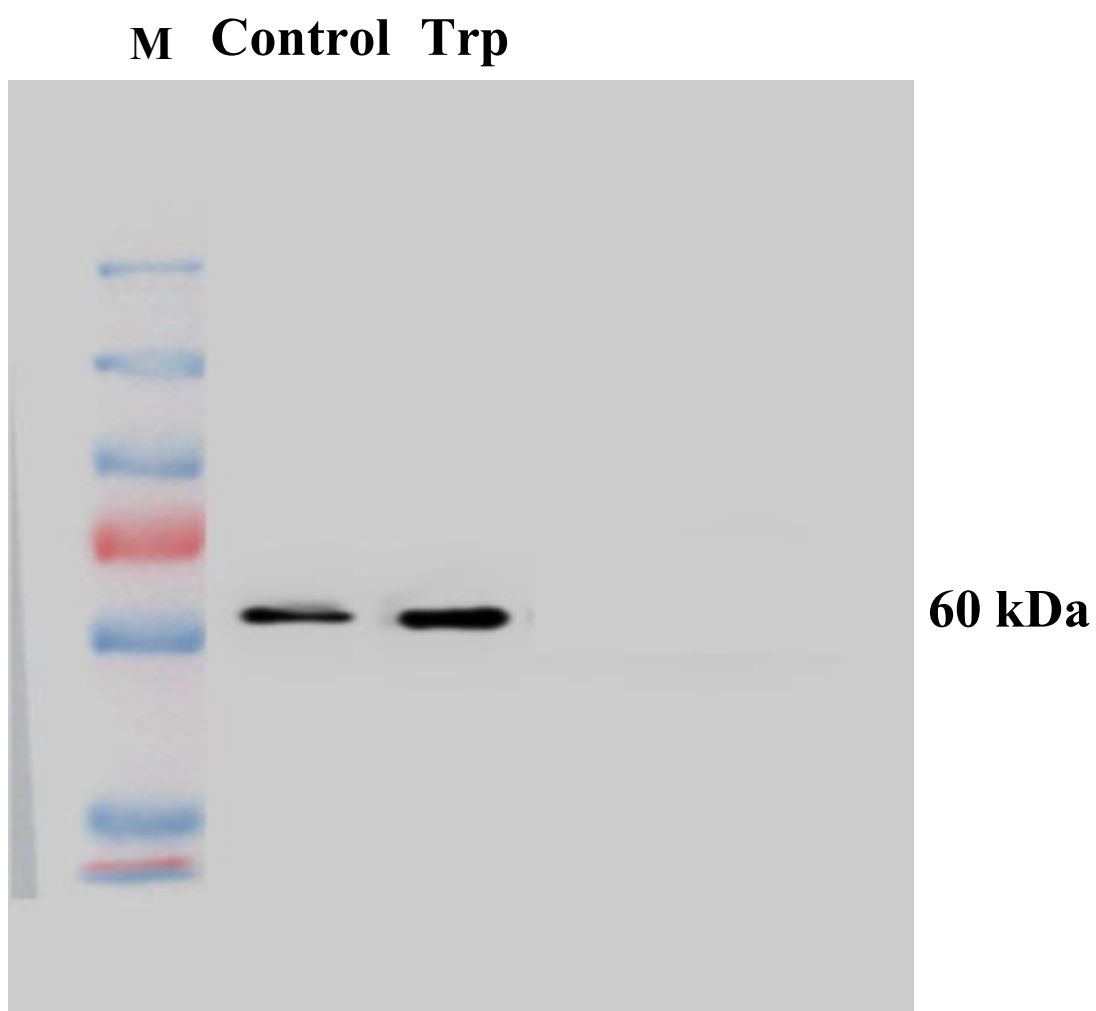


**Replicate 2**

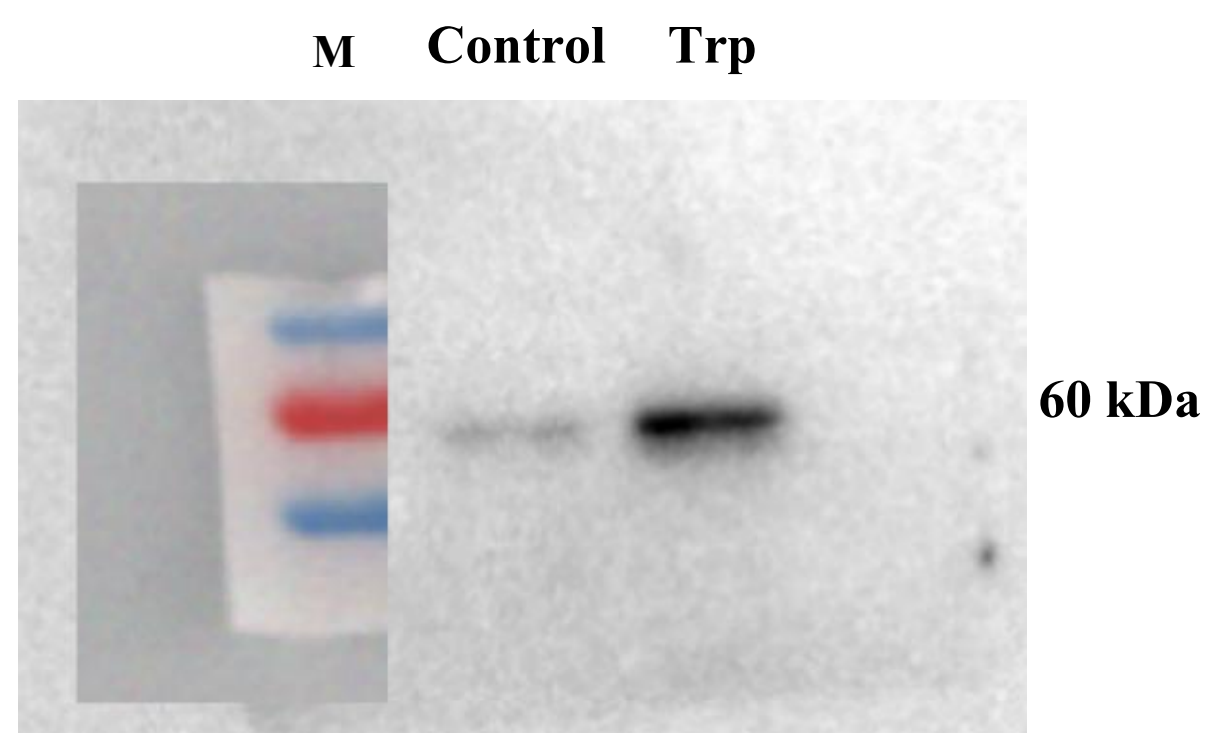


**Replicate 3**

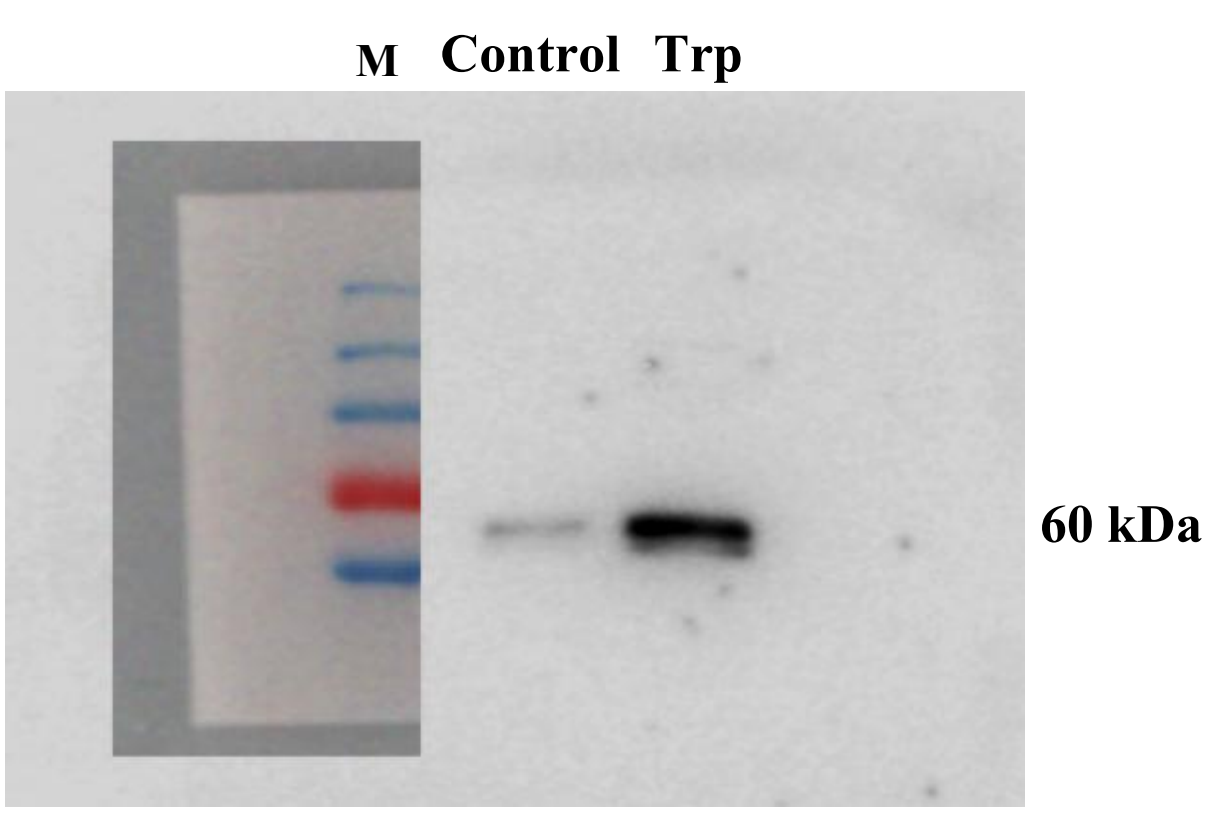


**Replicate 4**

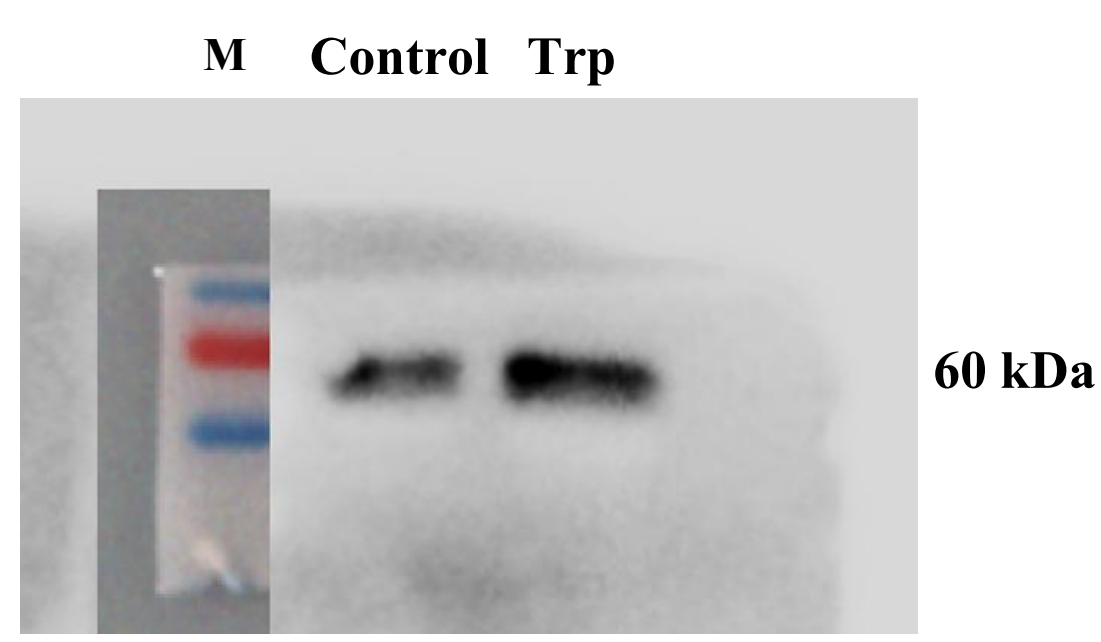

**Fig. 3a. p-4E-BP1. Original uncropped chemiluminescence images.**
The loading order and identity of experimental samples are indicated above each image. The molecular weight marker lane (M) is indicated where applicable. The expected molecular weight of fibronectin is approximately 20 kDa. All images were captured using a ChemiDoc™ Touch Imaging System (Bio-Rad Laboratories, Hercules, CA, USA).

**Replicate 1**

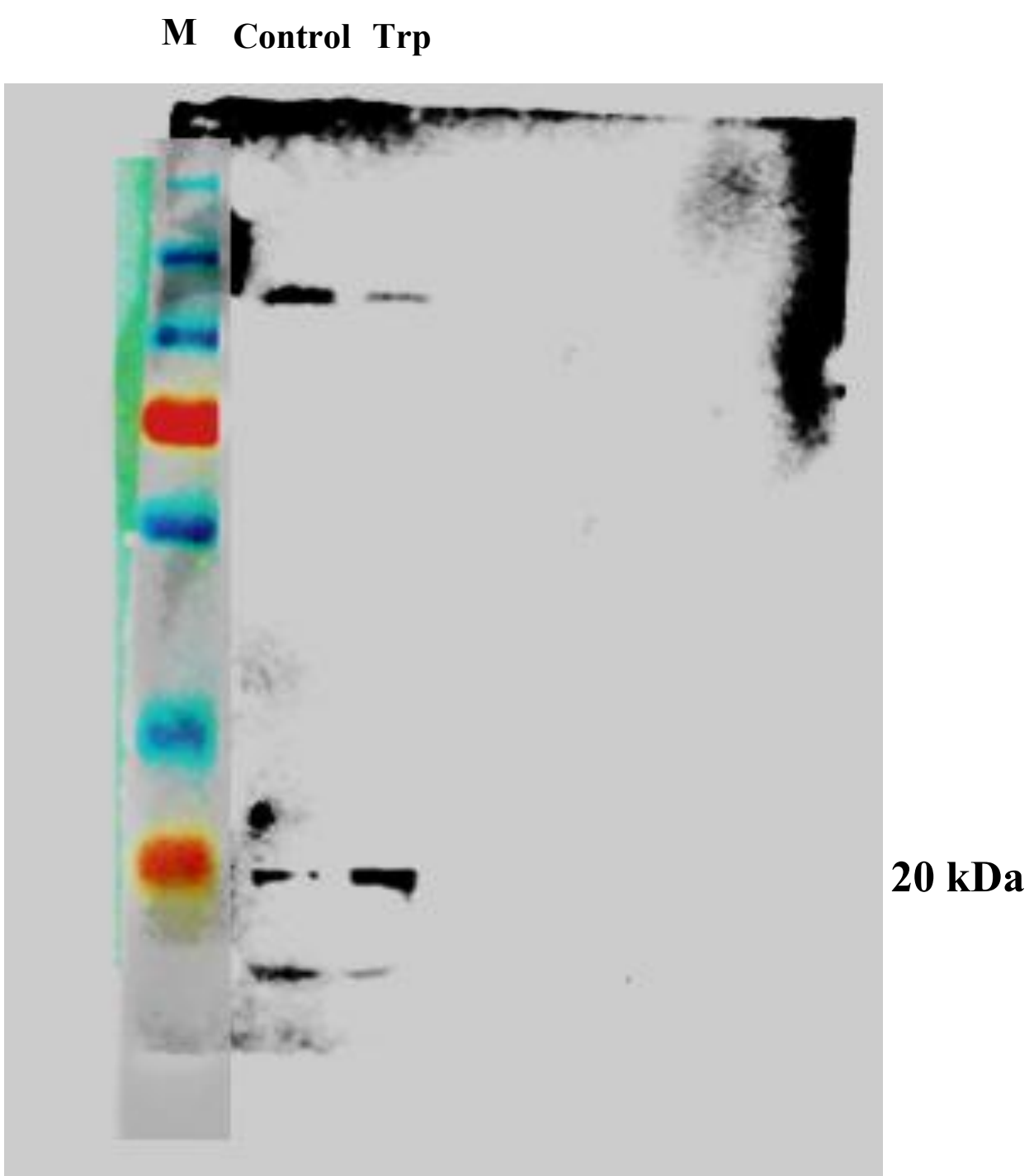


**Replicate 2**

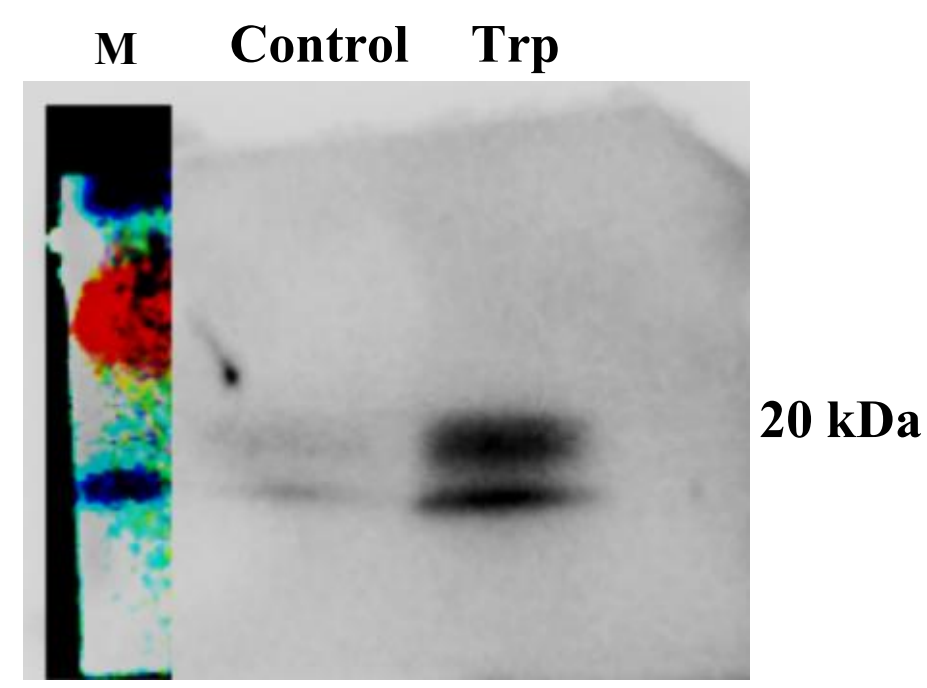


**Replicate 3**

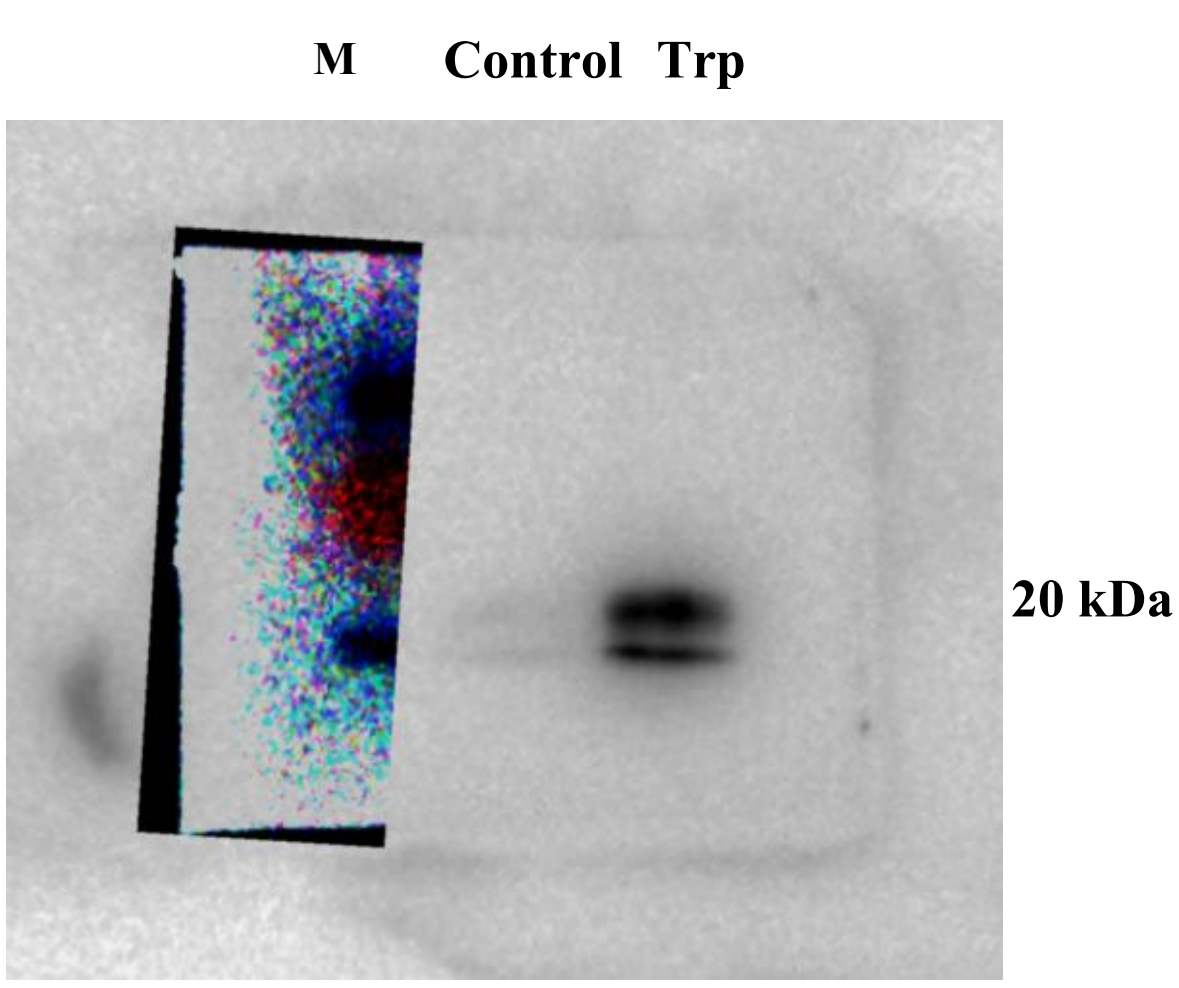


**Replicate 4**

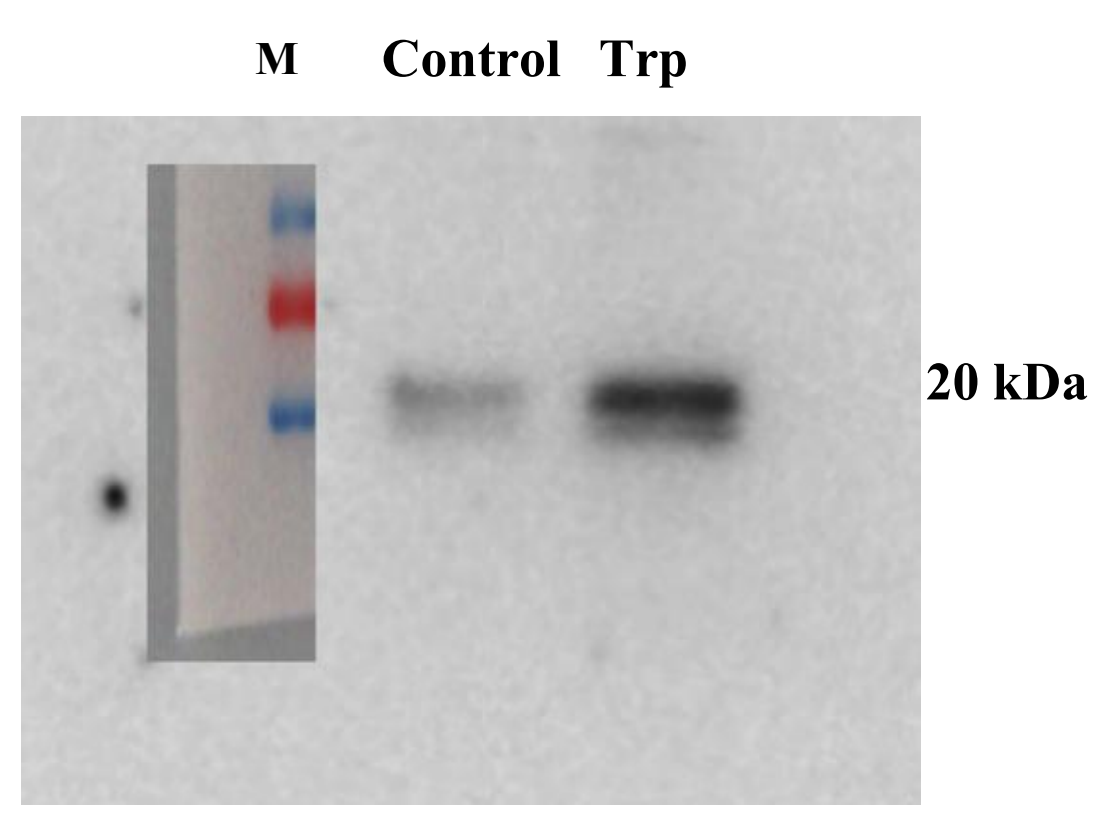

**Fig. 3a. p-S6. Original uncropped chemiluminescence images.**

The loading order and identity of experimental samples are indicated above each image. The molecular weight marker lane (M) is indicated where applicable. The expected molecular weight of fibronectin is approximately 30 kDa. All images were captured using a ChemiDoc™ Touch Imaging System (Bio-Rad Laboratories, Hercules, CA, USA).

**Replicate 1**

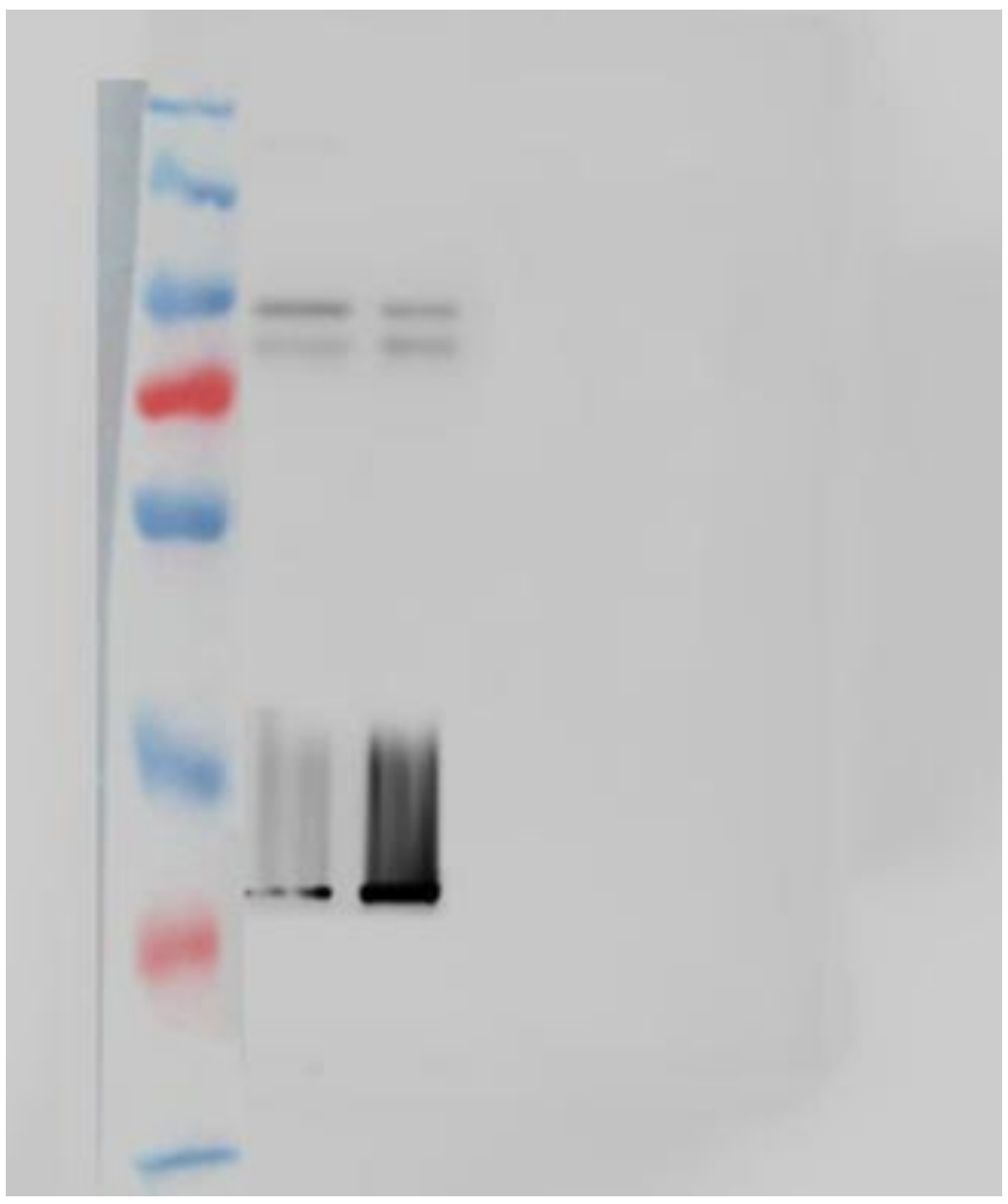


**Replicate 2**

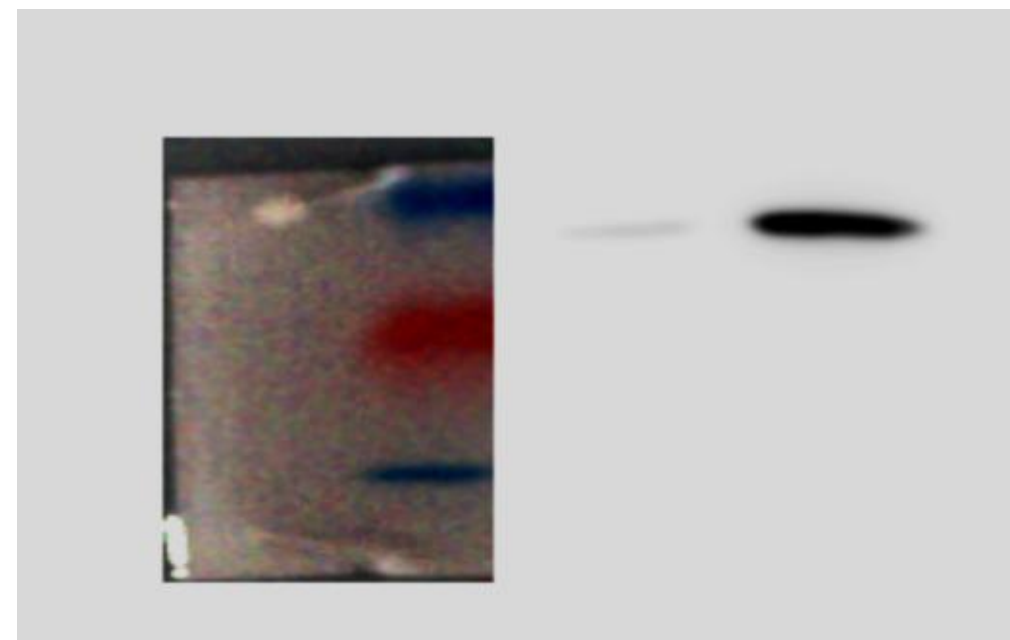


**Replicate 3**

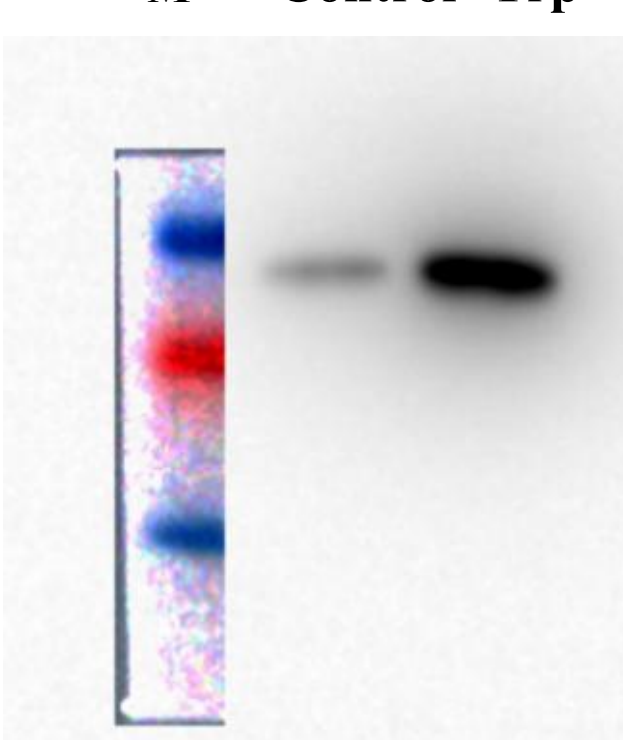


**Replicate 4**

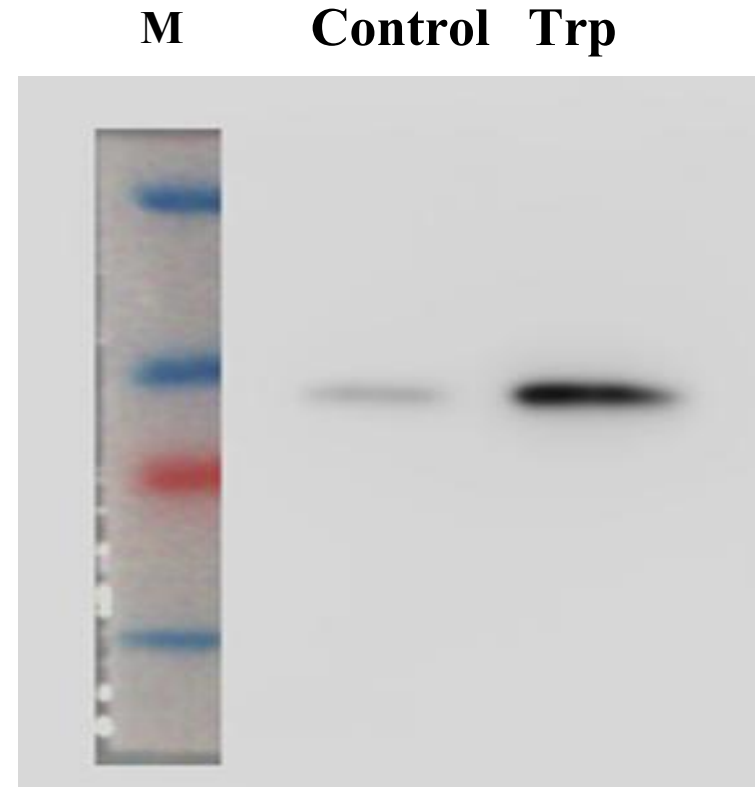

**Fig. 3a. p-mTOR. Original uncropped chemiluminescence images.**

The loading order and identity of experimental samples are indicated above each image. The molecular weight marker lane (M) is indicated where applicable. The expected molecular weight of fibronectin is approximately 280 kDa. All images were captured using a ChemiDoc™ Touch Imaging System (Bio-Rad Laboratories, Hercules, CA, USA).

**Replicate 1**

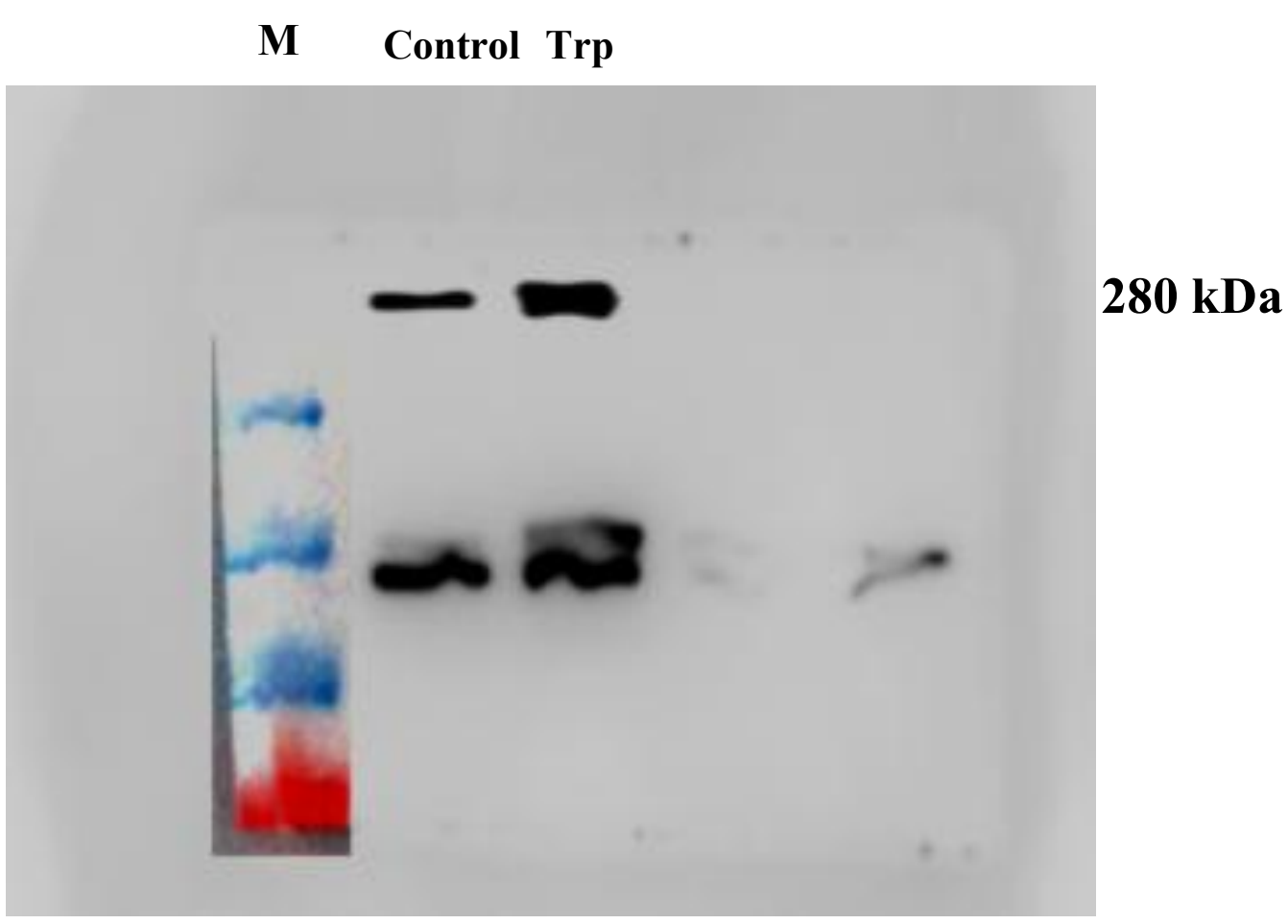


**Replicate 2**

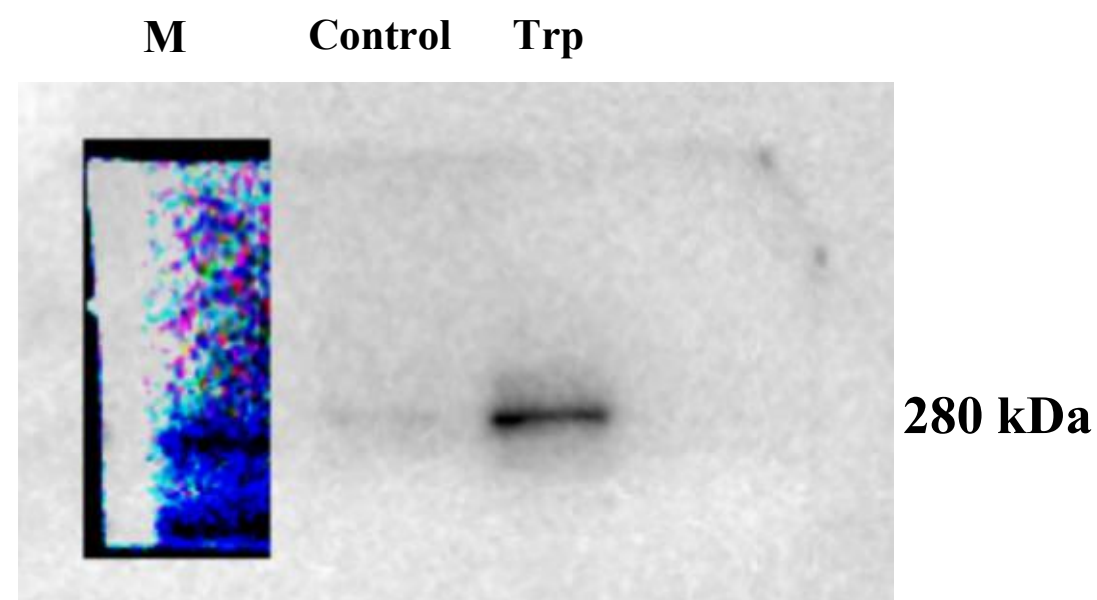


**Replicate 3**

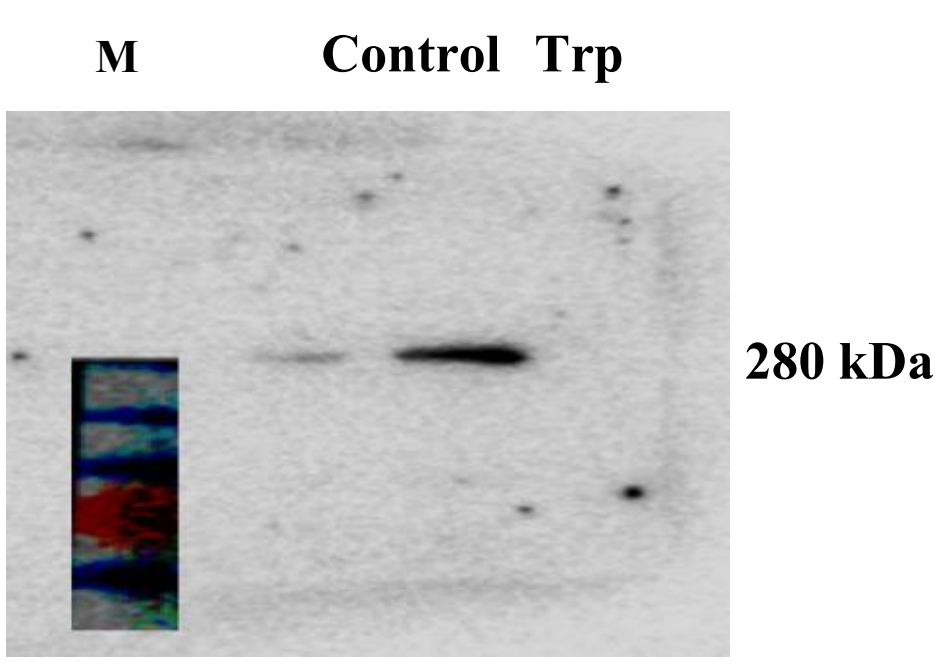


**Replicate 4**

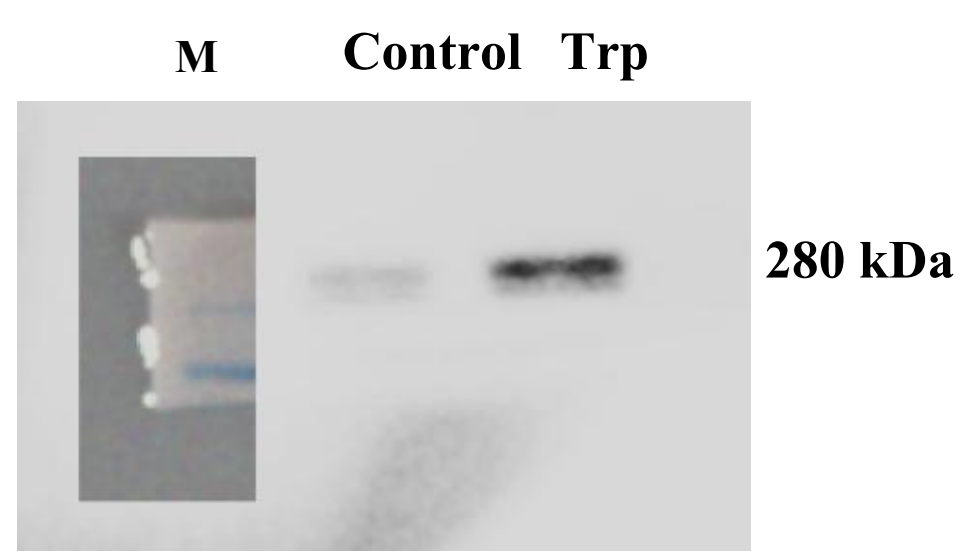

**Fig. 3a. β-actin. Original uncropped chemiluminescence images.**

The loading order and identity of experimental samples are indicated above each image. The molecular weight marker lane (M) is indicated where applicable. The expected molecular weight of fibronectin is approximately 45 kDa. All images were captured using a ChemiDoc™ Touch Imaging System (Bio-Rad Laboratories, Hercules, CA, USA).

**Replicate 1**

M Control Trp

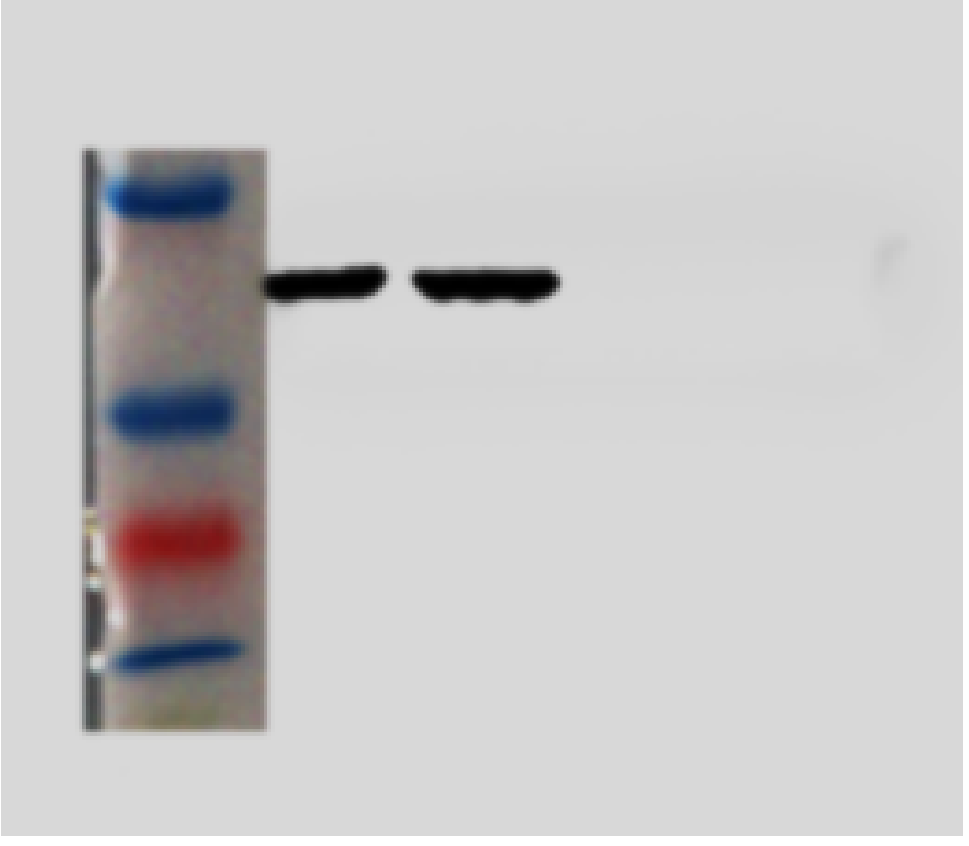

45 kDa

**Replicate 2**

M Control Trp

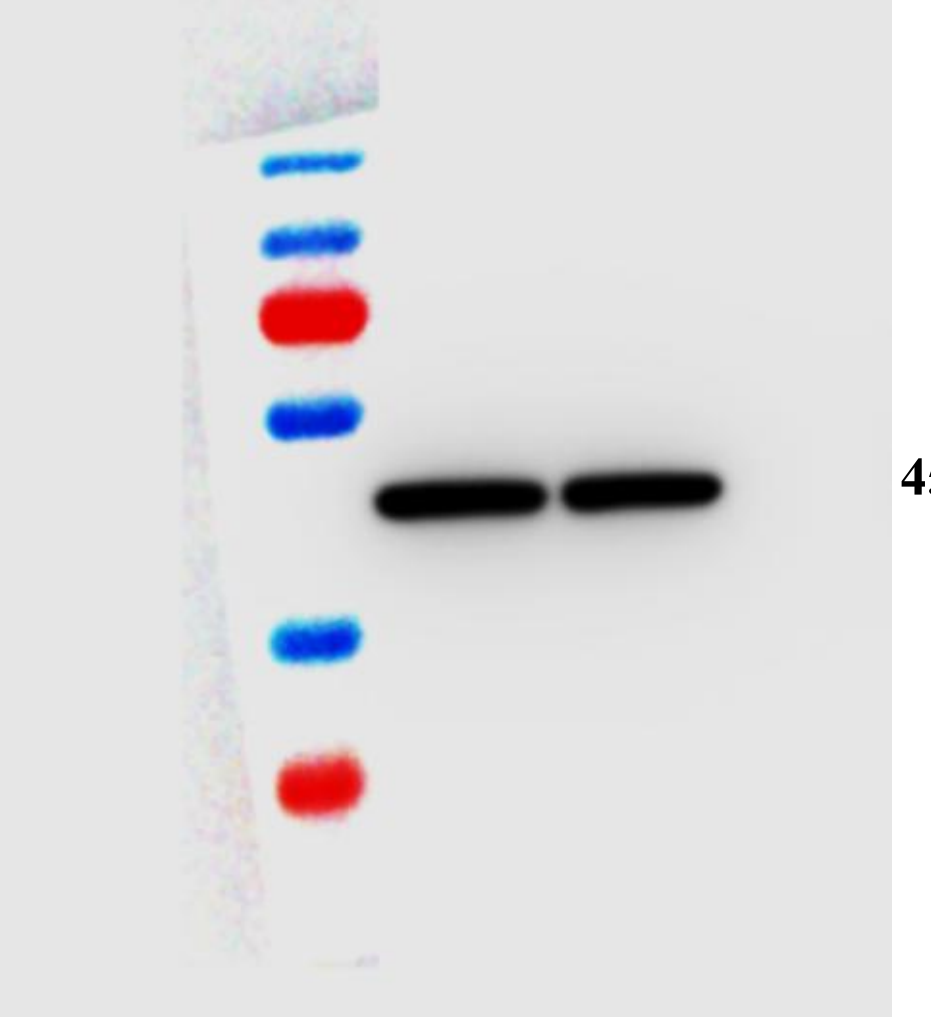

45 kDa

**Replicate 3**

M Control Trp

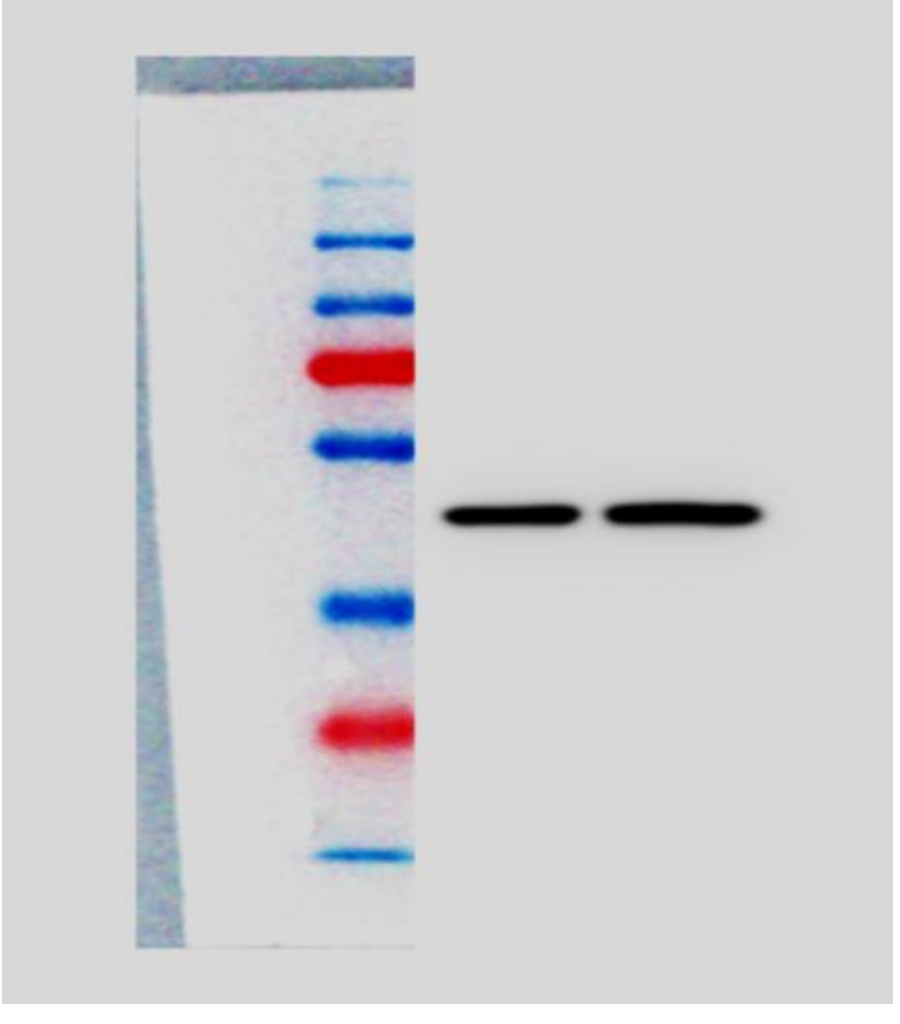

45 kDa

**Replicate 4**

M Control Trp

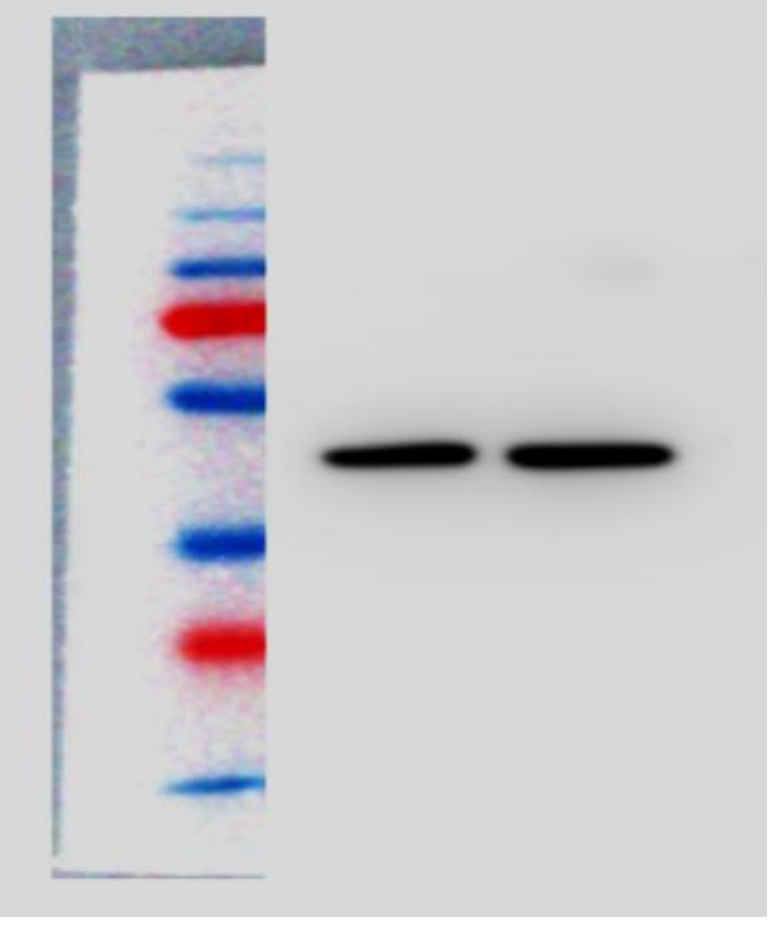

45 kDa

**Fig. 3b. p-SMAD3. Original uncropped chemiluminescence images.**

The loading order and identity of experimental samples are indicated above each image.The molecular weight marker lane (M) is indicated where applicable. The expected molecular weight of fibronectin is approximately 52 kDa. All images were captured using a ChemiDoc™ Touch Imaging System (Bio-Rad Laboratories, Hercules, CA, USA).

## Replicate 1

No treat | TGF-β

M Trp - CTL Trp + Trp - CTL Trp +

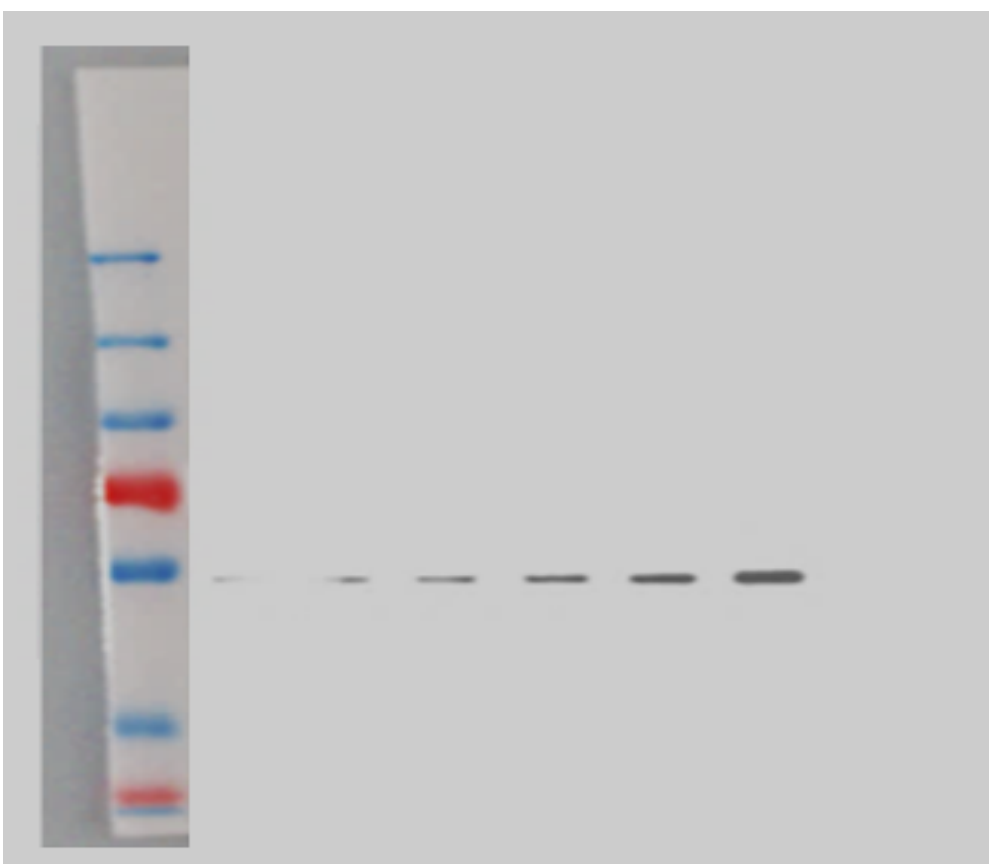

52 kDa

## Replicate 2

No treat | TGF-β

M Trp - CTL Trp + Trp - CTL Trp +

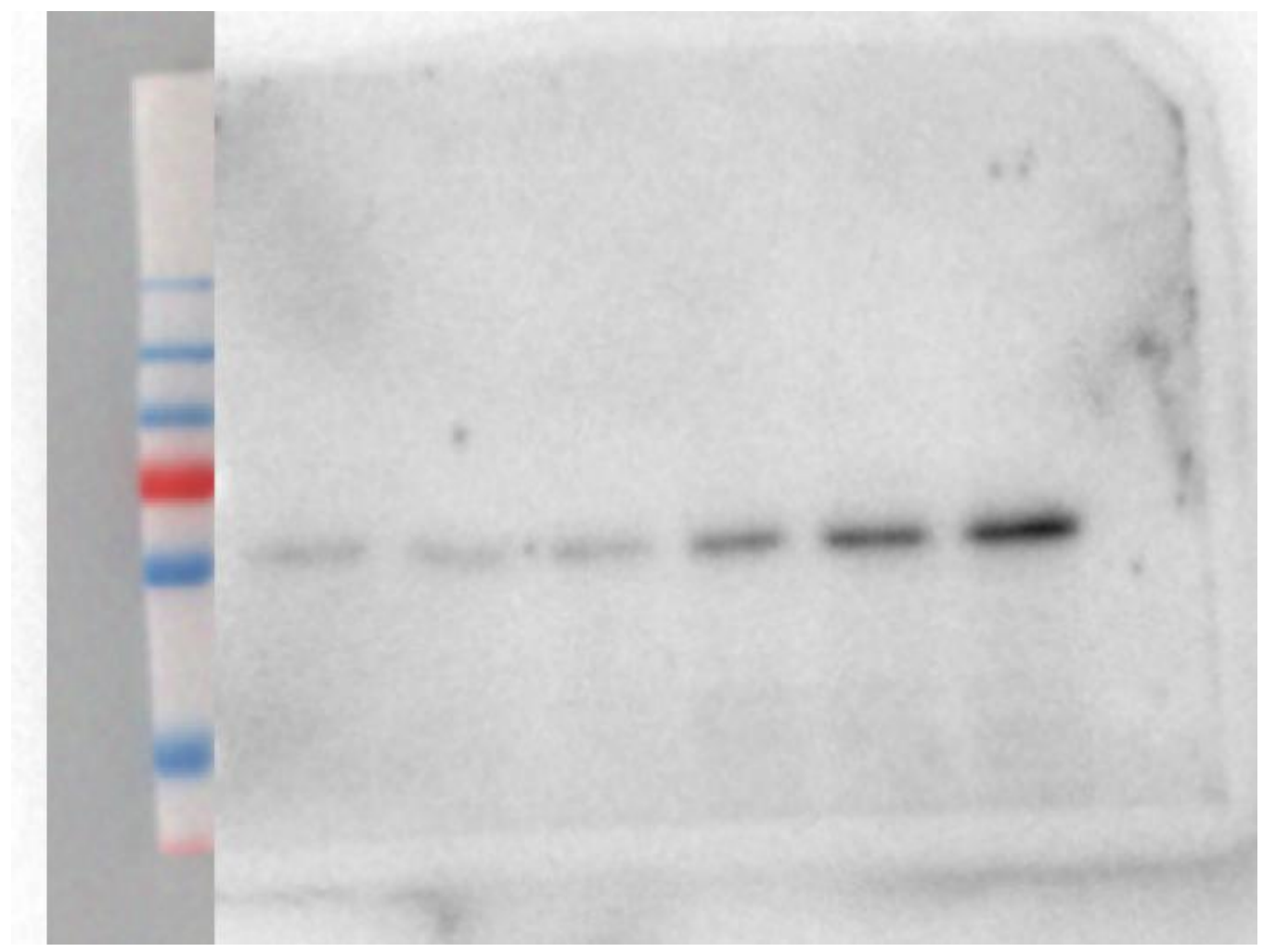

52 kDa

## Replicate 3

No treat | TGF-β

M Trp - CTL Trp + Trp - CTL Trp +

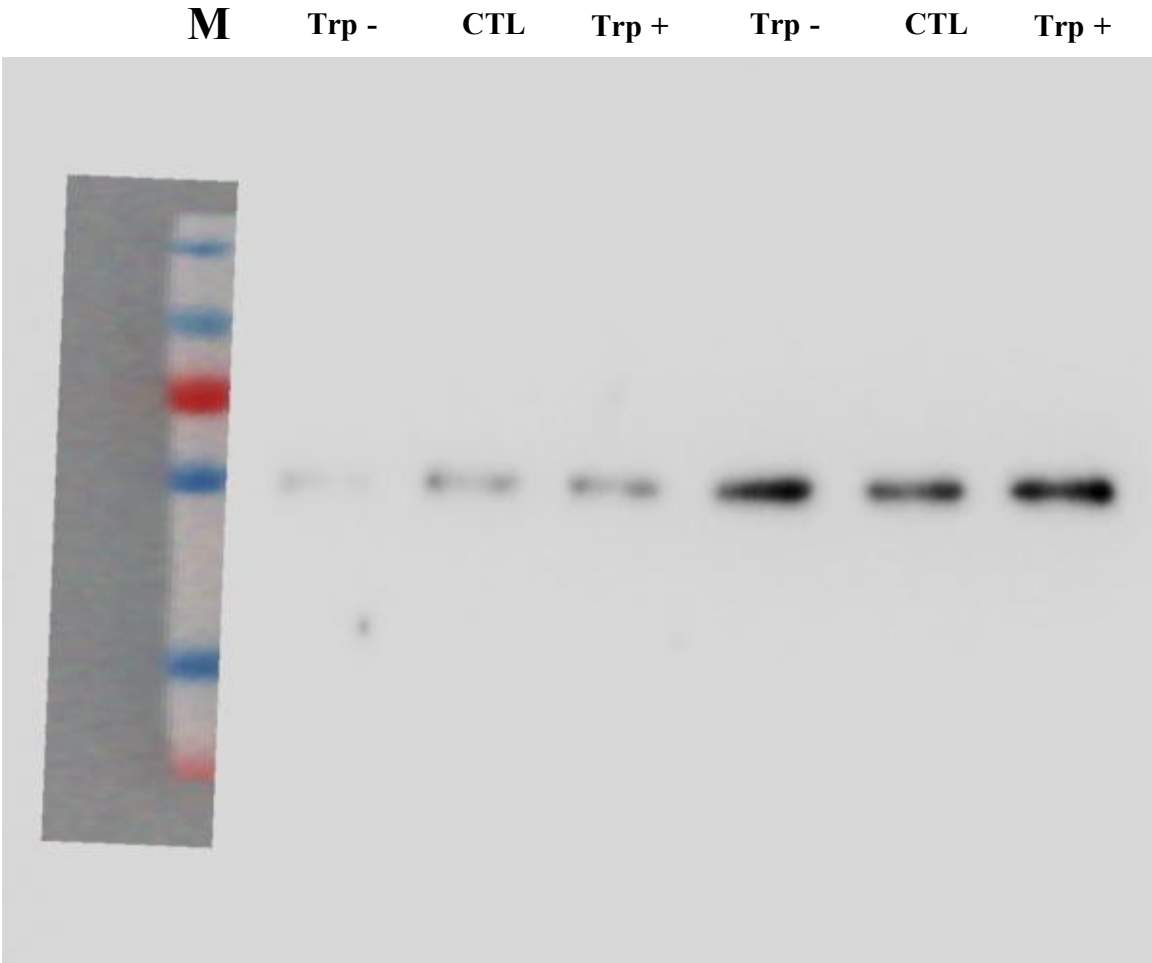

52 kDa

## Replicate 4

No treat | TGF-β

M Trp - CTL Trp + Trp - CTL Trp +

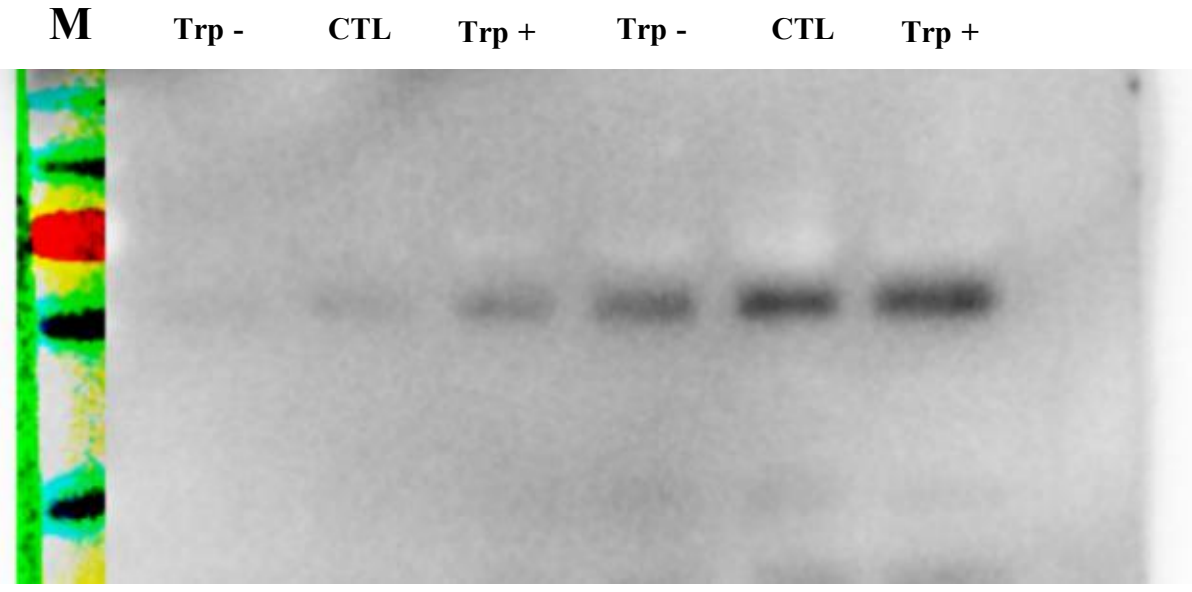

52 kDa

**Fig. 3b. SMAD3. Original uncropped chemiluminescence images.**

The loading order and identity of experimental samples are indicated above each image.The molecular weight marker lane (M) is indicated where applicable. The expected molecular weight of fibronectin is approximately 52 kDa. All images were captured using a ChemiDoc™ Touch Imaging System (Bio-Rad Laboratories, Hercules, CA, USA).

**Replicate 1**

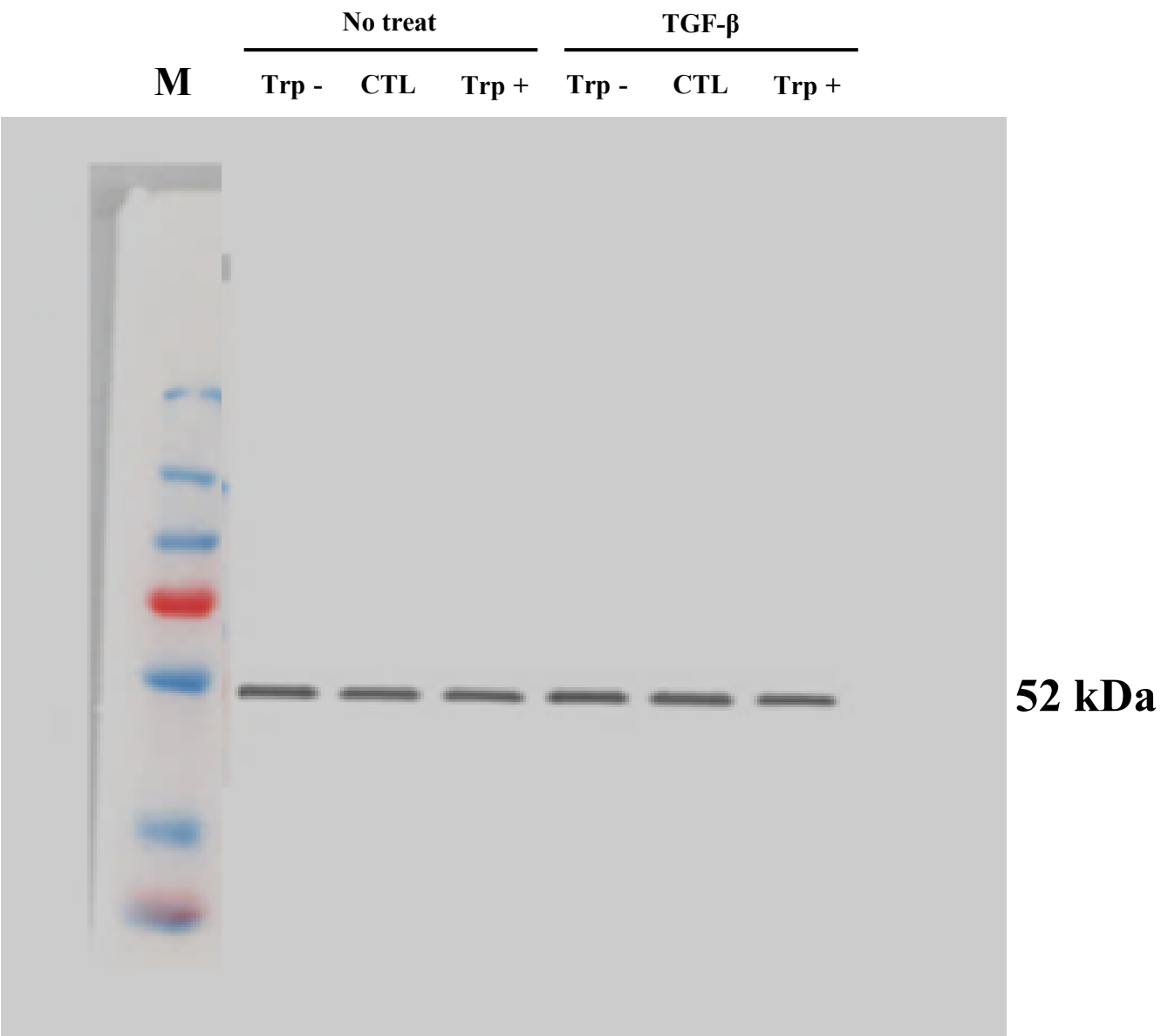


**Replicate 2**

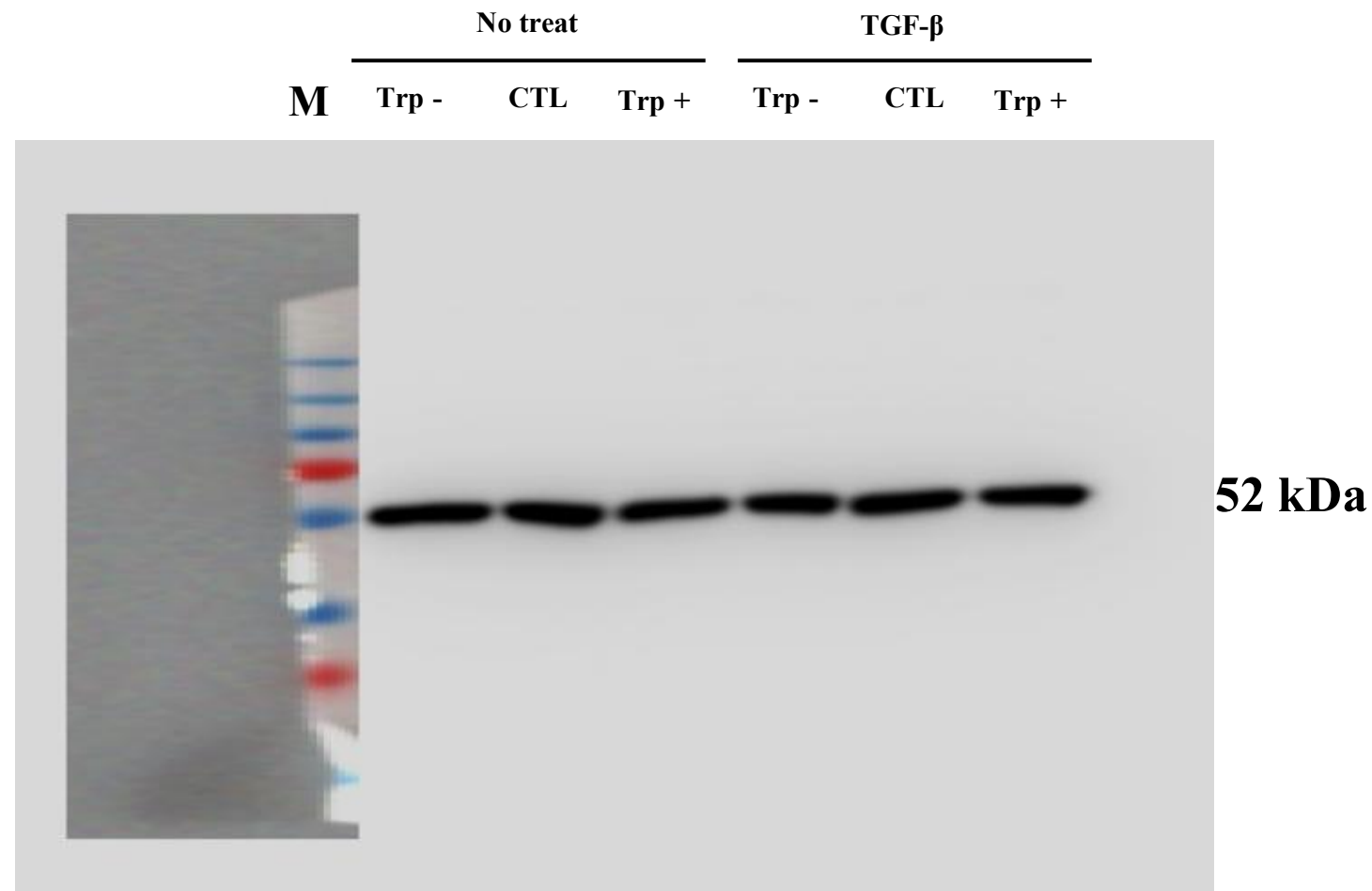


**Replicate 3**

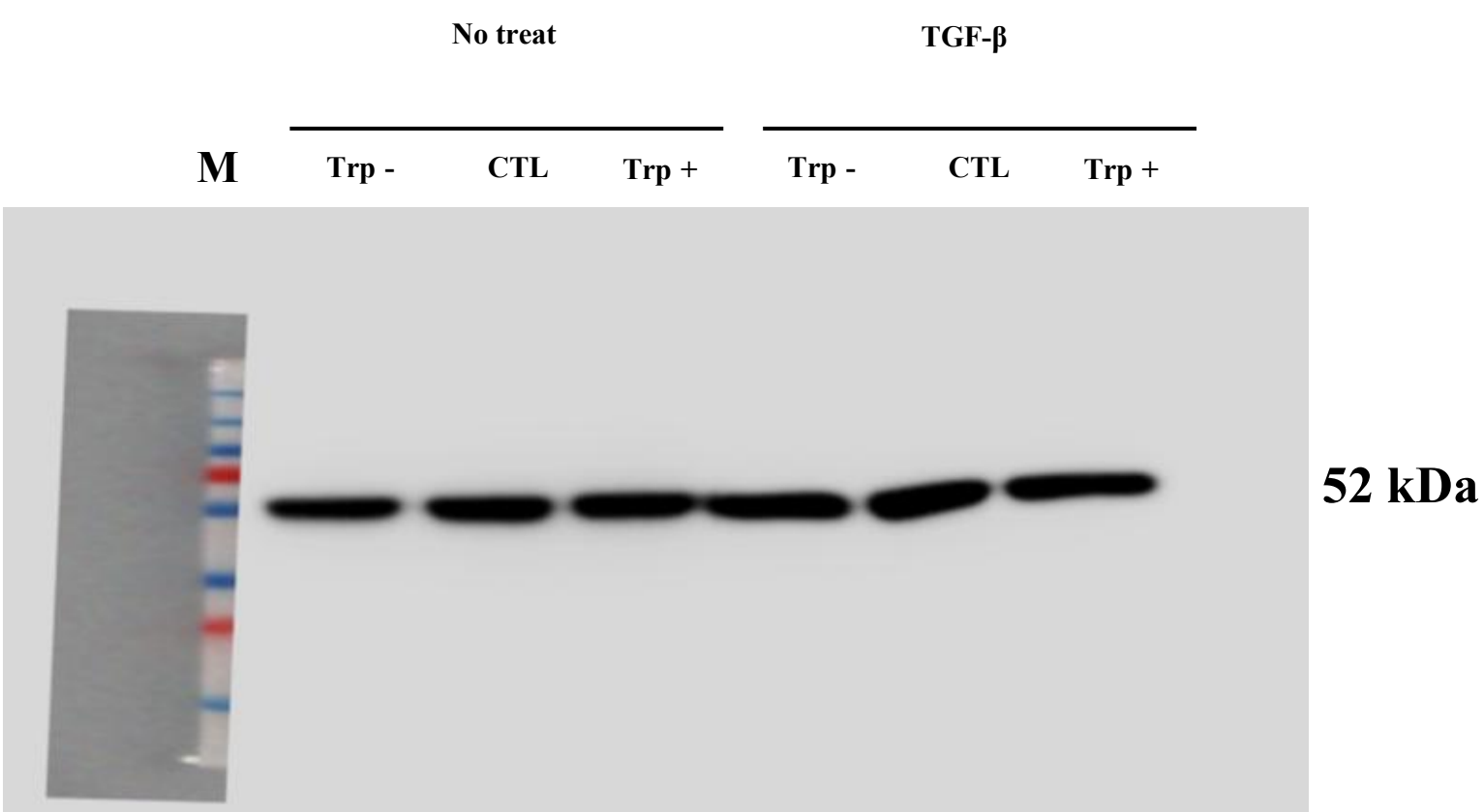


**Replicate 4**

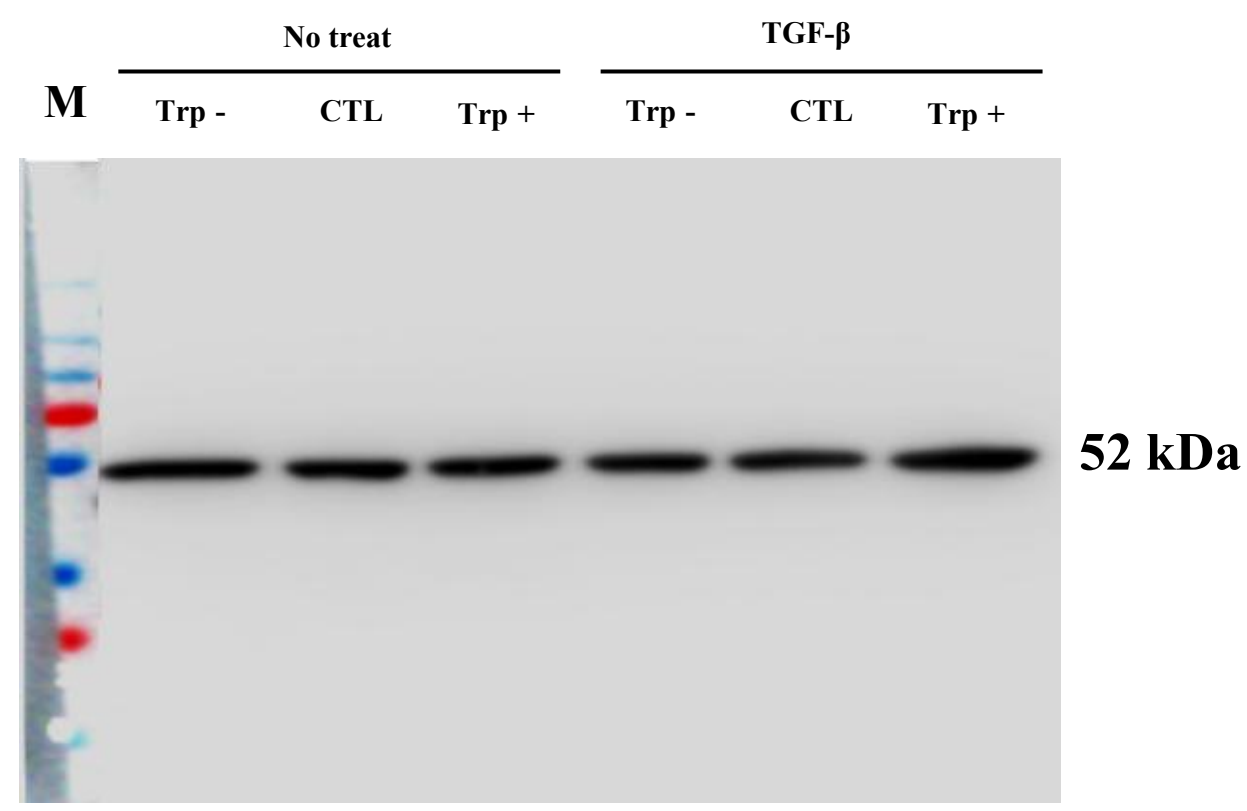

**Fig. 3b. p-SMAD2. Original uncropped chemiluminescence images.**

The loading order and identity of experimental samples are indicated above each image.The molecular weight marker lane (M) is indicated where applicable. The expected molecular weight of fibronectin is approximately 60 kDa. All images were captured using a ChemiDoc™ Touch Imaging System (Bio-Rad Laboratories, Hercules, CA, USA).

**Replicate 1**

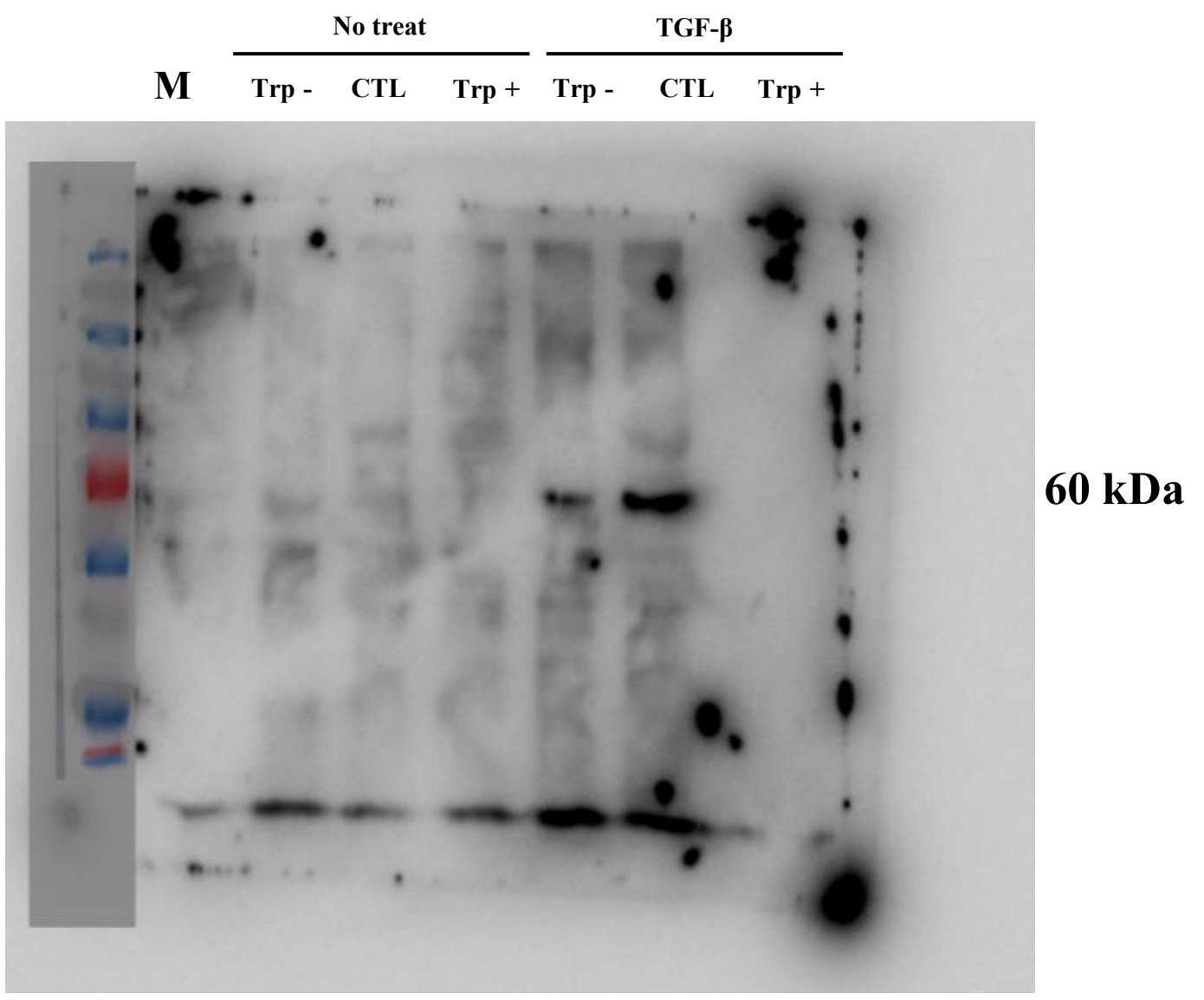


**Replicate 2**

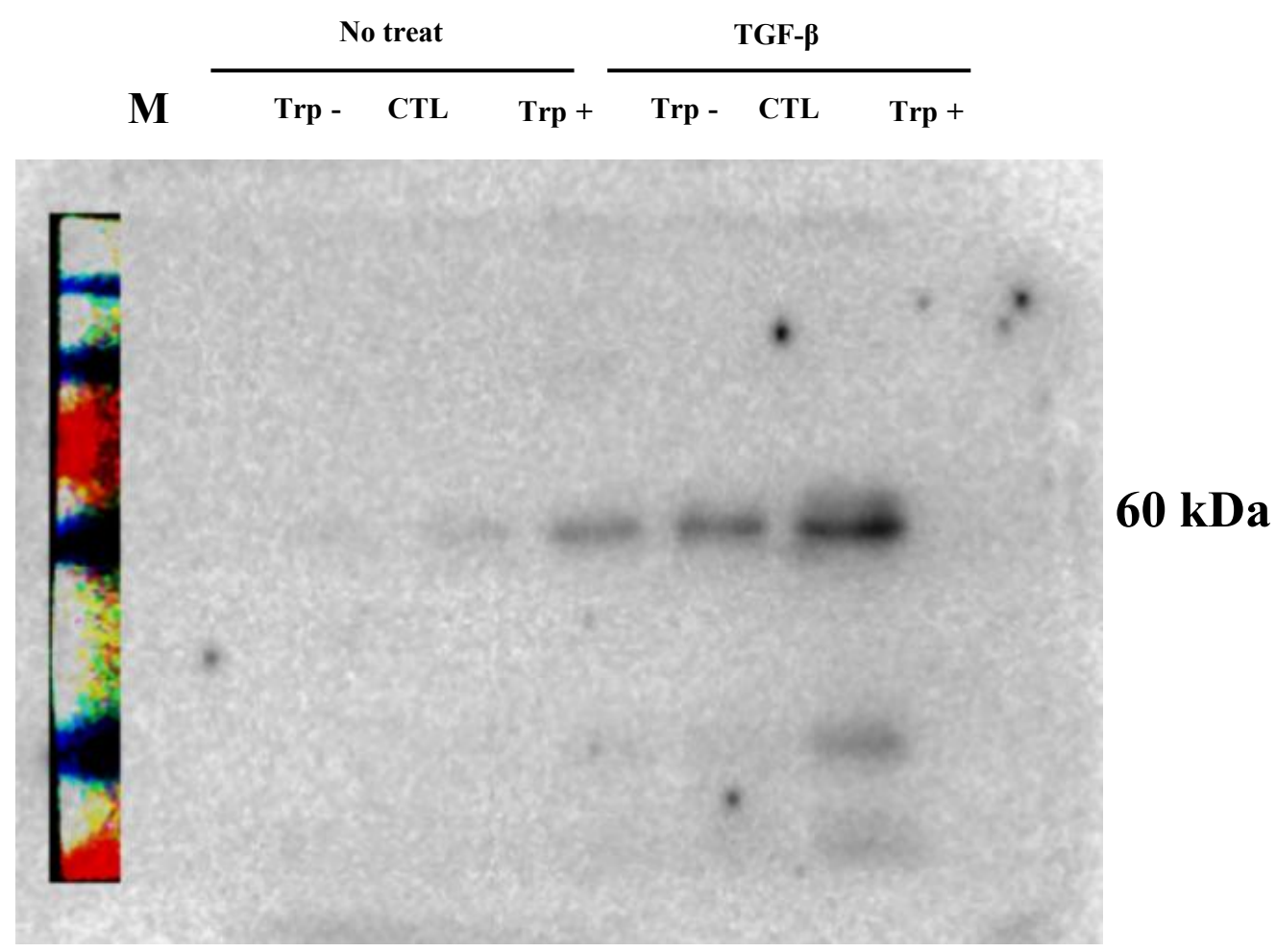


**Replicate 3**

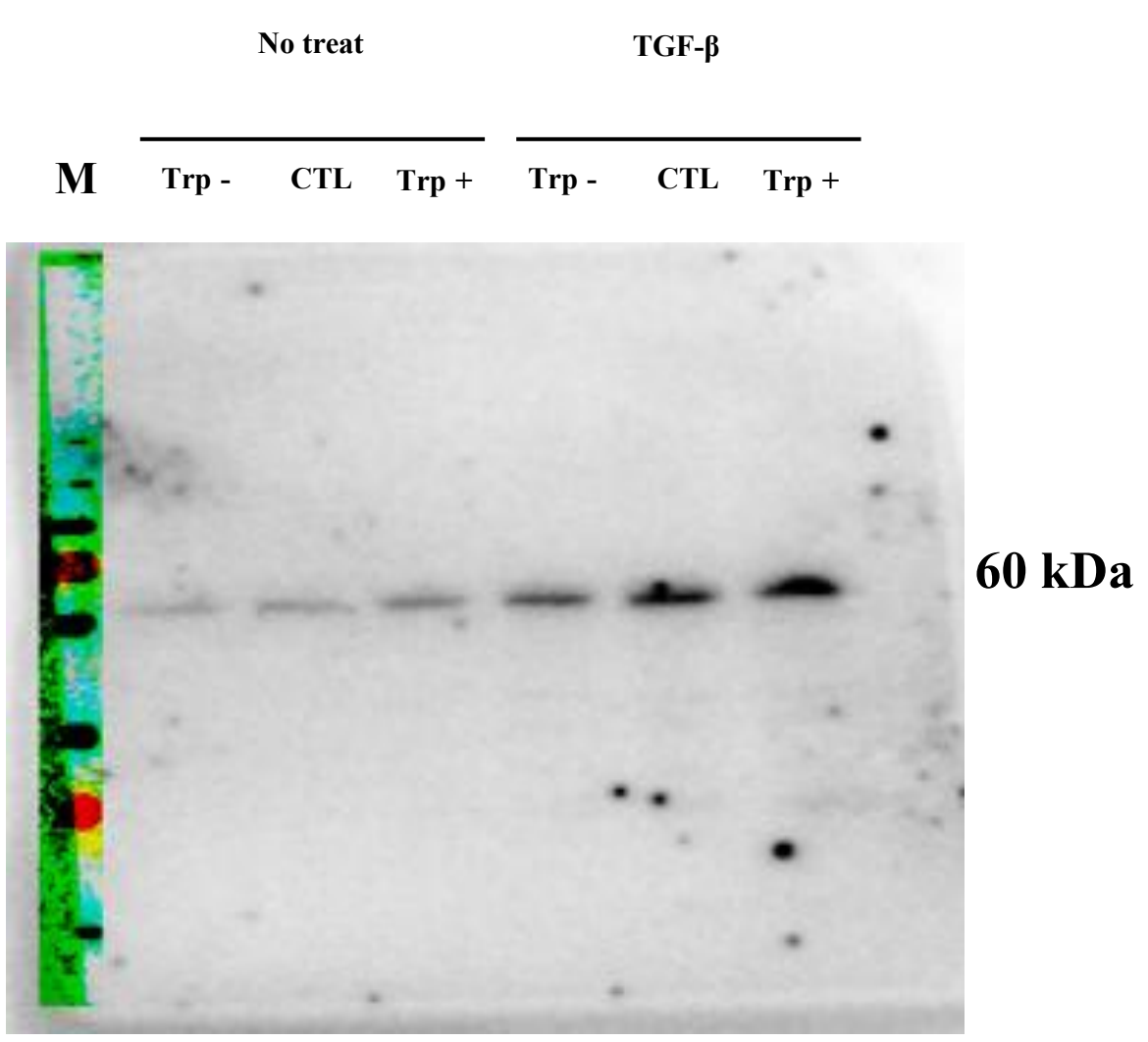


**Replicate 4**

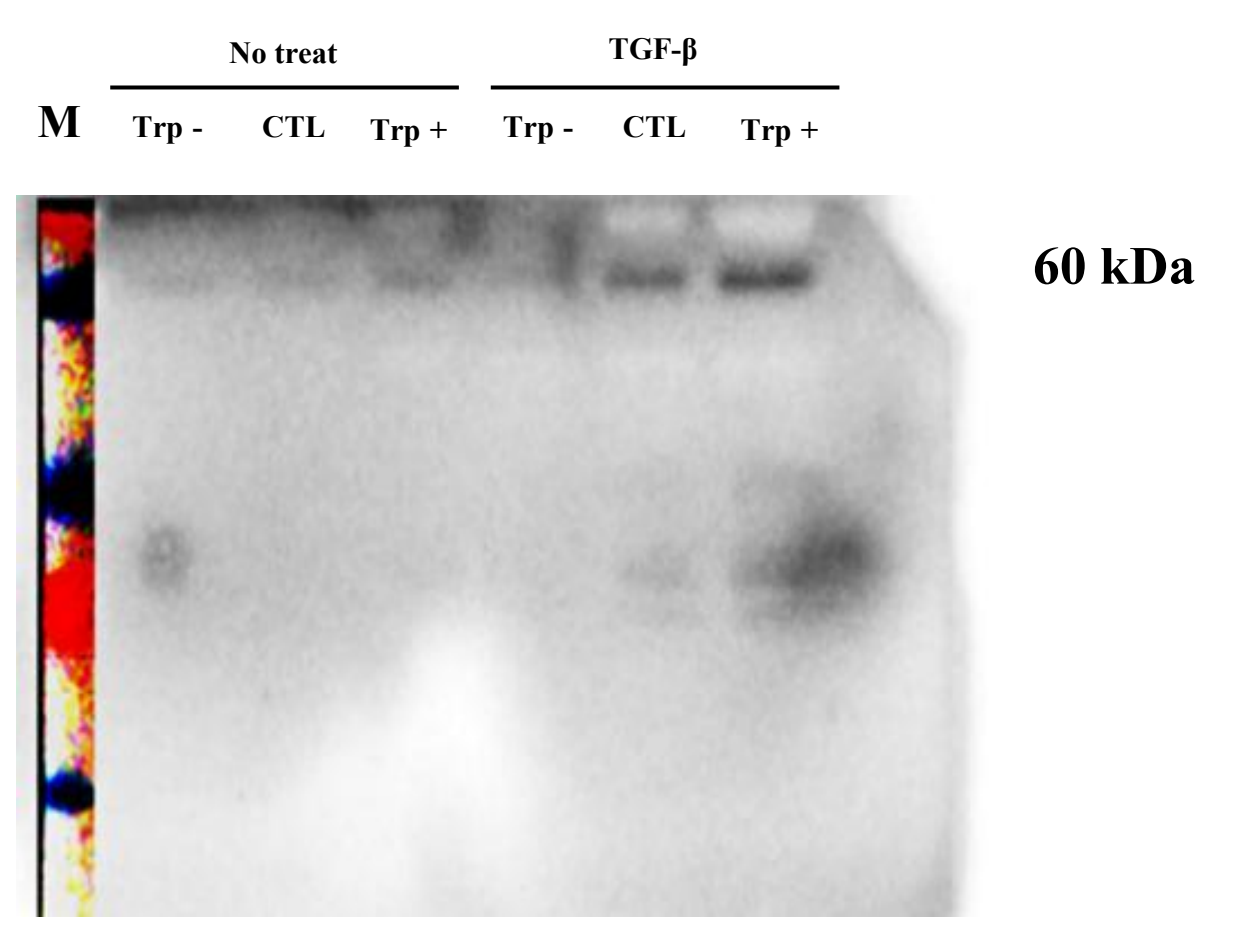

**Fig. 3b. SMAD2. Original uncropped chemiluminescence images.**

The loading order and identity of experimental samples are indicated above each image.The molecular weight marker lane (M) is indicated where applicable. The expected molecular weight of fibronectin is approximately 60 kDa. All images were captured using a ChemiDoc™ Touch Imaging System (Bio-Rad Laboratories, Hercules, CA, USA).

## Replicate 1

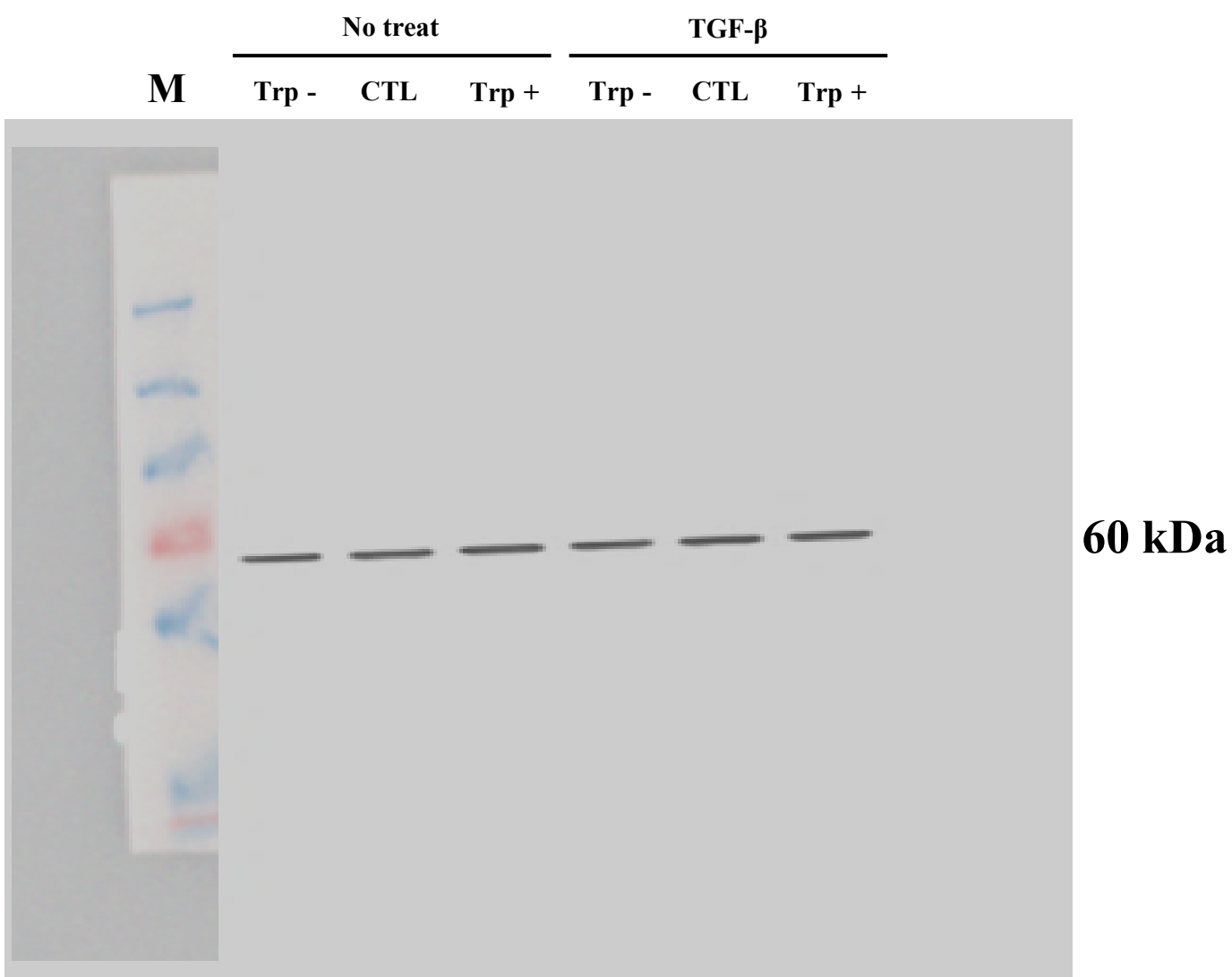


## Replicate 2

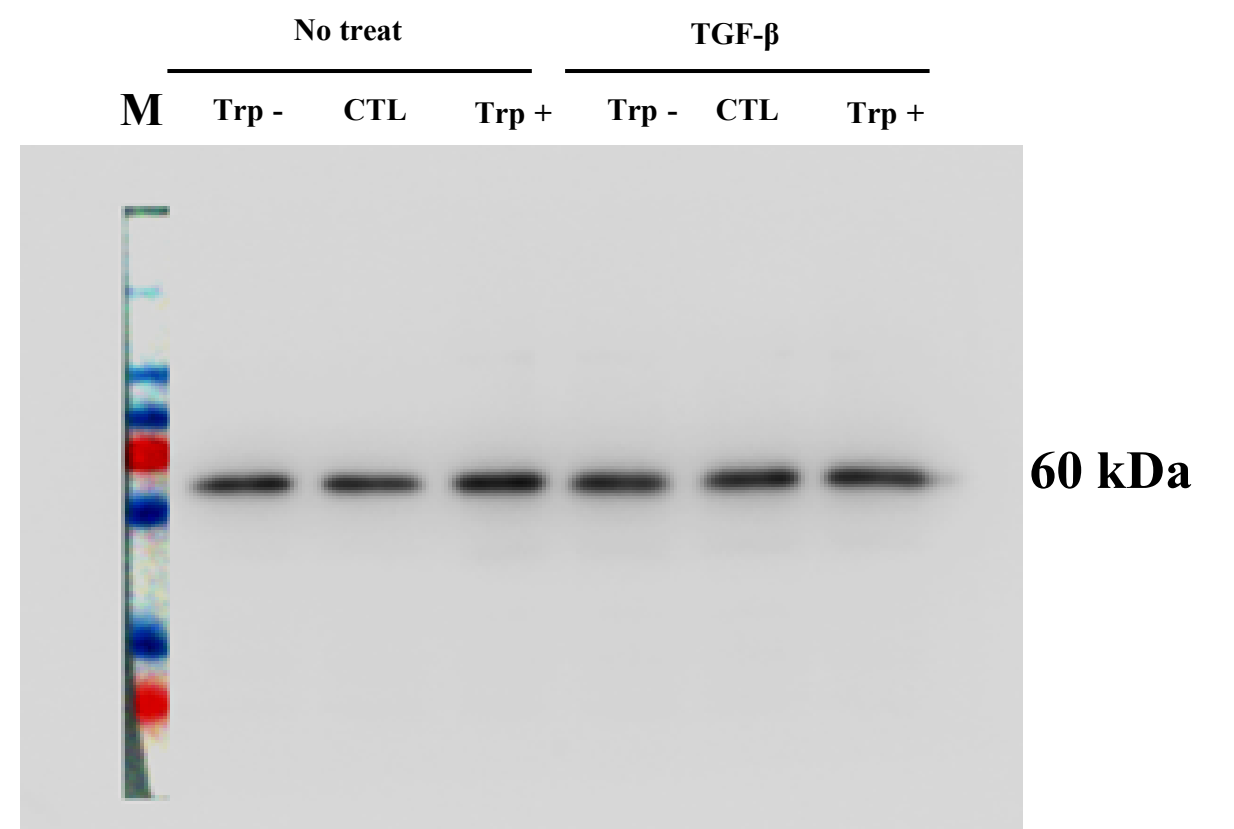


## Replicate 3

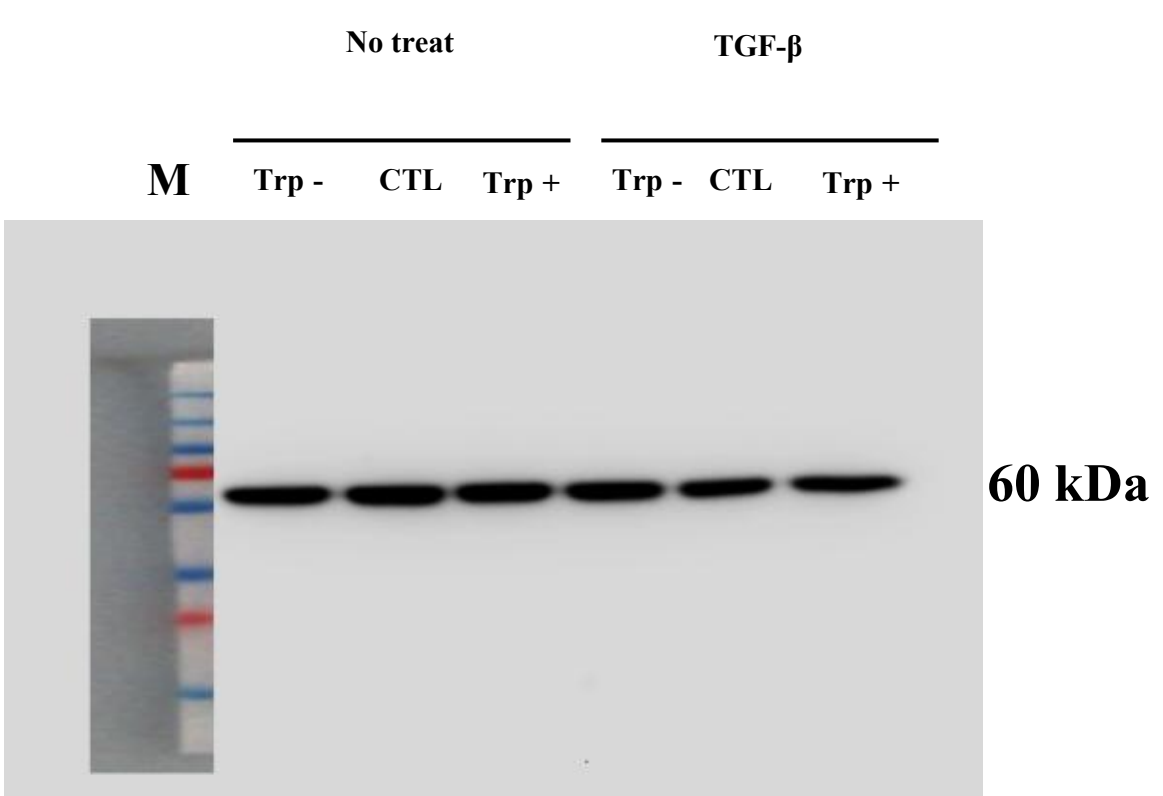


## Replicate 4

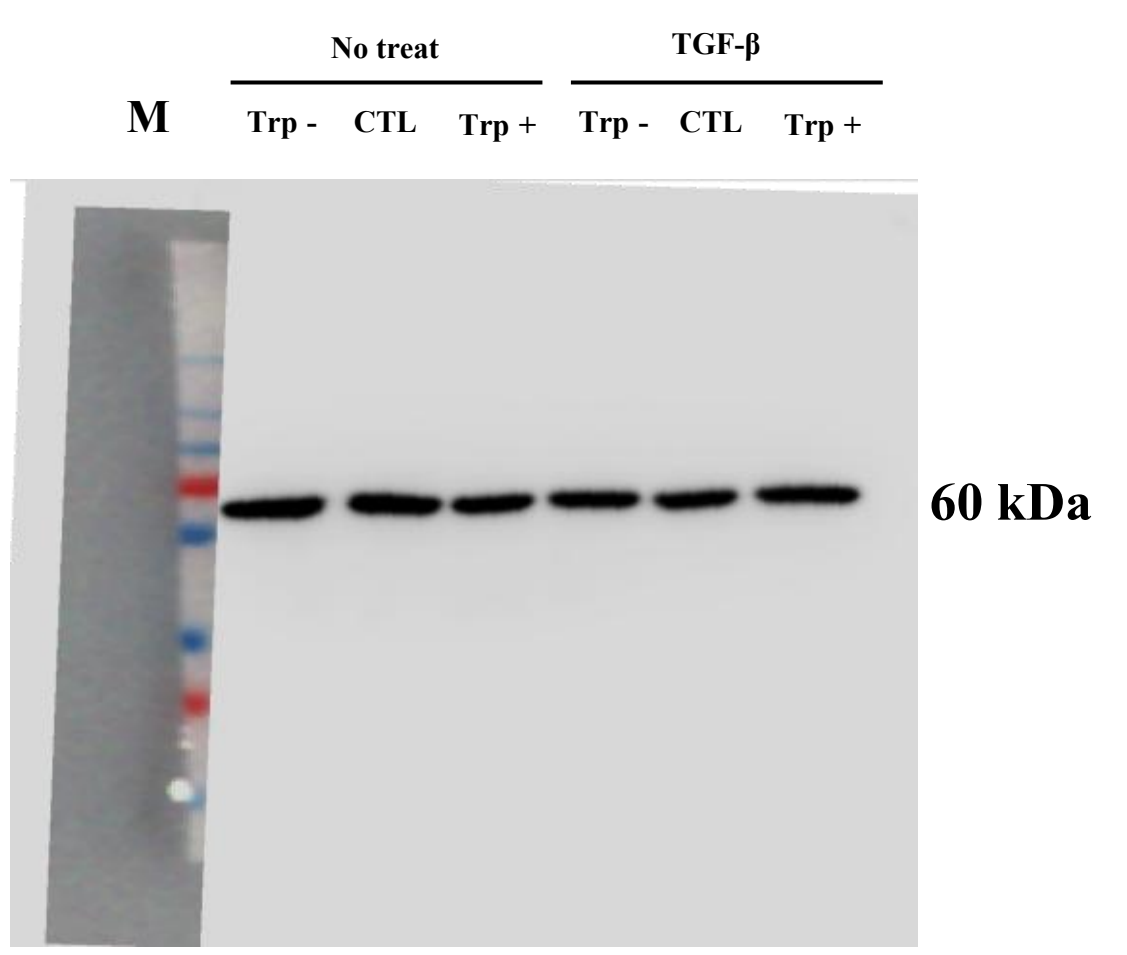

**Fig. 3b. β-actin. Original uncropped chemiluminescence images.**

The loading order and identity of experimental samples are indicated above each image.The molecular weight marker lane (M) is indicated where applicable. The expected molecular weight of fibronectin is approximately 45 kDa. All images were captured using a ChemiDoc™ Touch Imaging System (Bio-Rad Laboratories, Hercules, CA, USA).

**Replicate 1**

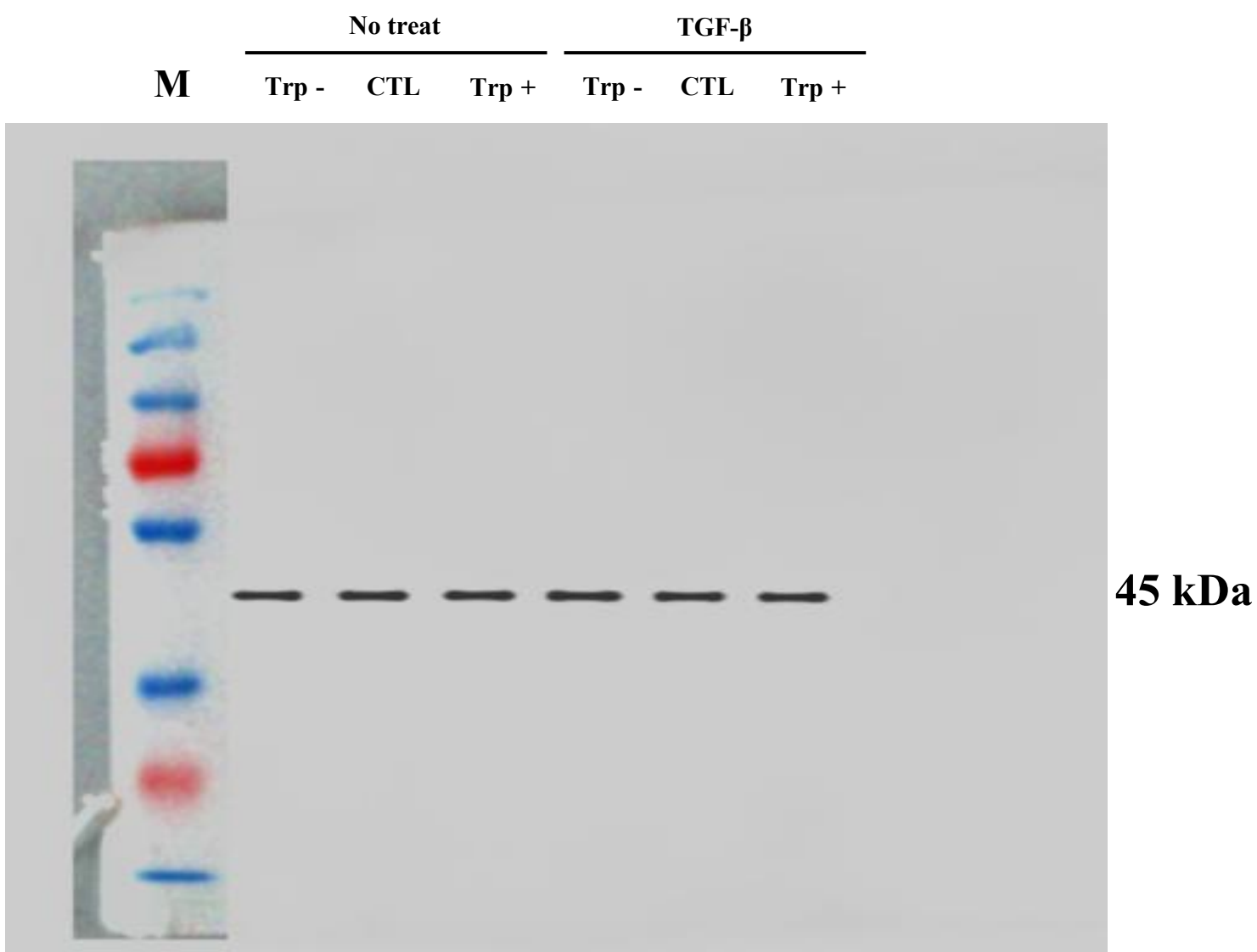


**Replicate 2**

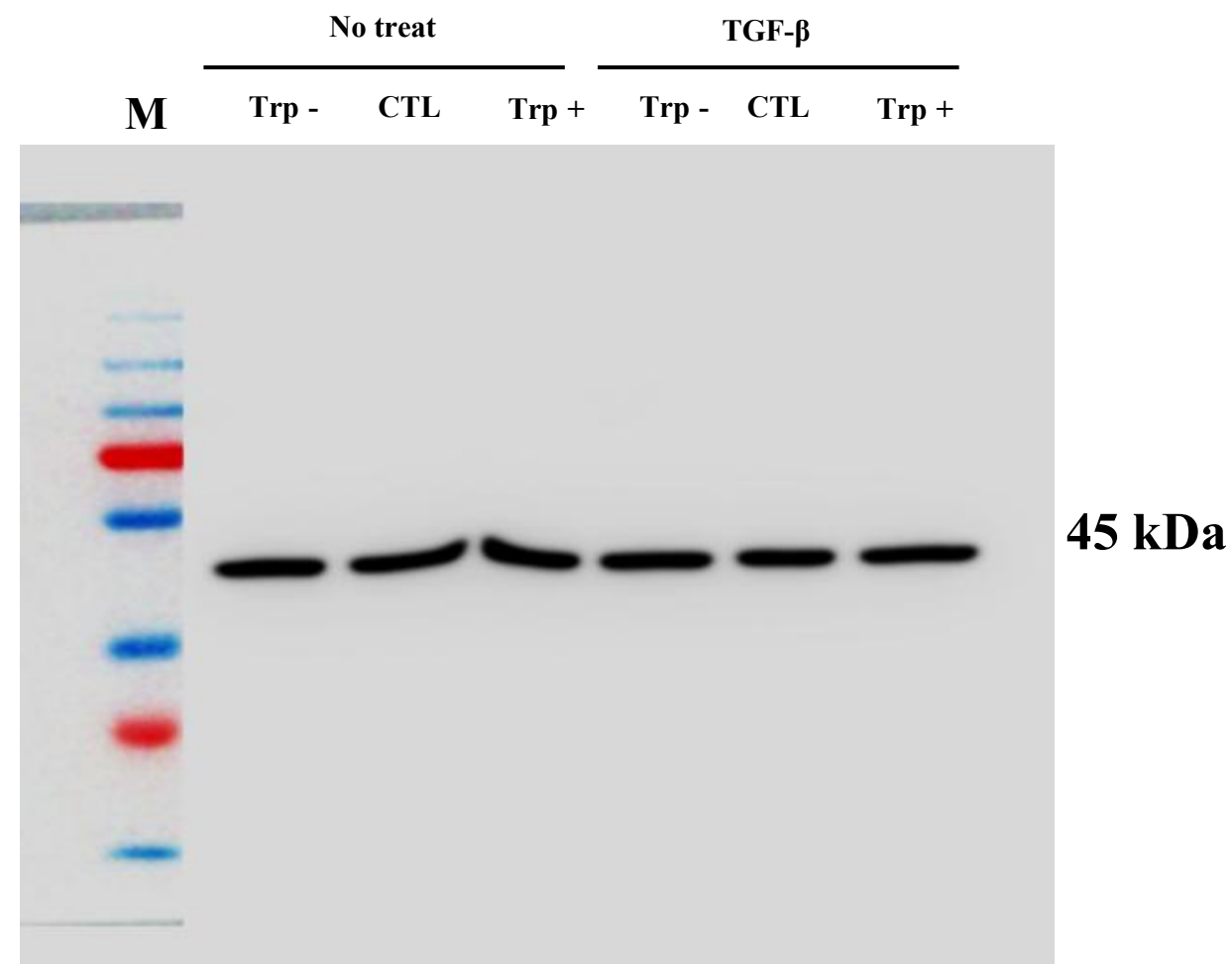


**Replicate 3**

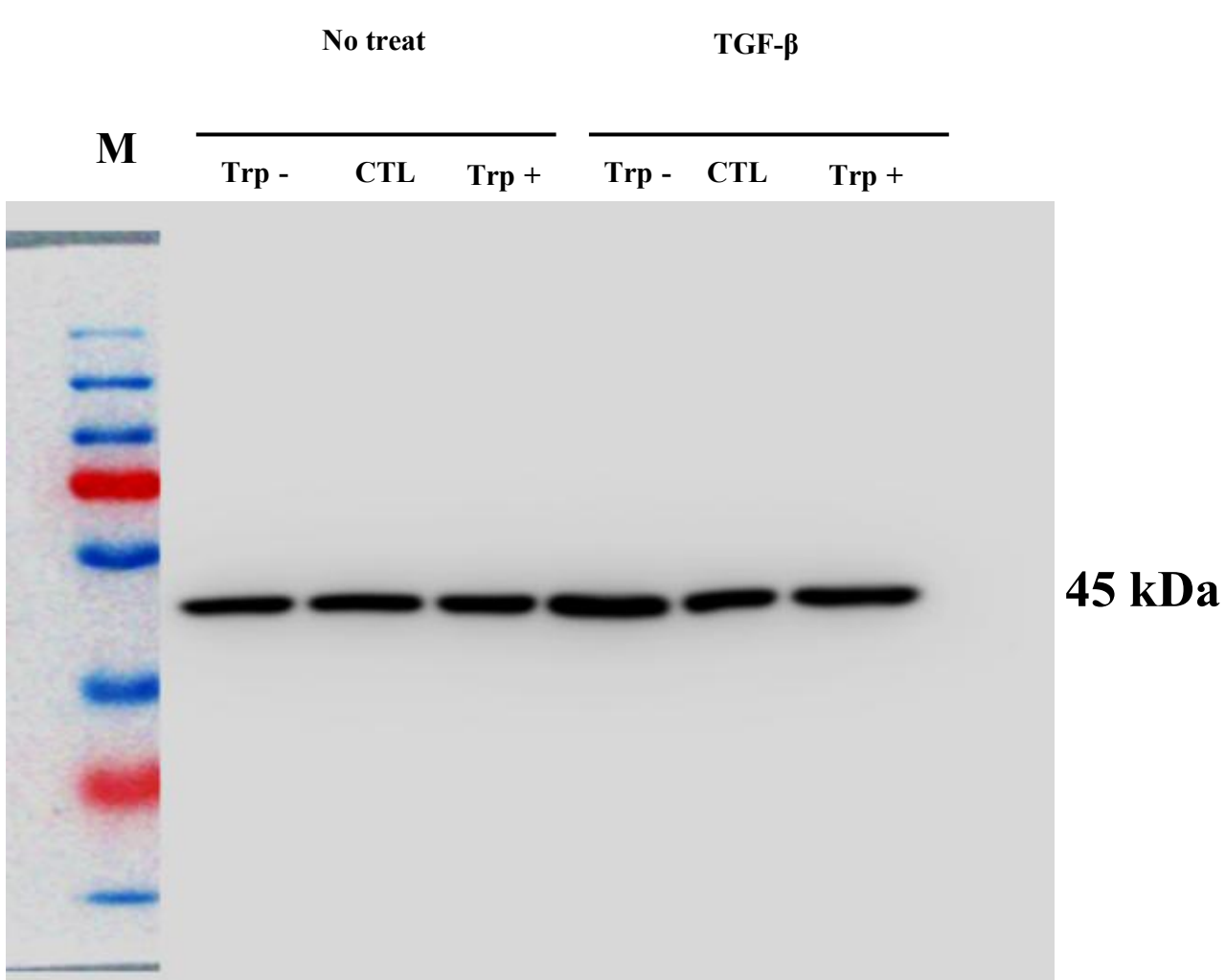


**Replicate 4**

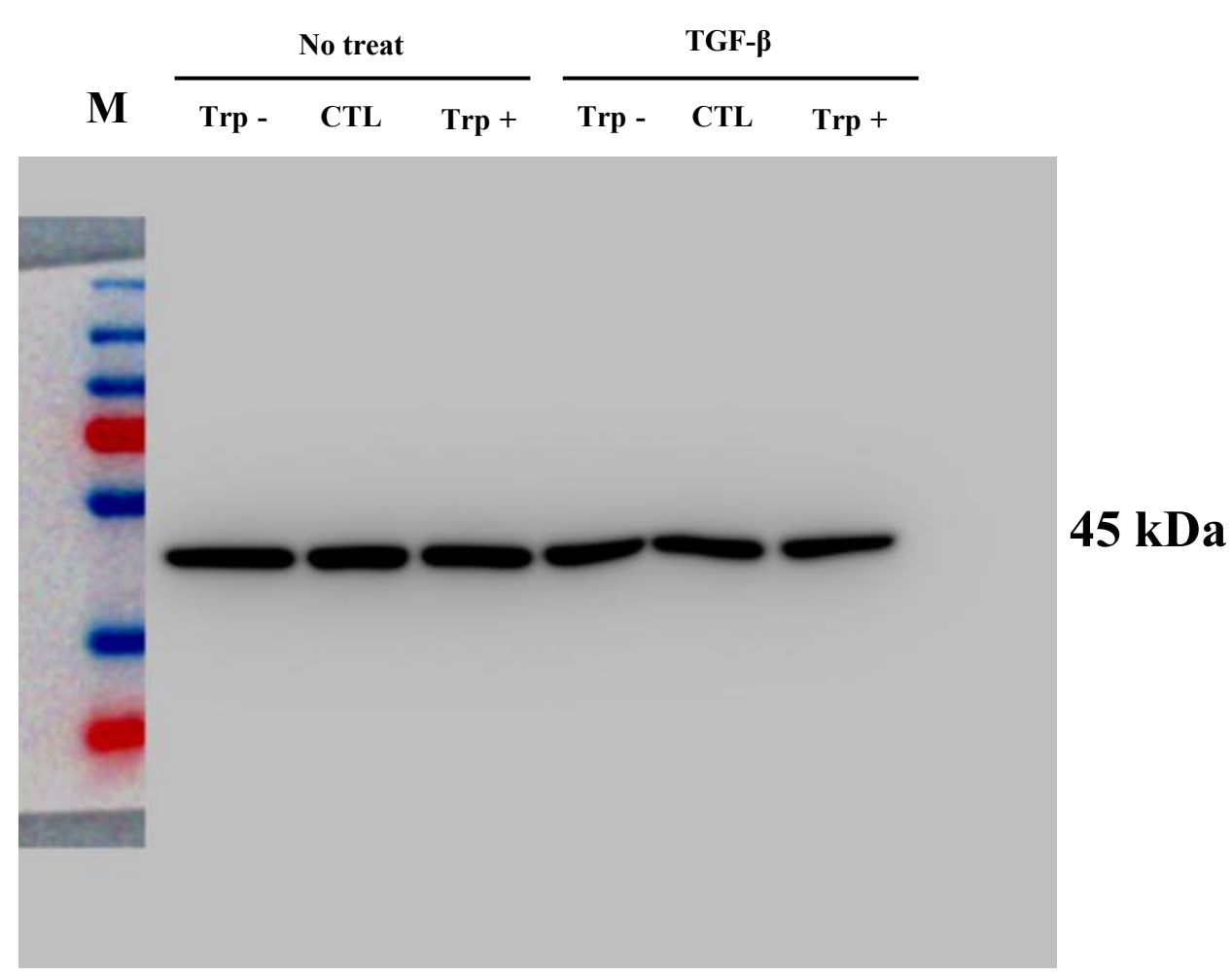

**Fig. 4C. TPH-1 and β-actin expression in human lung samples. Original uncropped chemiluminescence images.**

The loading order and identity of the experimental groups are indicated above each image. Each lane represents an independent biological sample from either the normal control (NC) or idiopathic pulmonary fibrosis (IPF) group. The molecular weight marker lane is indicated by "M". The expected molecular weights of TPH-1 and β-actin are approximately 52 kDa and 45 kDa, respectively. Images were captured using a ChemiDoc™ Touch Imaging System (Bio-Rad Laboratories, Hercules, CA, USA).

**TPH-1**

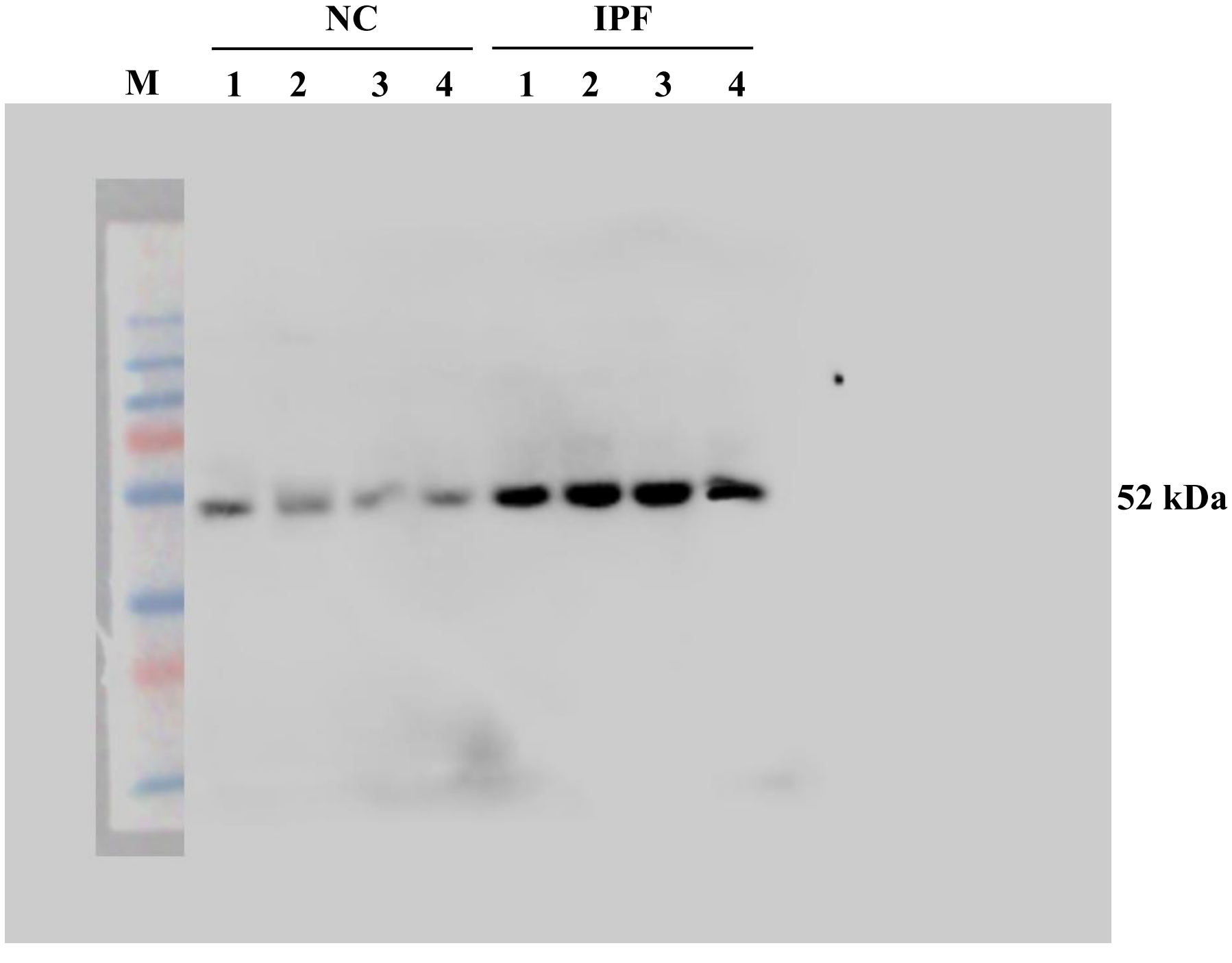


**β-actin**

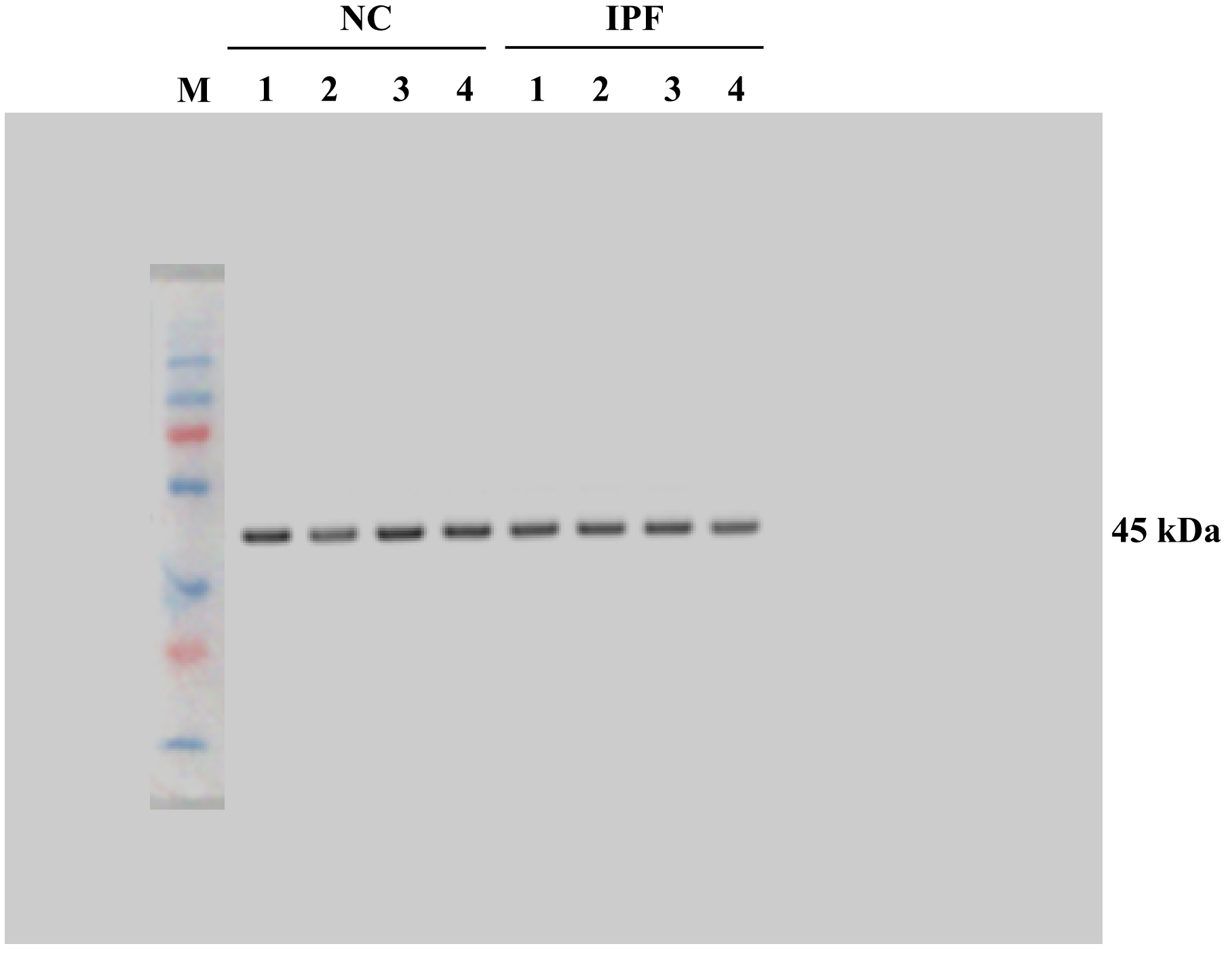

**Fig. 4C. TPH-1 and β-actin expression in control and bleomycin-treated mouse lungs. Original uncropped chemiluminescence images.**

The loading order and identity of the experimental groups are indicated above each image. Each lane represents an independent biological sample from either the control or bleomycin-treated (BLM) group. The molecular weight marker lane is indicated by “M”. The expected molecular weights of TPH-1 and β-actin are approximately 52 kDa and 45 kDa, respectively. Images were captured using a ChemiDoc™ Touch Imaging System (Bio-Rad Laboratories, Hercules, CA, USA).

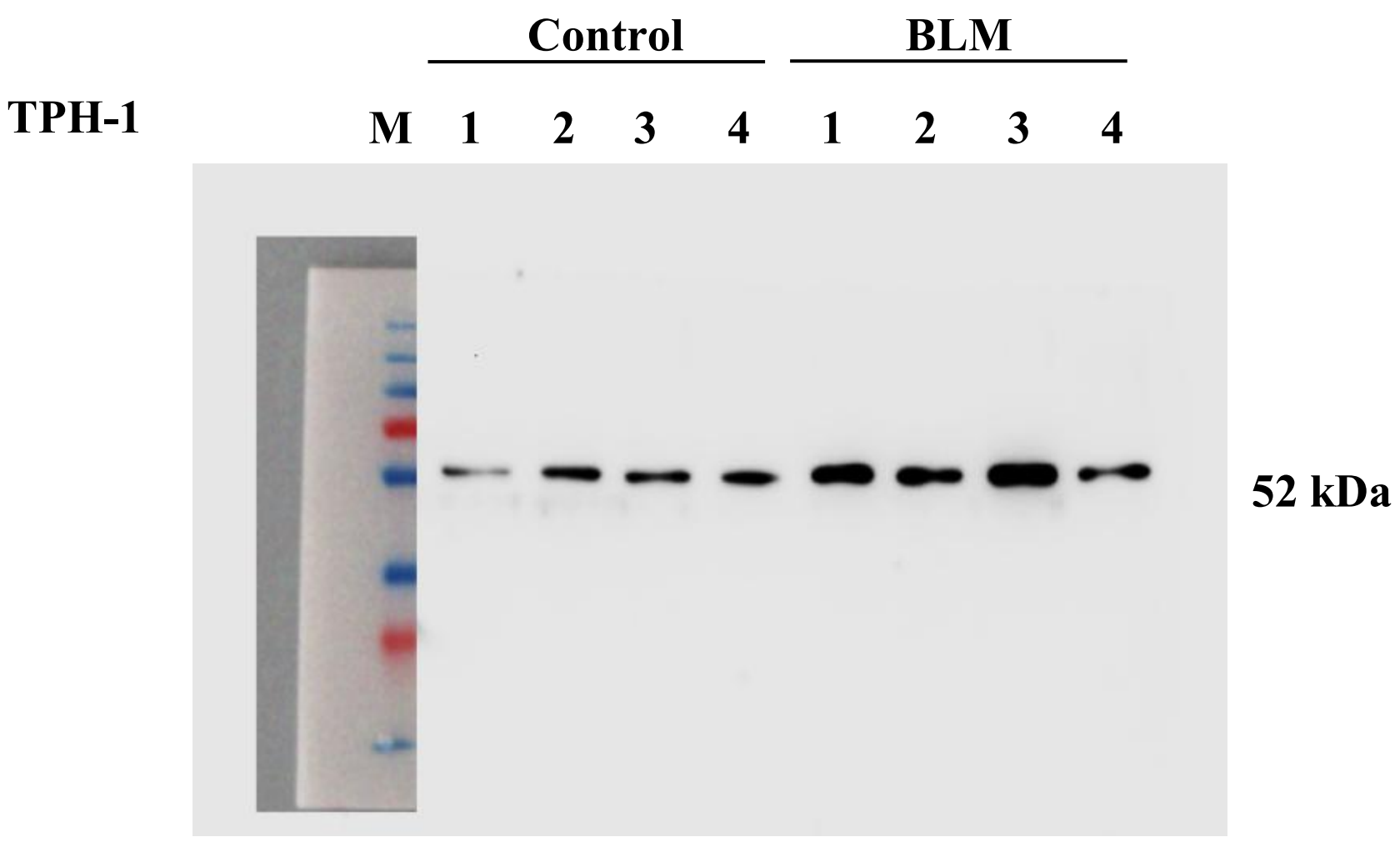


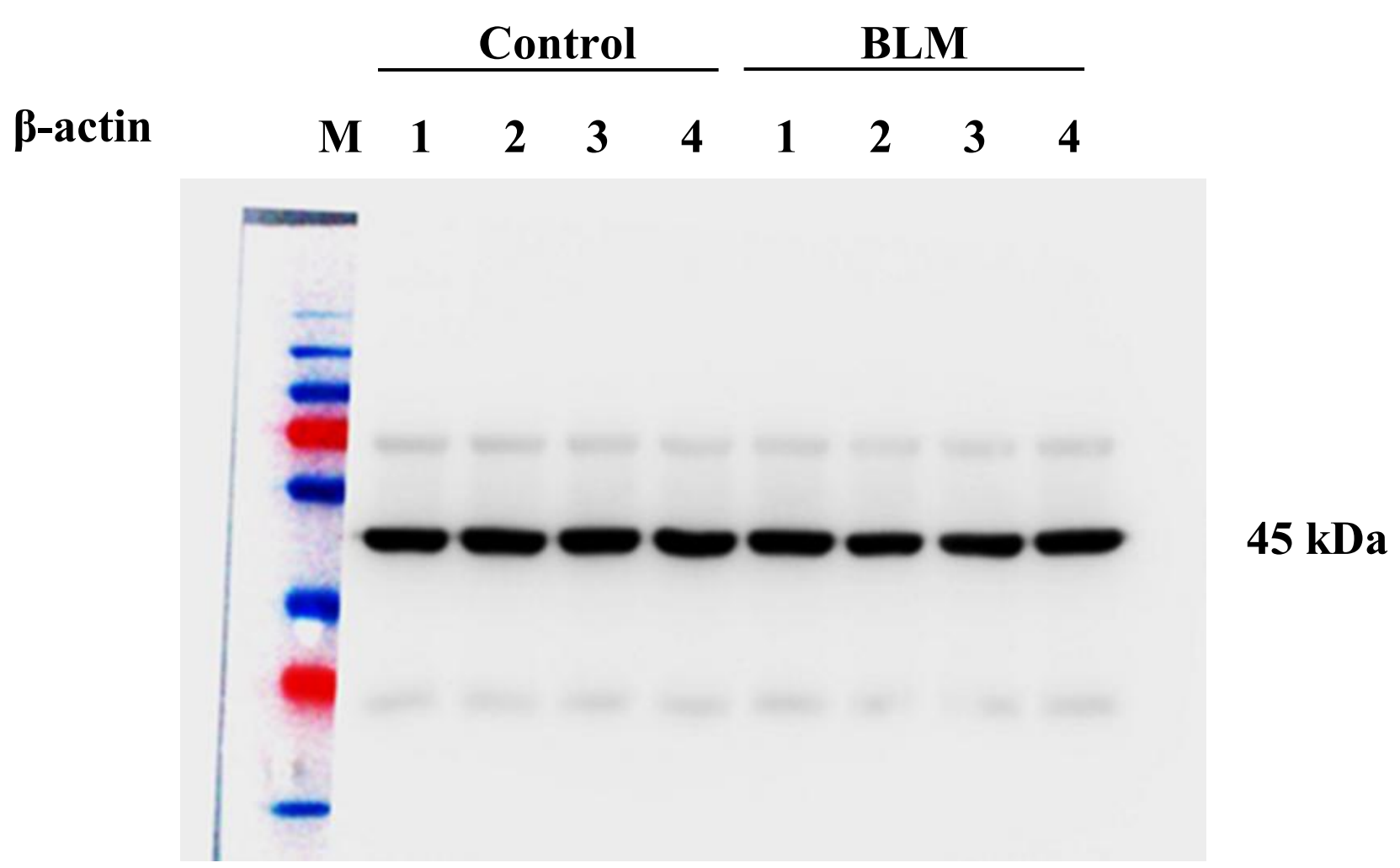

Supplement: Supplementary file 1 [file pone.0361463.s002.pdf]
