# Supplementary material for: Exogenous tryptophan aggravates experimental lung fibrosis through activation of AKT-mTORC1 pathway
Source: PLoS One. 2026 Nov 3;21(11):e0361463. doi: 10.1371/journal.pone.0361463 (PMC13730385; doi:10.1371/journal.pone.0361463)
Supplement: Supplementary file 2 [file pone.0361463.s001.docx]

Supplementary materials for

Exogenous tryptophan aggravates experimental lung fibrosis through activation of AKT-mTORC1 pathway

Jisu Hong. M.S. ^1*^, Yunha Nam. M.D^1*^, Jib Yong Jung M.D^1^. Jun Yeong Baek. B.D^2^, Eun Ji Park. B.D^2^, Yemin Choi. B.D ^2^, Shinhee Park M.D., Ph.D. ^1^. Ae-Rin Baek M.D., Ph.D. ^1^, Hun-Gyu Hwang M.D. ^1^, Junehyuk Lee M.D., Ph.D. ^1^, An-Soo Jang M.D., Do-Jin Kim M.D., Ph.D. ^1^, Ph.D. ^1^, Woo Jin Kim. M.D., Ph.D. ^3,^ Sung-Woo Park^,^ M.D., Ph.D. ^1†^

^1.^Division of Allergy and Respiratory Medicine, Department of Internal Medicine, Soonchunhyang University Bucheon Hospital, Bucheon, Korea.

^2^Department of Pathology, Soonchunhyang University Bucheon Hospital, Gyeonggi-Do, south Korea.

^3^Department of Internal Medicine Environmental Health Center Kangwon National University, Gangwondaehakgil, Chuncheon-si, Gangwon-do, South Korea

^* :^ Equally contributed as the first author

^†^ **Corresponding author:** Sung-Woo Park, M.D., Ph.D.

E-mail: [swpark@schmc.ac.kr](mailto:swpark@schmc.ac.kr)

S1 Fig. L-tryptophan increases proliferation of IPF fibroblasts in a dose- and time-dependent manner.

Proliferation of control and IPF fibroblasts was assessed following treatment with 1, 10, and 100 µM L-tryptophan using a water-soluble tetrazolium assay. IPF fibroblasts exhibited increased proliferation from 24 h after TRP treatment in a dose- and time-dependent manner. Data are presented as the mean ± SEM. At each time point, TRP-treated and untreated fibroblasts were compared separately within the control and IPF groups using the Mann–Whitney U test. *p < 0.05 vs. the corresponding untreated group.
